# Kit^lo^ hematopoietic stem cells exhibit distinct lymphoid-primed chromatin landscapes that enhance thymic reconstitution

**DOI:** 10.1038/s41467-025-61125-1

**Published:** 2025-07-04

**Authors:** Harold K. Elias, Sneha Mitra, Marina B. da Silva, Adhithi Rajagopalan, Brianna Gipson, Nicole Lee, Anastasia I. Kousa, Mohamed A. E. Ali, Simon Grassmann, Rhoshini Raghuraman, Xiaoqun C. Zhang, Susan DeWolf, Melody Smith, Hana Andrlova, Kimon V. Argyropoulos, Roshan Sharma, Teng Fei, Joseph C. Sun, Cynthia E. Dunbar, Christopher Y. Park, Christina S. Leslie, Avinash Bhandoola, Michael G. Kharas, Marcel R. M. van den Brink

**Affiliations:** 1 Molecular Pharmacology Program, Sloan Kettering Institute, Memorial Sloan Kettering Cancer Center, New York, NY USA; 2 National Institutes of Health (NIH), Bethesda, MD USA; 3 Program for Computational and Systems Biology, Sloan Kettering Institute, Memorial Sloan Kettering Cancer Center, New York, NY USA; 4 Department of Immunology, Sloan Kettering Institute, Memorial Sloan Kettering Cancer Center, New York, NY USA; 5 Department of Hematology and Hematopoietic Cell Transplantation, City of Hope National Medical Center, Duarte, CA USA; 6 Department of Pathology, New York University Grossman School of Medicine, New York, NY USA; 7 Leukemia Service, Department of Medicine, Memorial Sloan Kettering Cancer Center, New York, NY USA; 8 Division of Blood and Marrow Transplantation and Cellular Therapy, Department of Medicine, Stanford University School of Medicine, Stanford, CA USA; 9 Department of Pathology and Laboratory Medicine, Memorial Sloan Kettering Cancer Center, New York, NY USA; 10 Single-cell Analytics Innovation Lab, Sloan Kettering Institute, Memorial Sloan Kettering Cancer Center, New York, NY USA; 11 Department of Epidemiology and Biostatistics, Memorial Sloan Kettering Cancer Center, New York, NY USA; 12 Translational Stem Cell Biology Branch, National Heart, Lung, and Blood Institute, NIH, Bethesda, MD USA; 13 Laboratory of Genome Integrity, Center for Cancer Research, National Cancer Institute, NIH, Bethesda, MD USA

**Keywords:** Lymphopoiesis, Gene regulation in immune cells, Autoimmunity, Ageing

## Abstract

Hematopoietic stem cells (HSC) with multilineage potential are critical for T cell reconstitution after allogeneic hematopoietic cell transplantation (allo-HCT). The Kit^lo^ HSC subset is enriched for multipotential precursors, but their T cell potential remains poorly characterized. Using a preclinical allo-HCT mouse model, we demonstrate that Kit^lo^ HSCs provide superior thymic recovery and T cell reconstitution, resulting in improved immune responses to post-transplant infection. Kit^lo^ HSCs with augmented bone marrow (BM) lymphopoiesis mitigate age-associated thymic alterations and enhance T cell recovery in middle-aged mice. Mechanistically, chromatin profiling reveals Kit^lo^ HSCs exhibiting higher activity of lymphoid-specifying transcription factors, such as, ZBTB1. *Zbtb1* deletion diminishes HSC engraftment and T cell potential; by contrast, reinstating *Zbtb1* in megakaryocytic-biased Kit^hi^ HSCs rescues hematopoietic engraftment and T cell potential in vitro and in vivo. Furthermore, age-associated decline in Kit^lo^ HSCs is associated with diminished T lymphopoietic potential in aged BM precursors; meanwhile, Kit^lo^ HSCs in aged mice maintain enhanced lymphoid potential, but their per-cell capacity is diminished. Lastly, we observe an analogous human BM KIT^lo^ HSC subset with enhanced lymphoid potential. Our results thus uncover an age-related epigenetic regulation of lymphoid-competent Kit^lo^ HSCs for T cell reconstitution.

## Abstract (web summary)

Hematopoietic stem cells (HSCs) differentiate into multiple lineages, with such capacity impacted by aging. Here the authors identify Kit^lo^ HSCs as a functionally distinct population that exhibits distinct lymphoid-primed chromatin landscapes, which drive enhanced lymphoid reconstitution capacity, and is altered in aged hosts.

## Introduction

T lymphocytes play a crucial role in the adaptive immune response. A highly diverse T cell pool is required to recognize and eliminate foreign pathogens while also maintaining self-tolerance. The thymus continuously produces T cells, but with advancing age, its regenerative capacity declines^1^, leading to decreased thymic output and compromised T cell diversity^2–7^. Furthermore, the thymus is also vulnerable to acute damage from infections, cancer therapies, and conditioning regimens for allogeneic hematopoietic cell transplantation (allo-HCT), which results in prolonged T cell lymphopenia, thereby increasing the risk of infections and cancer relapse, contributing to transplant-related complications and mortality^8^. For these reasons, early T cell reconstitution is a positive prognostic indicator of allo-HCT outcomes^9,10^.

Post-transplant T cell reconstitution requires a steady supply of hematopoietic stem cells (HSCs)-derived lymphoid progenitors^11^ as well as thymic recovery. Prior research has demonstrated that thymic integrity is dependent on thymocyte-stromal crosstalk, particularly in the thymic epithelial cell (TEC) compartment^12–14^. Age-associated reduced lymphoid potential originating at the level of HSCs^15^, coupled with thymic decline^1^, contributes to delayed immune recovery. Therefore, identifying strategies to augment T lymphopoiesis and promote thymic regeneration is an unmet clinical need.

Single-cell transplantation with tracking and sequencing of progeny^16–21^ has uncovered heterogeneity in self-renewal capacity as well as lineage output among reconstituting HSCs- highlighting HSCs with multilineage versus lineage-biased potential. These studies showed that, while only a fraction of HSCs generate “balanced” multilineage output, the majority exhibit a diverse range of lineage potential following transplantation- varying in their contributions and reconstitution kinetics for each lineage, thereby highlighting their inherent biases. These lineage biases are intrinsically stable and maintained following transplantation^22–24^. At the molecular level, these cell-autonomous features are mediated by epigenetic configuration and are divergent from their transcriptional state^19,25^. Although this suggests the existence of a highly organized and predictable framework for lineage-restricted fates of long-term self-renewing HSCs, the molecular identities, gene regulatory networks, and functional implications of these differences governing lymphoid fate decisions remain unexplored.

Kit^lo^ HSCs have previously been described to exhibit better self-renewal and multipotency, including improved T cell reconstitution in syngeneic transplant models^26,27^. However, their thymic reconstituting ability, age-related changes, as well as the molecular basis for their enhanced T cell lymphoid potential are unknown. Here, we delineate the molecular mechanism underlying enhanced lymphoid potential in multilineage Kit^lo^ HSCs, identifying ZBTB1 as a critical transcription factor that governs their superior T cell reconstitution capacity. We demonstrate that the frequency of the Kit^lo^ subset declines with age and establish the pivotal role of these HSCs in orchestrating thymic recovery and immune reconstitution in both young and aged hosts. Through functional studies, we establish that ZBTB1 expression directly correlates with improved hematopoietic engraftment and thymic recovery following transplantation and is sufficient to rescue the lymphoid defect in aged HSCs. We further identify a corresponding human HSC subset with analogous enhanced lymphoid characteristics, demonstrating conservation of this marker to identify lymphoid-competent HSCs across species. This work builds on prior studies on lineage specification programs in HSCs and advances our fundamental understanding of potential molecular underpinnings governing lymphoid-primed HSCs. The identification of enhanced lymphoid-potential HSC subsets in human bone marrow, combined with emerging ex vivo expansion techniques, offers promising translational opportunities for improving immune regeneration after bone marrow transplantation while counteracting treatment-related immunosuppression and age-associated thymic decline.

## Results

### Reduced lymphoid output in aged HSCs impairs thymic recovery

Previous studies have shown impaired lymphoid reconstitution with aged HSCs in a syngeneic transplant model, yet this has not been explored using an allogeneic setting. We used a preclinical allo-HCT model to analyze immune reconstitution of young and old donors. To evaluate if HSCs with differences in lymphoid progenitor production could impact thymic recovery, we transplanted LT HSCs (Long-Term Hematopoietic Stem Cells, Lineage^−^CD34^−^CD48^−^CD150^+^Sca-1^+^cKit^+^) cells from young adult (2-mo) or old (24-mo) C57BL/6 mice with rescue BM cells (CD45.1) into lethally irradiated young BALB/cJ recipients (Supplementary Fig. 1A). Because HSC-derived thymic progeny emerges within 5–6 weeks^28^, we established an 8-week harvest timepoint. This interval allowed sufficient time for HSC-derived progenitors to repopulate the thymus, thereby providing a better understanding of thymic regeneration dynamics and the efficacy of HSC transplantation in promoting thymic reconstitution.

Consistent with prior reports, eight weeks post-HCT, we found that young HSCs supported balanced lineage reconstitution, while old HSCs exhibited significantly impaired T cell reconstitution in the peripheral blood (PB), despite comparable reconstitution patterns at four weeks (Supplementary Fig. 1B–E). Young HSCs demonstrated superior multilineage potential, particularly lymphoid reconstitution (LMPP/MPP4: lymphoid-primed multipotent progenitors and CLP: common lymphoid progenitors, Supplementary Fig. 1F, G), compared to old HSCs. Furthermore, recipients of young HSCs demonstrated significantly greater thymic cellularity and enhanced reconstitution of precursor and mature thymocytes compared to old HSC recipients (Supplementary Fig. 1H, I). In conclusion, old HSCs have significantly less multilineage potential, but especially T lymphoid potential, than young HSCs in young allo-HCT recipients.

### Age-related decline in multilineage HSCs marked by low Kit expression

Recent reports have shown remarkable functional heterogeneity of HSC subsets^29–34^, highlighting discordance between current HSC phenotypic definitions to their molecular identities^17–19^. To identify HSCs with an enriched multilineage program and gain insights into their transcriptional and epigenetic framework, we performed multiome single-cell RNA and ATAC sequencing on hematopoietic stem cells LT HSCs from 2-mo (young) or 24-mo (old) female mice.

To define HSC subsets, we performed unsupervised Leiden cluster analysis on the multiome scRNA-seq data from our young cohort (Supplementary Fig. 2A, B). Using nomenclature and signatures derived from previous studies^16,17,19,35–38^, we mapped all unsupervised clusters into five main subsets (Supplementary Fig. 2C): Quiescent HSC (q-HSC) exhibited a low-output signature (*Cdkn1c, Mpl*, and *Socs2*) and shared transcriptional features with megakaryocytic-biased HSCs (Mgk-HSC), marked by elevated expression of stemness-associated genes including *Mllt3, Hlf, Mecom, Txnip, Ifitm1, Msi2, Procr, Hoxa9, Ogt, Pim1*, and *Satb1*. Multilineage HSCs (MLin-HSCs) were characterized by higher expression of multipotency genes (*Zeb2, Pbx1, Runx3*, and *Tgfb1*) and shared molecular signatures with proliferative HSCs (p-HSCs), including cell cycle regulators *CDK6, Pola1, Hells*, and *Itga2b*. Notably, we identified a novel Intermediate HSC population (Int-HSC) that displayed overlapping molecular features with Mgk-HSC, p-HSCs, and MLin-HSCs, distinguished by elevated expression of *Stat5b, Jak2, Cdc42*, and *Ywhaz* (related to LNK-JAK2 interaction). (Supplementary Data 1 and Supplementary Fig. 2D).

**Fig. 1:**
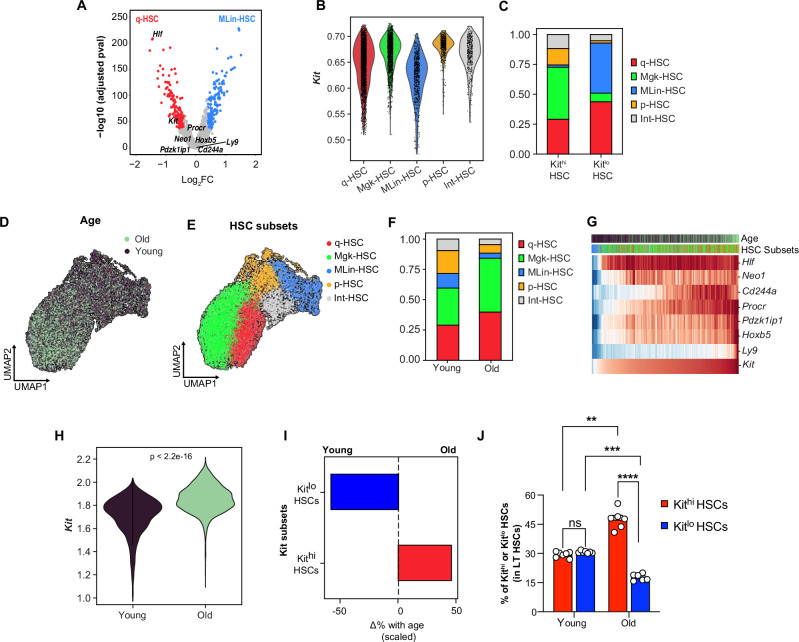
Age-related decline in multilineage HSCs marked by low Kit expression. **A**–**H** Multiome single-cell RNA and ATAC sequencing was performed on HSC (Lineage-Sca-1+cKit+ CD34-CD48-Flt3-CD150+) cells isolated from 2-mo (young) or 24-mo (old) female C57BL/6 mice. nTOTAL = 12,350 cells. **A** Volcano plot of DGE analysis between MLin-HSC vs. q-HSC subsets in young HSCs, highlighting bona fide HSC markers. **B** Violin plot for imputed *Kit* gene expression by annotated HSC subsets (as described in Fig. S2C). **C** Frequency of computationally defined HSC subtypes in Kit^hi^ and Kit^lo^ subsets in young HSCs. **D**, **E** Combined Uniform manifold approximation and projection (UMAP) of young and old HSCs after Harmony batch correction, annotated by age (**D**) and annotated HSC subsets (q-HSC: Quiescent HSCs; Mgk-HSCs: Platelet-biased HSCs; Mlin-HSC: Multilineage HSCs; p-HSC: Proliferative HSCs; Int-HSC: Intermediate HSCs) (**E**). **F** Frequency of HSC subtypes in young and old HSCs. **G** Heatmap showing MAGIC-imputed gene expression values of bona fide HSC markers ordered by increasing Kit expression. **H** Violin plot for imputed Kit gene expression by age. Statistical analysis was performed using the Wilcoxon test. **I** Scaled change in frequency for each Kit HSC subset with age. **J** FACS analysis showing the frequency of Kit^hi^ and Kit^lo^ HSCs by age. All data are from *n* = 6 mice/group (young = 6; old = 6). Error bars represent mean ± SEM. **P < 0.01, ***P < 0.001. *P*-values calculated by two-way ANOVA. Source data are provided as a Source Data file, Source Data Fig. 1.

To identify HSCs with multilineage potential, we compared multilineage enriched MLin-HSC versus low-output q-HSC subsets in young HSCs and queried for previously described bona fide HSC markers (*Hlf, Kit, Neo1, Procr, CD244a, Pdzk1ip1, Ly9, Hoxb5*). *Hlf* and *Kit* had significantly higher expression in q-HSCs compared to MLin-HSCs (Fig. 1A). *Hlf* has been previously implicated in HSC quiescence^39–41^, whereas *Kit* enriches for platelet-biased HSCs^27^. To further analyze the composition of HSCs with varying Kit expressions, we defined Kit high and low HSC populations (Kit^hi^ and Kit^lo^ HSCs) based on the top and bottom 20% of *Kit* gene expression levels. We observed lower Kit expression in MLin-HSCs (Fig. 1B) and increased representation of q-HSC and MLin-HSCs within the Kit^lo^ HSCs (Fig. 1C and Supplementary Fig. 2E), consistent with prior reports of increased self-renewal capacity and multipotency in Kit^lo^ HSCs^26,27^.

To validate these RNA-based findings at the protein level and confirm the relationship between Kit abundance and HSC subset distribution, we analyzed a CITE-seq dataset generated on young LSKs (GSE243197)^42^. We defined Kit high and low HSC populations (Kit^hi^ and Kit^lo^ HSCs) based on the top and bottom 30% of CD117 antibody-derived tag (ADT) reads, mirroring our gating thresholds to fractionate Kit HSC subsets. Consistent with our transcriptional analysis, we observed an increased representation of q-HSC and MLin-HSCs within the Kit^lo^ HSCs, while Kit^hi^ HSCs were enriched for Mgk-HSC, p-HSC, and Int-HSCs (Supplementary Fig. 2F and G).

We then sought to understand how Kit corresponds to previously defined HSC-lineage-biased markers (CD229, CD61, CD150, and Neogenin), which are known indicators of lineage bias. While CD150^lo^ and CD229+ mark lymphoid-biased HSCs^23,43^, Neogenin+ identifies myeloid bias^44^, and CD61+ expression denotes quiescent HSCs^45^. We observed that Kit^lo^ HSCs express lymphoid-biased markers CD150^lo^ (Supplementary Fig. 3A–C) and CD229 (Supplementary Fig. 3G, H), though these markers significantly underrepresent Kit^lo^ HSCs (CD229+: ~10%; CD150lo: ~40%). In contrast, Kit^lo^ HSCs showed low expression of the myeloid-biased marker Neo1 (Neo1+: ~25%, Supplementary Fig. 3D–F). Additionally, CD61-expressing HSCs are unable to distinguish Kit HSC subsets. (Supplementary Fig. 3G, H).

To assess for age-associated changes, we first compared the transcriptional profile of young and old HSCs. In our differential gene analysis, we found that HSCs from young mice had higher expression of genes related to cell cycle (*Ccnd3*, *Cdc25a*, *Cdk1)*, and cell polarization (*Arhgap15*); conversely, old HSCs were enriched for expression of genes related to quiescence (*Egr1*, *Junb, Jun*, *Jund*, *Fos*), platelet differentiation (*vWf*, *Clu*, *Itgb3, Fhl1 and Tgm2*), and myeloid differentiation (*Selp, Neo1*) (Supplementary Data 2). To investigate age-related changes in HSC populations, we used our young HSC data as a reference to classify the HSC subtypes in old HSCs (Supplementary Fig. 2H). Subsequently, we generated a UMAP of our young and old datasets (Supplementary Fig. 2I and Fig. 1D) to visualize the post-ingestion results (Fig. 1E). While all HSC subsets were present in young and old cohorts (Fig. 1F), we notably observed an increased representation of MLin-HSCs, p-HSCs, and Int-HSCs in young HSCs. Conversely, q-HSCs and Mgk-HSCs were predominantly in old HSCs (Supplementary Fig. S2J). Consistent with previous studies^19,30,46^, these findings suggest shifts in the composition of HSC subsets with aging, indicating a reduction in multilineage potential accompanied by an increase in platelet bias.

Given the increased frequency of Mgk-HSCs in old HSCs and prior work^27^ demonstrating that Kit^hi^ HSCs are platelet-biased HSCs (Supplementary Fig. 2E, J, and L), we hypothesized this could be attributed to an expansion in Kit^hi^ HSCs. We observed significantly higher *Kit* expression (Fig. 1G, H and Supplementary Fig. 2M) in old HSCs, with an age-dependent reduction in the proportion of Kit^lo^ HSCs, accompanied by an increase in the frequency of Kit^hi^ HSCs (Fig. 1I). Flow cytometry analysis of young and old bone marrow confirmed a significant expansion of Kit^hi^ HSCs, accompanied by a concomitant decrease in Kit^lo^ HSCs in old HSCs (Supplementary Fig. 2N and Fig. 1J), consistent with our prior report^47^. Together, these findings indicate that the frequency of multilineage Kit^lo^ HSCs declines with age.

### Young Kit^lo^ HSCs exhibit enhanced T cell potential

**Fig. 2:**
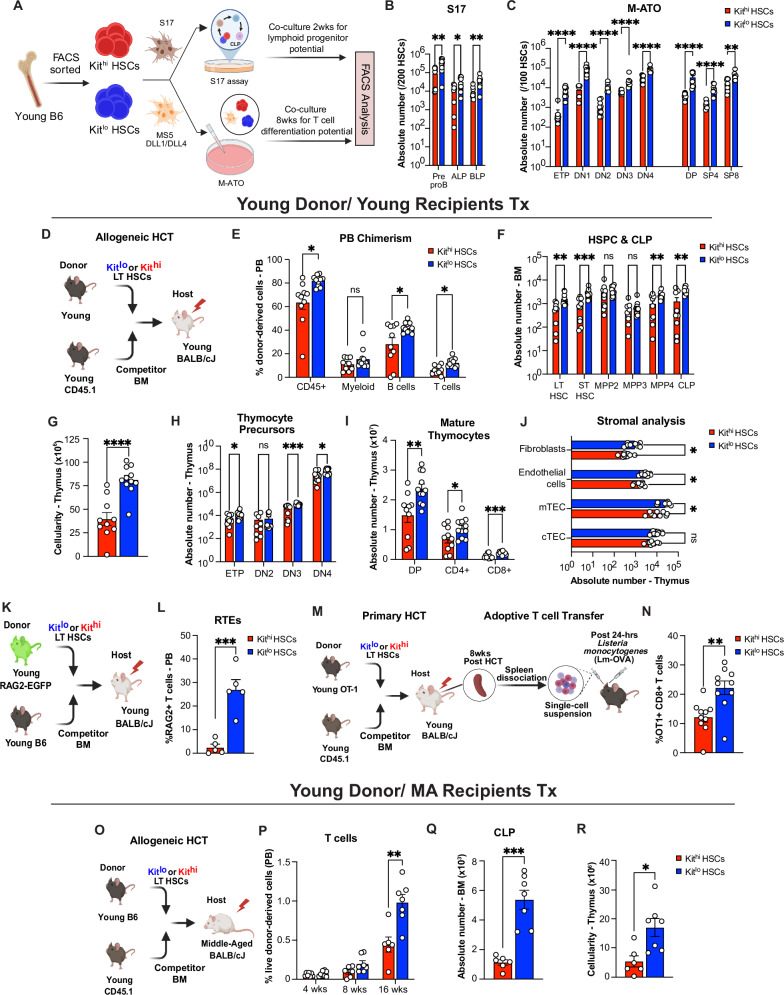
Young Kit^lo^ HSCs exhibit enhanced T cell potential. **A** Experimental schema to evaluate in vitro lymphoid progenitor and T cell differentiation potential of Kit^hi^ (red) and Kit^lo^ HSCs (blue) from 2-mo (young) C57BL/6 mice using the S17 lymphoid assay and murine artificial thymic organoid (M-ATO), respectively. Enumeration of absolute number of lymphoid progenitor cells following 14 days in S17 lymphoid assay (**B**) and absolute number of T cell subsets following 8 weeks of culture in M-ATOs (**C**). Refer to Supplementary Fig. 13A, B for gating strategies to define the above populations. Aggregated data across 3 independent experiments, each performed in triplicate (Kit^lo^ = 3; Kit^hi^ = 3). **D** Experimental schema for competitive allogeneic HCT (allo-HCT) using Kit^hi^ (red) and Kit^lo^ HSCs (blue) from 2-mo (young) C57BL/6 mice with competitor bone marrow (BM) cells from B6.SJL-PtprcaPepcb/BoyJ mice were transplanted into lethally irradiated 7-week-old (young) BALB/cJ recipients. **E**–**J** Eight weeks after competitive HCT, (**E**) frequency of donor-derived chimerism (CD45.2/ H-2Kb) of mature lineages in the peripheral blood (PB, CD45+, Myeloid cell: Gr-1+CD11b+; B cell: B220+; T cell: CD3 + ), (**F**) Enumeration of absolute number of donor-derived cells in the BM for LSK cells (LT HSC: Lineage^−^Sca-1^+^cKit^+^ CD34^−^CD48^−^Flt3^−^CD150^+^**;** ST HSC: Lineage^−^Sca-1^+^cKit^+^ CD48^−^Flt3^−^CD150^−^**;** MPP2: Lineage^−^Sca-1^+^cKit^+^ CD48^+^Flt3^−^CD150^−^; MPP3: Lineage^−^Sca-1^+^cKit^+^ CD48^+^Flt3^−^CD150^+^; MPP4/ LMPP: Lineage^−^Sca-1^+^cKit^+^ Flt3^+^CD150^−^) and Common Lymphoid Progenitors cells (CLP: Lineage^−^IL7Ra^+^Flt3^+^Sca^mid/lo^ Kit^lo^). **G**–**J** Post-HCT thymi analysis for total thymic cellularity (**G**), **H**, **I** enumeration of absolute number of donor-derived cells for T cell precursors (ETP: Lineage^−^ CD4^−^ CD8^−^CD44^+^ CD25^−^Kit^+^; DN2: Lineage^−^ CD4^−^ CD8^−^CD44^+^ CD25^+^; DN3: Lineage^−^ CD4^−^ CD8^−^CD44^−^ CD25^+^) (**H**), Mature T cells (DP: Lineage^−^ CD4^+^ CD8^+^; SP4: Lineage^−^ CD4^+^ CD8^−^; SP8: Lineage^−^ CD4^−^ CD8^+^) (**I**), and analysis of thymic CD45- compartment for Thymic Epithelial Cells (TEC: CD45^−^ EpCAM^+^), Cortical TEC (cTEC: CD45^−^ EpCAM^+^UEA-1^lo^ 6C3^hi^ MHCII^hi/lo^), Medullary TEC (mTEC: CD45^−^ EpCAM^+^UEA-1^hi^ 6C3^lo^ MHCII^hi/lo^^−^), endothelial cells: CD45^−^ EpCAM^−^Ter119 PDGFRα^−^ CD31^+^ and fibroblasts: CD45^−^ EpCAM^−^Ter119^−^CD31^−^ PDGFRα^+^) (**J**). Data for (**E**–**I**) are from *n* = 10–11 mice/group (Kit^hi^ = 10; Kit^lo^ = 11) and (**J**) from 9 mice/group (Kit^hi^ = 9; Kit^lo^ = 9), across two independent experiments. **K** Experimental schema to evaluate thymic function following competitive allo-HCT using young HSCs. **L** Frequency of donor-derived Recent Thymic Emigrants (RTE: CD3 + GFP + ) in the PB at 8 weeks post-HCT. Refer to Supplementary Fig. 13C for the gating strategy to define the above population. All data are from *n* = 5 mice/group (Kit^lo^ = 5; Kit^hi^ = 5). **M** Experimental schema to investigate the functional response of differential Kit-expressing HSC-derived T cells in secondary recipients to *L. monocytogenes* expressing chicken ovalbumin (LM-OVA) infection. **N** Spleen analysis of secondary recipients for frequency of OT1-derived chimerism of CD8+ T cells (OT1+CD8 + : CD45.1^+^H2-K^b^CD8^+^) 7 days post-infection. Refer to Supplementary Fig. 13D for the gating strategy to define the above population. All data are from *n* = 10 mice/group, (Kit^lo^ = 10; Kit^hi^ = 10), across two independent experiments. **O** Experimental schema for competitive allogeneic HCT (allo-HCT) using Kit^hi^ (red) and Kit^lo^ HSCs (blue) from 2-mo (young) C57BL/6 mice with competitor bone marrow (BM) cells from B6.SJL-PtprcaPepcb/BoyJ mice transplanted into lethally irradiated 14-mo (Middle-aged) BALB/cJ recipients. **P** Frequency of donor-derived T cell chimerism at the indicated timepoints. **Q**, **R** Sixteen weeks after competitive HCT, enumeration of donor-derived CLPs (**Q**) and total thymic cellularity (**R**). Data for (**P**–**R**) are from *n* = 6–7 mice/group, (Kit^lo^ = 7; Kit^hi^ = 6), across two independent experiments. Refer to Supplementary Fig. 12 for gating strategies to define the above populations. Error bars represent mean ± SEM. *P < 0.05, **P < 0.01, ***P < 0.001, ****P < 0.0001. *P*-values calculated by a nonparametric unpaired two-tailed Mann–Whitney U test. Panels **A**, **D**, **K**, **M**, and **O** were *created in BioRender. Lab, K. (2025)* https://BioRender.com/fl4hwgn*and* https://BioRender.com/oeh4i7x. Source data are provided as a Source Data file, Source Data Fig. 2.

Next, we sought to determine the lymphoid potential of the HSC Kit subsets. To this end, we used the S17 lymphoid progenitor assay^48^ to assess for lymphoid progenitor output of purified Kit^hi^ and Kit^lo^ HSCs from young mice (Fig. 2A). Prior studies have elucidated the existence of functional heterogeneity among phenotypic Common Lymphoid Progenitors (CLPs) characterized by Ly6D expression^49^, resulting in the identification of two distinct subsets: All Lymphocyte Progenitors (ALPs), also referred to as functional CLPs, and B cell-biased Lymphocyte Progenitors (BLPs). Following a 12-day co-culture, we observed that young Kit^lo^ HSCs generated more ALPs and BLPs than Kit^hi^ HSCs (Fig. 2B). To further examine their T cell differentiation potential, we used murine artificial thymic organoids (M-ATO)^50–52^ (Fig. 2A). Following an 8-week culture, we noted a substantial increase in overall ATO output from young Kit^l^^o^ HSCs in comparison to Kit^hi^ HSCs (Fig. 2C).

To further evaluate the reconstituting potential of Kit^lo^ HSCs, we competitively transplanted Kit^lo^ or Kit^hi^ HSCs from young C57BL/6 (CD45.2) mice with rescue BM cells (CD45.1) into lethally irradiated young BALB/cJ recipients (Fig. 2D). Eight weeks after allo-HCT, we found that Kit^lo^ HSCs generated significantly better B and T cell reconstitution, while myeloid reconstitution was comparable (Fig. 2E, Supplementary Fig. 4A, B). Concordant with a prior report of better self-renewal ability^27^, we found that Kit^lo^ HSCs demonstrated increased LT HSC reconstitution (Fig. 2F). In support of multilineage potential, we observed that Kit^lo^ HSC recipients had higher lymphoid progenitor reconstitution (including LMPP and CLP, Fig. 2F, G), along with comparable myeloid precursor output (including MPP3: Multipotent Progenitor 3, CMP: Common Myeloid Progenitor, GMP: Granulocyte-Monocyte Progenitor, MEP: Megakaryocyte-Erythrocyte Progenitor, Fig. 2F and Supplementary Fig. 4C).

To evaluate whether enhanced lymphoid precursor output could contribute to thymic reconstitution, we concurrently evaluated the recipient thymi. We found that Kit^lo^ recipients demonstrated enhanced thymic recovery, characterized by significantly higher thymic cellularity and reconstitution of precursor and mature thymocytes (Fig. 2H–I). Notably, in support of a critical role for thymocyte-stromal crosstalk in preserving thymic architecture^12–14^, we also found higher thymic epithelial cells (TECs), specifically medullary TECs, and endothelial cell recovery in Kit^lo^ reconstituted thymi (Fig. 2J). We next examined donor-derived lymphoid progenitors for differential expression of molecules involved in homing to the thymus (CCR7, CCR9, and PSGL1)^53^. We observed significantly higher expression of CCR7 on Kit^lo^-derived lymphoid progenitors (CLP) (Supplementary Fig. 4D). In addition, we analyzed thymic supernatants and found no differences in levels of thymopoietic ligands, IL22^54^ and RANKL^55^ (Supplementary Fig. 4E). Finally, we found significantly higher secondary lymphoid organ (spleen) reconstitution in Kit^lo^ recipients (Supplementary Fig. 4F-G). To evaluate long-term reconstitution, we performed additional harvests at 20 weeks following HCT. We observed similar trends in higher BM lymphopoiesis, thymic reconstitution, and thymic stromal recovery in Kit^lo^ recipients (Supplementary Fig. 5A–H).

Having established Kit^lo^ HSC’s superior thymic reconstitution capacity, we next investigated how this HSC subset with enhanced lymphoid potential compares to previously defined lymphoid-biased HSC populations. Our immunophenotypic characterization studies of Kit^lo^ HSCs (Supplementary Fig. 3) revealed modest enrichment of lymphoid-biased CD229+ HSCs (~10%). In comparison, we observed a 40% enrichment for a previously described lymphoid-biased, CD150^lo^ HSCs^23^, but their relative contribution to thymic reconstitution compared to Kit^lo^ HSCs has not been directly assessed. Thus, we generated mix chimeras of Kit^lo^ HSCs and CD150^lo^ HSCs in an equal competition transplant (Supplementary Fig. 6A, B). We observed significantly better T cell and thymic reconstitution with Kit^lo^ HSCs compared to CD150^lo^ HSCs in both PB and thymus (Supplementary Fig. 6C, G, H). To determine whether these markers identify overlapping or distinct HSC populations with potential synergistic effects on lymphoid potential, we performed competitive transplants of Kit^lo^CD150^lo^ HSCs versus CD150^lo^ HSCs chimeras. These studies revealed better T cell and thymic repopulating potential of Kit^lo^CD150^lo^ HSCs (Supplementary Fig. 6C, G, H), suggesting Kit expression provides additional lymphoid potential beyond that conferred by low CD150 expression alone. Finally, to investigate whether Kit^lo^ better identifies multipotent HSCs within myeloid-biased CD150^hi^ HSCs, we examined our CD150^hi^-CD150^hi^Kit^lo^ chimeras (Supplementary Fig. 6C, G, H). In line with prior observations, we observed that, while CD150^hi^ HSCs exhibit decreased T cell output compared with CD150^lo^ HSCs, CD150^hi^Kit^lo^ HSCs possess superior thymic repopulating potential compared to CD150^hi^ HSCs (Supplementary Fig. 6C), revealing a previously unrecognized functional heterogeneity within traditionally myeloid-biased HSC populations. We observed similar trends in B cell reconstitution across Kit^lo^ and CD150^lo^ HSCs (Supplementary Fig. 6D), though not reaching statistical significance. In contrast,  CD150^hi^ HSC subsets exhibited robust myeloid reconstitution compared to Kit^lo^ and CD150^lo^ HSC subsets, consistent with their myeloid-biased potential (Supplementary Fig. 6E). Notably, the improved thymocyte repopulation with CD150^lo^Kit^lo^ and Kit^lo^ HSCs was reflected in superior bone marrow lymphopoiesis with increased LMPP and CLP compared to CD150^lo^ HSCs (Supplementary Fig. 6F), while the enhanced thymocyte repopulating potential of CD150^hi^Kit^lo^ HSCs compared to CD150^hi^ HSC subsets, despite comparable CLP reconstitution (Supplementary Fig. 6F), is consistent with previously described CLP-independent pathways of T cell differentiation^56,57^.

To assess thymic output, we used the RAG2-GFP model^58^, which allows the analysis of recent thymic emigrants (RTEs) (Fig. 2K). We observed higher numbers of PB GFP + T cells in Kit^lo^ vs Kit^hi^ recipients (Fig. 2L). Additionally, to assess whether this increased number of PB T cells in Kit^lo^ recipients results in increased functionality (Fig. 2M), we analyzed response to ovalbumin engineered *Listeria Monocytogenes* (LM-OVA) and observed a significant increase in OT1+CD8+ T cells (Fig. 2N).

A key element in the post-HCT recovery of thymic function is the influx of bone marrow-derived lymphoid progenitors, also known as thymic seeding progenitors (TSPs)^11^. Furthermore, the significance of restoring the bone marrow-thymus axis in mitigating thymic involution has been underscored through various strategies aimed at augmenting BM lymphopoiesis, including sex-steroid ablation^59,60^ and administration of Ghrelin^61^, among other approaches^62^. We therefore hypothesized that robust BM lymphopoiesis driven by Kit^lo^ HSCs could potentially counteract age-related alterations within the thymic microenvironment, consequently ameliorating the decline in T cell output. To this end, we competitively transplanted Kit^lo^ or Kit^hi^ HSCs from young C57BL/6 mice (CD45.2) with rescue BM cells (CD45.1) into lethally irradiated 14-month-old (Middle-aged) BALB/cJ recipients (Fig. 2O). Sixteen weeks post-transplantation, we observed significantly higher numbers of peripheral T and B cells in Kit^lo^ recipients (Fig. 2P and Supplementary Fig. 4I, J). This was attributed to increased numbers of Kit^lo^ HSC-derived lymphoid progenitors (including LMPP/MPP4 and CLPs, Supplementary Fig. 4J and Fig. 2Q), while numbers of myeloid precursors were comparable (MPP3, CMP, GMP, and MEP, Supplementary Fig. 4J, K). In our parallel analysis of the thymi of Kit^lo^ vs Kit^hi^ recipients, we observed increased numbers of thymocytes and TECs (Fig. 2R and Supplementary Fig. 4L–N). Collectively, these findings establish the potential of Kit^lo^ HSCs for lymphopoiesis and to improve thymic recovery and peripheral T cell reconstitution in recipients of various ages.

### Kit^lo^ HSCs exhibit distinct epigenetic features that facilitate lymphoid potential

**Fig. 3:**
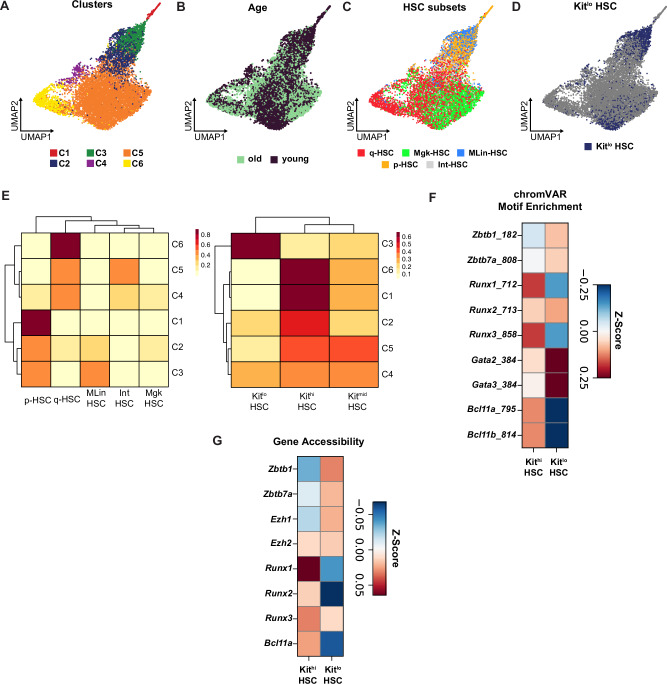
Kit^lo^ HSCs exhibit distinct epigenetic features that facilitate lymphoid potential. **A** UMAP of 6 unsupervised HSC clusters identified by tile-based scATAC-seq analysis after Harmony batch correction. **B**–**D** Annotated by age (**B**), HSC subsets (**C**), and Kit subsets (**D**) on the scATAC-seq UMAP. **E** scATAC-seq cluster composition by HSC subsets (left) and Kit subsets (right). Color scale denotes correlation values between the ATAC clusters and cell annotations, generated using ArchR. **F**, **G** Matrix plots showing motif enrichment identified by ChromVAR (**F**) and gene accessibility (**G**) for lymphoid-specifying transcription factors for Kit subsets in young and old HSCs. Z-scores denote chromVAR motif enrichment in (**F**) and gene accessibility scores (peak matrix values) that have been row-scaled in (**G**), generated using ArchR.

Prior work has shown that immunophenotypically defined HSC clones with differential lymphoid output have distinct epigenetic signatures, with no discernible patterns in terms of gene expression^19,25,63^. To identify the gene regulatory network orchestrating the lymphoid potential differences in HSCs, we analyzed our single-cell ATAC-seq (scATAC-seq) component from our multiome dataset (Supplementary Fig. 7A–D). Through unsupervised clustering, we identified 6 clusters, including young and old HSCs (Fig. 3A, B). Distinct HSC subsets identified via scRNA-seq analyses (Fig. 1B) are situated in varied regions on the scATAC-seq UMAP (Fig. 3C, D). Notably, MLin-HSC and Kit^lo^ HSC intersect with Cluster 3 from the scATAC-seq clusters (Fig. 3E).

We characterized the differentially accessible regions (DARs) of chromatin that defined the HSC subsets and found 2816 DARs in cluster C3 (MLin-HSC and Kit^lo^ enriched) compared to cluster C6 (q-HSC and Kit^hi^ enriched) (Supplementary Fig. 7E). Next, to determine the differential activity and accessibility of lymphoid-specifying transcription factors (TFs) in the identified Kit^hi^ and Kit^lo^ HSC subsets, we analyzed chromatin accessibility variation within TF motifs using chromVAR and gene accessibility. Compared to Kit^hi^ HSCs, we found increased gene accessibility and enrichment for Zinc finger motifs, specifically *Zbtb1 and Zbtb7a*, in Kit^lo^ DARs, which are implicated in the lymphoid differentiation program^19,64–66^ (Fig. 3F, G). In summary, we found that Kit^lo^ HSCs exhibit an epigenetic program, emphasizing an enrichment of lymphoid-specifying transcription factors, in support of their heightened lymphoid potential.

### ZBTB1 regulates HSC multipotency and drives thymic reconstitution potential

**Fig. 4:**
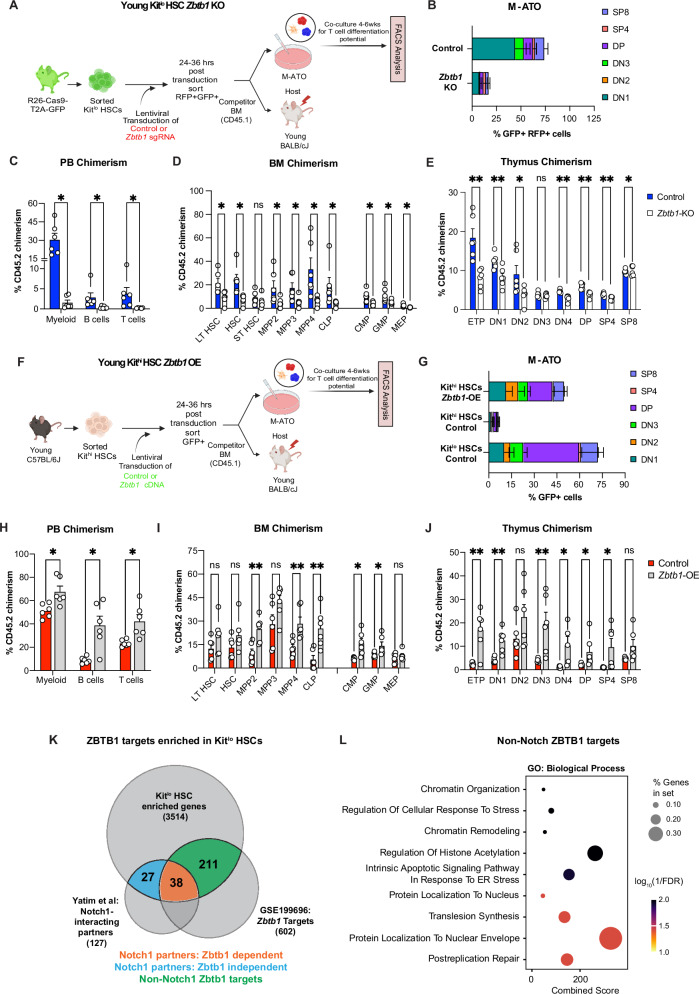
ZBTB1 regulates HSC multipotency and drives thymic reconstitution potential. **A** Experimental schema for T cell differentiation assay and competitive allogeneic HCT with *Zbtb1*-deficient Kit^lo^ HSCs generated with 2-mo young Rosa26Cas9-eGFP KI mice. **B** Frequency of T cell subsets following 6 weeks of culture in M-ATOs. Refer to Supplementary Fig. 13B for gating strategies to define the above populations. Aggregated data across three independent experiments, each performed in duplicates (Control = 3; *Zbtb1*-KO = 3). **C**–**E** Competitive allogeneic HCT with Zbtb1-deficient Kit^lo^ HSCs generated with 2-mo young Rosa26Cas9-eGFP KI mice. Sixteen weeks after competitive HCT, frequency of donor-derived chimerism of mature lineages in the peripheral blood as described in Fig. 2E (**C**), donor-derived chimerism of LSK and MP cell subsets in the BM (**D**), and donor-derived chimerism of T cell precursor thymocyte and mature T cell subsets in the thymus (**E**). All data are from *n* = 6 mice/group, (Control = 6; *Zbtb1*-KO = 6), across two independent experiments. **F** Experimental schema for T cell differentiation assay and competitive allogeneic HCT with *Zbtb1*-OE Kit^hi^ HSCs from young mice. **G** Frequency of T cell subsets following 6 weeks of culture in M-ATO of young Kit^hi^ HSCs. Refer to Supplementary Fig. 13B for gating strategies to define the above populations. Aggregated data across three independent experiments, each performed in duplicates (Control = 3; *Zbtb1*-OE = 3). **H**–**J** Competitive allogeneic HCT with Zbtb1-OE Kit^hi^ HSCs generated with young mice. Sixteen weeks after competitive HCT, frequency of donor-derived chimerism of mature lineages in the peripheral blood as described in Fig. 2E (**H**), donor-derived chimerism of LSK and MP cell subsets in the BM (**I**), and donor-derived chimerism of T cell precursor thymocyte and mature T cell subsets in the thymus (**J**). All data are from *n* = 6 mice/group, (Control = 6; *Zbtb1*-OE = 6), across two independent experiments. Refer to Supplementary Fig. 12 for gating strategies to define the above populations. **K** Previously identified Zbtb1 targets^69^ and Notch1-interacting proteins^71^ enriched in Kit^lo^ HSCs. **L** Pathway enrichment analysis for non-Notch1 Zbtb1 targets enriched in Kit^lo^ HSCs by *gseapy*()^104^ using GO_Biological_Process_2023 libraries. Bubble plot showing representative pathways. Error bars represent mean ± SEM. *P < 0.05, **P < 0.01, ***P < 0.001. *P*-values calculated by nonparametric unpaired two-tailed Mann–Whitney U test. Panels (**A**) and (**F**) were *created in BioRender. Lab, K. (2025)* https://BioRender.com/zy2p156. Source data are provided as a Source Data file, Source Data Fig. 4.

Among members of the ZBTB gene family, *Zbtb1* plays a pivotal role in T cell development^66–68^ and lymphoid reconstitution^66^. However, its role in regulating lymphoid potential in HSCs remains unknown. We hypothesized that Zbtb1 loss impairs the lymphoid potential of Kit^lo^ HSCs. To this end, we used the CRISPR-Cas9 system to generate *Zbtb1*-deficient Kit^lo^ HSCs (Fig. 4A) and confirmed a decrease in ZBTB1 by flow cytometry and Western blot analyses (Supplementary Fig. 8A). *Zbtb1*-deficient Kit^lo^ HSCs showed significantly reduced in vitro T cell potential (Fig. 4B). To evaluate the in vivo reconstituting potential, we competitively transplanted young recipients with control or Zbtb1 knockout (KO) Kit^lo^ HSCs. Sixteen weeks after allo-HCT, we found that Zbtb1-KO recipients exhibited decreased reconstitution across all lineages in the PB and impaired regeneration of the HSPC compartment, including LT HSCs (Fig. 4C, D and Supplementary Fig. 8B). Furthermore, consistent with reduced BM lymphopoiesis in Zbtb1-KO HSC recipients, we observed decreased thymic reconstitution (Fig. 4E and Supplementary Fig. 8C).

Next, we questioned whether reinstating *Zbtb1* expression in megakaryocytic-biased, Kit^hi^ HSCs is sufficient to rescue their lymphoid defect. To test our hypothesis, we overexpressed *Zbtb1* cDNA in Kit^hi^ HSCs (Fig. 4F and Supplementary Fig. 8D) and we observed a rescue of their T cell potential when compared to Kit^hi^ controls, thus partially phenocopying Kit^lo^ HSCs (Fig. 4G) as well as improved lymphoid reconstitution in vivo (Fig. 4H). Comparing competitively transplanted control or *Zbtb1*-overexpressed (OE) Kit^hi^ HSCs, we observed increased trilineage reconstitution, increased chimerism at the level of the Multipotent Progenitor 2 (MPP2), MPP4, and CLPs, thymic reconstitution, and better thymic stromal recovery, in Zbtb1-OE recipients (Fig. 4H–J; Supplementary Fig. 8E–G). Taken together, our data demonstrates that ZBTB1 is required for normal HSPC function, multilineage engraftment, and lineage bias.

Previous studies have identified ZBTB1 targets that facilitate Notch-mediated T cell differentiation in lymphoid progenitors^69^ and support chromatin remodeling following DNA damage^70^. Given *Zbtb1* motif enrichment and enhanced T cell differentiation in Kit^lo^ HSCs, we hypothesized that ZBTB1 targets mediating lymphoid differentiation gene programs are also enriched in Kit^lo^ HSCs. To this end, we queried Kit^lo^ enriched genes for Notch1-interacting proteins^71^. We observed ~50% overlap of Notch1-partners enriched in Kit^lo^ HSCs, of which 60% were ZBTB1 targets identified in a previous CHIP-seq study^69^ (Fig. 4K). Furthermore, we identified an additional subset of ZBTB1 targets (33%) that were not associated with the Notch1 interactome (Supplementary Data 3). To further query for non-Notch-related biological pathways, we performed pathway analysis and identified a strong enrichment for gene sets related to chromatin remodeling and RNA stability (Fig. 4L, Supplementary Data 4).

This data demonstrates that ZBTB1 contributes to an epigenetic regulatory program that facilitates T cell differentiation in primitive Kit^lo^ HSCs through instructive and permissive mechanisms.

### Old Kit^lo^ HSCs exhibit enhanced T cell potential

**Fig. 5:**
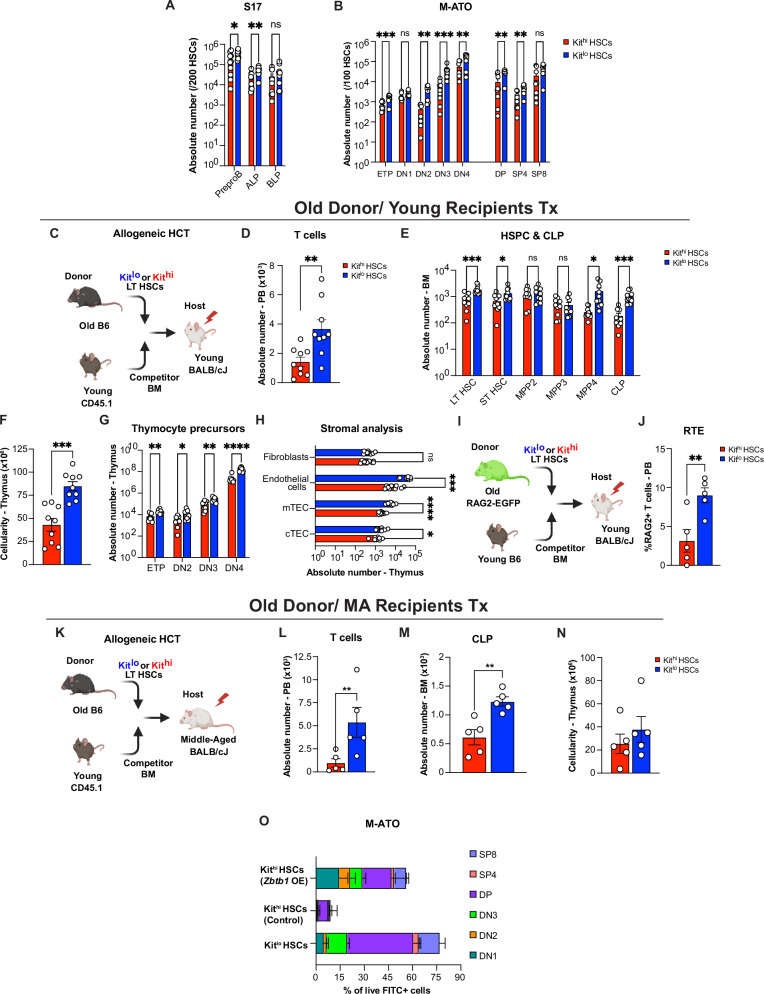
Old Kit^lo^ HSCs exhibit enhanced T cell potential. **A**, **B** Evaluation of in vitro lymphoid progenitor and T cell differentiation potential of Kit^hi^ (red) and Kit^lo^ HSCs (blue) from or 24-mo (old) C57BL/6 mice using the S17 lymphoid assay and murine artificial thymic organoid (M-ATO), respectively, as shown in Fig. 2A. Enumeration of absolute number of lymphoid progenitor cells following 14 days in S17 lymphoid assay (**A**) and absolute number of T cell subsets following 8 weeks of culture in M-ATOs (**B**). Refer to Supplementary Fig. 13A, B for gating strategies to define the above populations. Aggregated data across 3 independent experiments, each performed in triplicate (Kit^lo^ = 3; Kit^hi^ = 3). **C** Experimental schema for competitive allogeneic HCT (allo-HCT) using Kit^hi^ (red) and Kit^lo^ (blue) HSCs from 22–24-mo (old) C57BL/6 mice with competitor bone marrow (BM) cells from B6.SJL-PtprcaPepcb/BoyJ mice transplanted into lethally irradiated young BALB/cJ recipients. **D**–**H** Eight weeks after competitive HCT, enumeration of absolute number of donor-derived T cells in the PB (Kit^hi^ = 9; Kit^lo^ = 9) (**D**), LSK cell subsets and CLP cells (Kit^hi^ = 10; Kit^lo^ = 10) (**E**), total thymic cellularity (Kit^hi^ = 9; Kit^lo^ = 9). **F** Donor-derived T cell precursor thymocyte subsets (Kit^hi^ = 9; Kit^lo^ = 9) (**G**), stromal subsets within thymic CD45-compartment (Kit^hi^ = 9; Kit^lo^ = 9) (**H**). Aggregated data across two independent experiments. **I** Experimental schema to evaluate thymic function following competitive allo-HCT using old HSCs. **J** Frequency of donor-derived Recent Thymic Emigrants (RTE: CD3^+^GFP^+^) in the PB at 8 weeks post-HCT. Refer to Supplementary Fig. 13C for the gating strategy to define the above population. All data are from *n* = 5 mice/group (Kit^lo^ = 5; Kit^hi^ = 5). **K** Experimental schema for competitive allogeneic HCT (allo-HCT) using Kit^hi^ (red) and Kit^lo^ HSCs (blue) from 22–24-mo (old) C57BL/6 mice with competitor bone marrow (BM) cells from B6.SJL-PtprcaPepcb/BoyJ mice transplanted into lethally irradiated middle-aged BALB/cJ recipients. **L**–**N** Eight weeks after competitive HCT, enumeration of the absolute number of donor-derived T cells in PB (**L**), donor-derived CLPs in the BM as defined in Fig. 3D (**M**), and thymic cellularity (**N**). All data are from *n* = 5 mice/group (Kit^lo^ = 5; Kit^hi^ = 5). Refer to Supplementary Fig. 12 for gating strategies to define the above populations. **O** Frequency of T cell subsets following 6 weeks of culture in M-ATO of old Kit^hi^ HSCs following *Zbtb1* OE. Refer to Supplementary Fig. 13B for gating strategies to define the above populations. Aggregated data across three independent experiments, each performed in duplicates (Control = 3; *Zbtb1*-OE = 3). Error bars represent mean ± SEM. *P < 0.05, **P < 0.01, ***P < 0.001, ****P < 0.0001. *P*-values calculated by a nonparametric unpaired two-tailed Mann–Whitney U test. Panels (**C**), (**I**), and (**K**) were *created in BioRender. Lab, K. (2025)* https://BioRender.com/oeh4i7x. Source data are provided as a Source Data file, Source Data Fig. 5.

To determine whether Kit^lo^ HSCs retain their lymphoid potential with age, we evaluated the lymphoid potential of old Kit subsets. Remarkably, even in the case of old mice, we noted a substantial increase in lymphoid progenitor and ATO output from old Kit^lo^ HSCs compared to Kit^hi^ HSCs (Fig. 5A, B), although modest when compared to the young HSCs. This finding is consistent with prior reports of proliferative defects in aged T cell precursors contributing to decreased thymopoiesis^72–74^.

Next, to test their in vivo potential, we competitively transplanted either Kit^lo^ or Kit^hi^ HSCs from old C57BL/6 (CD45.2) mice with rescue BM (CD45.1) cells into lethally irradiated young BALB/cJ recipients (Fig. 5C). At eight weeks post-transplantation, we noted significantly higher numbers of T cells in the peripheral blood of Kit^lo^ vs Kit^hi^ recipients. (Fig. 5D and Supplementary Fig. 9A). Consistent with our experiments using HSCs from young donors, we observed in the BM of Kit^lo^ vs Kit^hi^ recipients significantly higher numbers of LT HSCs, LMPPs and CLPs (Fig. 5E) with comparable myeloid potential (Fig. 5E and Supplementary Fig. 9B). Furthermore, mirroring our findings in HSCs from young mice, we observed in recipients of old Kit^lo^ HSCs enhanced overall thymic cellularity, thymocyte reconstitution, and recovery of TECs and endothelial cells (Fig. 5F–H and Supplementary Fig. 9C), higher RTE output (Fig. 5I, J), and increased CD4+ and CD8+ T cells in the spleen (Supplementary Fig. 9C). At 20-weeks post-HCT, we observed similar trends of enhanced reconstitution in old Kit^lo^ HSC recipients, confirming their long-term reconstituting potential (Supplementary Fig. 9F-M). Next, to examine whether old HSC subsets with preserved lymphoid could augment aged thymic recovery, we competitively transplanted middle-aged recipients with old Kit^lo^ or Kit^hi^ donors (Fig. 5K). We observed increased peripheral T cells (Fig. 5L and Supplementary Fig. 9E) and CLPs in the BM (Fig. 5M), but a modest, non-significant increase in thymic cellularity (Fig. 5N) in Kit^lo^ recipients. Like our *Zbtb1* functional studies with young Kit^hi^ HSCs, we observed a rescue in T cell potential in *Zbtb1*-overexpressed old Kit^hi^ HSCs (Fig. 5O).

Finally, to directly compare the per cell functionality of Kit^lo^ HSCs in the context of aging, we generated mixed chimeras combining Kit^lo^ HSC cells from both young and old mice in an equal competition transplant (Supplementary Fig. 10A). Consistent with our in vitro findings (Fig. 5A, B), we observed an overall decrease in lymphoid reconstitution within the PB, BM, and thymus with old Kit^lo^ HSCs compared to their young counterparts (Supplementary Fig. 10B–E). Pathway analysis revealed decreased enrichment of chromatin remodeling-related gene sets (Supplementary Fig. S10F, Supplementary Data 2 and 5). Additionally, we noted decreased accessibility and expression of *Zbtb1 (*Supplementary Fig. 10G–I*)* in Old Kit^lo^ HSCs, indicating an impaired epigenetic program for lymphoid differentiation. Remarkably, ectopic *Zbtb1* expression in old Kit^lo^ HSCs significantly increased HSC and lymphoid-biased HSPCs, including CLPs, and enhanced peripheral T cell reconstitution (Supplementary Fig. 10J–L).

These findings demonstrate that old Kit^lo^ HSCs exhibit preserved lymphoid potential and contribute to thymic recovery, albeit with reduced output. Additionally, forced ZBTB1 expression in old HSCs can improve HSC and lymphoid-biased engraftment potential.

### KIT^lo^ human BM HSCs exhibit enhanced lymphoid potential

**Fig. 6:**
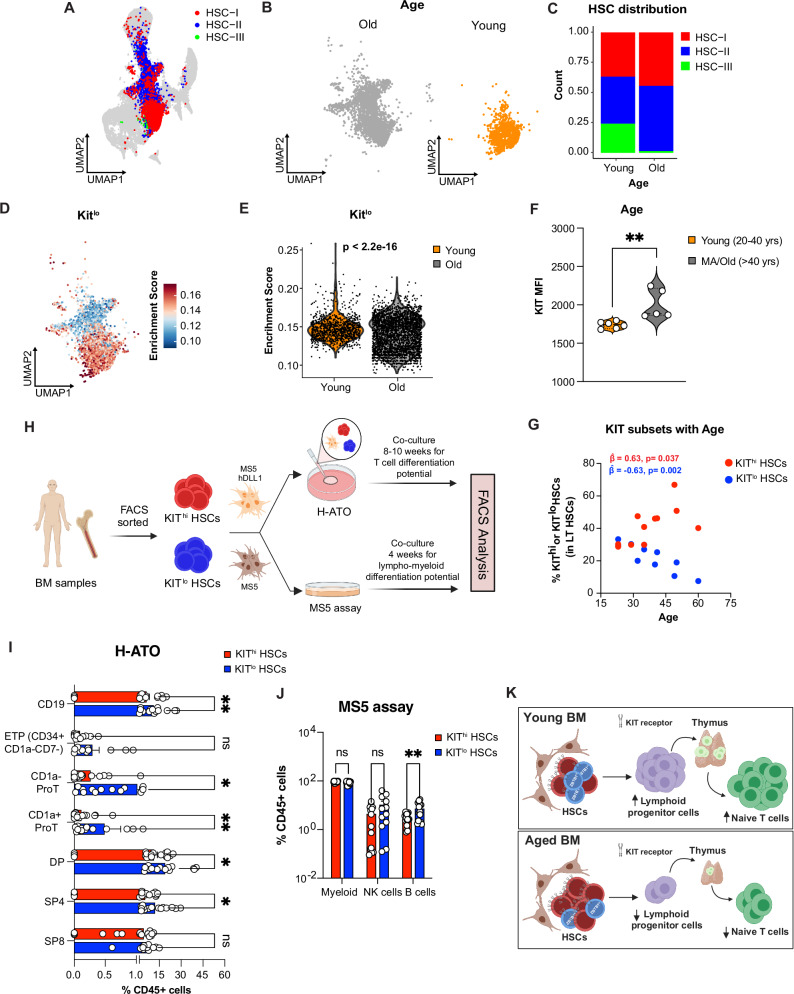
KIT^lo^ human BM HSCs exhibit enhanced lymphoid potential. **A**–**E** Young and old human BM CITE-seq dataset generated and published by Sommarin et al.^76^ **A**–**C** UMAP of CD34+ young and old human BM annotated with HSC subsets (**A**), and age (**B**, **C**) HSC cluster composition by age. **D** Kit^lo^ gene signature (top 50 marker genes from 239 mouse genes identified with human orthologues) was overlaid on human HSC UMAP. **E** Violin plot for Kit^lo^ gene score by age. Statistical analysis was performed using the Wilcoxon test. **F**, **G** FACS analysis of human BM samples. **F** Violin plot of KIT protein expression on phenotypic HSCs (p-HSC: CD34 + CD38-CD10-CD45RA-CD90 + ) by age (young: 20–40 yrs; MA/old: >40 yrs). **G** Scatterplot showing distribution of KIT^hi^ and KIT^lo^ HSC subsets within p-HSCs in human BM samples with varying age. The two-tailed *P*-values generated for (**G**) were based on a single multivariable generalized estimating equation (GEE) logistic model. The model was adjusted for age, KIT^lo^ and KIT^hi^ groups, and their interaction, with an exchangeable working correlation structure to account for the matched data structure. **H** Experimental schema to evaluate in vitro T cell and lympho-myeloid potential of human BM HSC subsets based on differential KIT expression using human artificial thymic organoids (H-ATO) and MS5 assay, respectively. **I** Following 8–10-weeks of culture, enumeration of CD19+ B cells and T cell subsets within CD34+ T cell precursors (Early Thymic Progenitors, ETP: CD34^+^CD1a^−^CD7^−^; CD1a^neg^- ProT: CD34^+^CD1a^−^CD7^+^; CD1a^pos^-ProT: CD34^+^CD1a^+^CD7^+^), and mature T cells (DP: CD34^−^CD5^+^CD7^+^CD4^+^CD8^+^; SP4: CD34^−^CD5^+^CD7^+^CD4^+^CD8^−^; SP8: CD34^−^CD5^+^CD7^+^CD4^−^CD8^+^). Refer to Supplementary Fig. 10E for gating strategies to define the above populations. Aggregated data from 8 independent BM donors, each performed in duplicates, across two independent experiments (KIT^lo^ = 8; KIT^hi^ = 8). **J** Following 4 weeks of culture, enumeration of Lineage output (Neutrophil-Granulocytes: hCD45+CD15+; monocyte-macrophages: hCD45+CD14+; B cells: hCD45+CD19+; NK cells: hCD45+CD56+) after culturing 200 KIT^lo^ and KIT^hi^ HSCs.^79^Aggregated data from 5 independent BM donors, each performed in triplicate or triplicates, across two independent experiments (KIT^lo^ = 5; KIT^hi^ = 5). Refer to Supplementary Fig. 10F for gating strategies to define the above populations. **K** Our model illustrates that hematopoietic stem cells (HSCs) with distinct lymphoid-primed chromatin states and differential ZBTB1 activity, which determines their lymphoid potential, can be distinguished by their CD117/KIT expression. In young bone marrow, Kit^lo^ HSCs (blue) with high ZBTB1 activity exhibit enhanced lymphoid potential, thymic recovery, and T-cell reconstitution, in contrast to Kit^hi^ HSCs (red) with reduced lymphoid potential. The age-related decline in lymphoid potential results from both a proportional shift toward Kit^hi^ HSCs and a global decrease in ZBTB1 activity across all HSC populations. Error bars represent mean ± SEM. *P < 0.05, **P < 0.01, ***P < 0.001, ****P < 0.0001. *P*-values calculated either by nonparametric paired two-tailed Mann–Whitney U test (**I** and **J**), unpaired Mann–Whitney U test (**F**). Panels (**H**) and (**K**) were *created in BioRender. Lab, K. (2025)* https://BioRender.com/fl4hwgn. Source data are provided as a Source Data file, Source Data Fig. 6.

Informed by a recent study that mapped the molecular profiles of human thymic seeding progenitors (TSPs) to their transcriptional counterparts within the HSC compartment^75^, we next sought to identify a comparable human HSC subset with lymphoid potential. Interrogating a CITE-Seq (Cellular Indexing of Transcriptomes and Epitopes by Sequencing) dataset^76^ generated from young and old human bone marrow (BM) samples (Fig. 6A–C and Supplementary Fig. 11A), we mapped our mouse Kit^lo^ gene signature onto human HSCs and noted a significant enrichment in young BM (Fig. 6D, E). We observed lower protein expression of KIT (CD117) on young HSCs by using antibody-derived tag (ADT) reads (Supplementary Fig. 11B). To validate our in-silico findings, we next assessed KIT expression in human BM samples (young: 20–40 years; middle-age/ old: >40 years). In our FACS analysis, we found that phenotypic HSCs (p-HSCs: CD34+CD38-CD10-CD45RA-CD90+) showed the lowest KIT expression (Supplementary Fig. 11C), with higher KIT levels on old p-HSCs (Fig. 6F). Moreover, we noted a statistically significant association between age and the composition of KIT subsets in p-HSCs, indicating an increase in KIT^hi^ and a decrease in KIT^lo^ HSCs, aligning with our observations in mice. (Fig. 6G).

Cord blood (CB) and BM have previously been fractionated based on KIT levels^77,78^. To evaluate the T cell potential of these human BM HSC subsets, we generated artificial human thymic organoids (H-ATO) using purified KIT^hi^ or KIT^lo^ HSCs from human BM samples (Fig. 6H and Supplementary Fig. 11D; Supplementary Data 6). In concordance with our mouse in vitro studies, following 8–10 weeks of culture, we observed significantly higher output of precursor and mature T cells from KIT^lo^ HSCs-derived ATOs compared to KIT^hi^ HSCs (Fig. 6I). To further evaluate the multilineage potential of KIT HSC subsets, we performed a lympho-myeloid differentiation assay, as previously described^79^. While we observed comparable myeloid differentiation potential, Kit^lo^ HSCs exhibited significantly more B cells and a non-significant increase in NK cell differentiation potential (Fig. 6J and Supplementary Fig. 11E). Collectively, these results identify a previously uncharacterized subset of human BM HSCs, KIT^lo^ HSCs – which exhibit enhanced lymphoid potential.

## Discussion

Recent studies have uncovered significant heterogeneity within the HSC compartment and their role in hematopoiesis. This heterogeneity has implications for disease development and treatment. Of note, certain aspects of HSC heterogeneity challenge the established hematopoietic models, with lineage biases and lineage-restricted cells impacting self-renewal dynamics^80,81^. Decisions regarding lympho-myeloid fate are established at multiple levels of the hematopoietic hierarchy: HSCs^22,23,82^, MPP, LMPP, and more mature GMPs and CLPs. At the level of HSCs, the process of epigenetic imprinting confers a distinct advantage for lymphoid priming within multilineage HSCs, as well as maintaining these cell-autonomous features imposed at steady state, even under conditions of stress^19,25^.

Here, we report on the enhanced T cell potential of Kit^lo^ HSCs, observed in both mice and humans. Through combining multiomic single-cell sequencing and functional analyses utilizing a preclinical allo-HCT model, we demonstrate the augmented potential of Kit^lo^ HSCs for T cell lymphopoiesis and to support thymic recovery, independent of age. During the early post-transplant period, we noted a relative increase in DN3 thymocytes relative to other thymocyte precursor subsets. This observation is consistent with previous studies reporting that DN3 thymocytes undergo compensatory expansion to support early post-transplant thymopoiesis, with minimal contribution from T cell precursor thymocytes (ETP and DN2)^11,83,84^. Further investigation into the mechanisms driving these compensatory processes and their implications for immune function is critical to our understanding of post-transplant immune reconstitution.

Previous studies have identified several markers to identify lineage-biased HSCs (lymphoid-biased: CD150^lo^, CD229+, vWF-; myeloid-biased: Neogenin+; megakaryocytic-biased: vWF+). However, these previous studies did not assess the utility of these markers in fractionating aged HSCs or characterizing human HSCs. Our comparative analysis demonstrates that Kit expression provides crucial complementary information to CD150 for identifying functionally distinct HSC populations. While both Kit^lo^ and CD150^lo^ HSCs support lymphoid reconstitution, Kit^lo^ HSCs exhibit superior thymic reconstitution capacity. Notably, CD150^lo^Kit^lo^ HSCs showed enhanced T cell and thymic repopulating potential compared to CD150^lo^ HSCs alone, indicating that Kit expression provides additional resolution for identifying HSCs with robust T cell potential. Further, CD150^hi^Kit^lo^ HSCs exhibited enhanced thymic reconstitution compared to CD150^hi^ HSCs, confirming that Kit expression identifies functionally distinct HSCs even within conventionally defined subsets. Although CD150^hi^ HSCs repopulate thymocyte precursors, they exhibit a T cell differentiation block beyond the DN2 stage, suggesting their myeloid-biased state may impact terminal thymocyte differentiation. The functional differences stem from distinct developmental trajectories: Kit^lo^ and CD150^lo^ HSCs follow classical CLP-dependent differentiation pathways, while CD150^hi^Kit^lo^ HSCs achieve superior thymic reconstitution via a CLP-independent T cell differentiation route. This developmental heterogeneity reinforces the value of KIT as a marker for identifying HSC subsets with diverse yet efficient T cell reconstitution strategies—findings with potential clinical relevance. We further show that Kit is a reliable marker to prospectively identify multilineage or lymphoid-primed Kit^lo^ HSCs in aged BM. Our findings extend to human biology and identify an analogous KIT^lo^ HSC subset with enhanced lymphoid potential in adult BM, suggesting translational relevance in human hematopoiesis, although further functional characterization is warranted to definitively establish their clinical significance. Cumulatively, our studies show that low-expressing Kit HSCs more robustly identify lymphoid-primed HSCs compared to other immunophenotypic-based definitions. This marker system is conserved across species, thus providing a simple fractionating strategy to isolate lineage-biased HSC populations.

Mechanistically, Kit^lo^ HSCs exhibit differential expression and activity of lymphoid-specifying transcription factors (TFs), including *Zbtb1*, indicating their epigenetic program towards a lymphoid fate. We speculate this relationship between Kit signaling and ZBTB1-mediated HSC function operates at multiple levels: transcriptionally, via direct regulation of the *Zbtb1* promoter by KIT signaling effectors; post-transcriptionally, through RNA-binding proteins that stabilize *Zbtb1* mRNA; and post-translationally, via E3 ligases that modulate protein stability.

Studies in mouse models have shown that disruptions in Zbtb1 (Zbtb1-mutant^66^ and Zbtb1-KO^67^) result in T cell developmental defects and severe combined immunodeficiency phenotypes, with no differences within the stem and progenitor compartment, in native hematopoiesis^66,67^. While competitive transplantation studies with Zbtb1-mutants demonstrated additional defects in B and NK cell reconstitution with loss-of-function Zbtb1-mutants^66^, similar transplantation studies were not performed with Zbtb1-KO mice^67^, which did not rule out the possibility of additional reconstitution defects in the complete absence of ZBTB1.

Indeed, we identify ZBTB1 as an essential transcription factor governing hematopoietic stem and progenitor engraftment and lymphoid specification in phenotypic HSCs. Our in vivo experiments reveal that ZBTB1 manipulation produces lineage-specific outcomes. Progenitor analyses showed that ZBTB1 alteration disproportionately affects lymphoid (LMPP and CLP) versus myeloid lineages (CMP, GMP, and MEP) (8-fold versus 2.5-fold decrease with ZBTB1 knockout; 5-fold versus 2-fold increase with ZBTB1 overexpression). These effects were not proportional to the observed differences within the HSC compartment (~2.5–3-fold decrease in ZBTB1-KO; ~1.5-fold increase with ZBTB1 overexpression). This was further reflected in overall differences in cellularity, with Zbtb1-KO resulting in a 65% reduction in thymic cellularity compared to only a 10% decrease in bone marrow cellularity, while Zbtb1 overexpression increased thymic cellularity by 45% versus a 20% increase in bone marrow.

We observed an age-related functional decline in Kit^lo^ HSCs during aging, characterized by competitive disadvantage in old Kit^lo^ HSCs and reduced *Zbtb1* expression. This aging phenotype was further marked by increased expression of the multipotency repressor *Ezh1*^85^. as well as age-associated markers *Selp*^29^ and *Neo1*^86^ in old Kit^lo^ HSCs, which previous studies have linked to HSC functional impairment. Importantly, ZBTB1’s lymphoid-specifying role is further highlighted in our aging experiments, where *Zbtb1* overexpression in aged Kit^lo^ HSCs enhanced lymphoid reconstitution 3.5-fold while myeloid chimerism remained statistically unchanged, demonstrating a clear lineage-specific effect.

Supporting these in vivo findings, our artificial thymic organoid assays—a cell-autonomous readout of T cell differentiation potential—demonstrate that *Zbtb1* overexpression specifically rescues the lymphoid defects in both young and aged megakaryocytic-biased Kit^hi^ HSCs, increasing T cell output by 5-fold. Consistent with prior research, our study suggests ZBTB1 modulates Notch-dependent pathways^69^ – critical for HSPC function and lymphoid fate decisions. Additionally, our in vivo experiment with aged HSCs uncovers a previously unrecognized role for ZBTB1 in influencing broader HSC repopulation capacity across multiple progenitor populations. This function likely regulates cellular survival programs^32,87^, thereby promoting overall HSPC fitness.

Collectively, our findings demonstrate a dual role of ZBTB1 in enhancing HSPC function and lymphoid specification in HSCs. Further mechanistic studies are needed to fully delineate which ZBTB1-specific dependencies mediate its HSC effects in the context of self-renewal versus lineage specification. Examining the direct role at the HSC stage, or if its expression could be indicative of epigenetic states that then facilitate ZBTB1 function at later developmental stages. Future work will also address whether ZBTB1 could be used to augment the generation of mature lymphoid lineages from human BM precursors, with potential therapeutic implications for addressing age-related immune decline.

Long-term reconstituting CD34+KIT^lo^ adult human BM cells have been previously reported^78^. In contrast, fractionating phenotypic HSCs in Cord Blood (CB) based on KIT levels showed reduced reconstitution of KIT^int^ HSCs compared to KIT^hi^ HSCs^77^. These contrasting findings between adult bone marrow and cord blood suggest that the functional importance of KIT signaling varies depending on developmental stage. In our study, combining transcriptional, immunophenotypic, and functional analyses of human BM samples, we identified a KIT^lo^ subset within immunophenotypically defined HSCs with enhanced lymphoid potential, highlighting its translational relevance in human biology. Further functional characterization is warranted to definitively establish the clinical significance of these observations. This understanding is crucial as prospective HSC isolation with subsequent ex vivo expansion^88,89^, holds promise for enhancing immune regeneration after bone marrow transplantation, effectively counteracting treatment-related immunosuppression, and age-associated thymic decline.

## Methods

### Animal use

Young female mice (6–10 weeks of age): C57BL/6J (CD45.2/H-2Kb, JAX#000664), B6.SJL-PtprcaPepcb/BoyJ (CD45.1, JAX# 002014), and B6(C)-Gt (ROSA)26Sor^em1.1(CAG^^−^^cas9*^^−^^EGFP) Rsky/J^ (Rosa26^Cas9^ KI, JAX#028555) mice were purchased from The Jackson Laboratory (JAX). CD45.1 (Stem)-CD45.2 chimeric female mice were obtained from Dr. Joseph Sun. Aged C57BL/6 (CD45.2/H-2Kb) mice (23–24 months of age) were obtained from the National Institute on Aging (Baltimore, MD). Aged female mice (ranging between 18 and 24 months of age) correlate with humans ranging between 56 and 69 years of age. For recipients, we either used young (6–10 weeks of age) BALB/cJ (H-2Kd, JAX#000651) or generated middle-aged BALB/cJ mice (ranging between 14 and 16 months of age) by initially purchasing young BALB/cJ (H-2Kd) mice from JAX and subsequently allowing them to age under controlled conditions in our facility. Middle-aged mice (ranging between 14 and 16 months of age) correlate with humans ranging between 40 and 60 years of age. For consistency across all our transplantation experiments, only female recipient mice were used. RAG2-EGFP-CD45.1 chimeric female mice were generated by crossing FVB-Tg (RAG2-EGFP)1Mnz/J (JAX# 005688) and B6.SJL-PtprcaPepcb/BoyJ (CD45.1, JAX# 002014). Young TCR-OT1 (C57BL/6-Tg (TcraTcrb)1100Mjb/J, JAX#003831) transgenic female mice were obtained from Dr. Andrea Schietinger. All mice were kept under barrier, specific pathogen-free facility (cohousing of 3–5 mice per cage, chow and water ad libitum, 12 h-light cycle [6:00 pm: off]) and were allowed to acclimatize in our vivarium for at least 10-14 days before experiments. All the animal experiments were approved, and mice were euthanized with CO2 gas inhalation under an MSKCC Institutional Animal Care and Use Committee-approved protocol (IACUC).

### Cell lines

To provide a microenvironment that supports lymphoid progenitors in vitro, we cocultured purified HSCs from young and old B6 bone marrow with the S17 stromal cells^48^, which were originally obtained from K. Dorshkind (UCLA). To support in vitro T cell differentiation, we cocultured purified HSCs from young and old B6 bone marrow and human BM with MS5-mDLL4 or MS5-hDLL4 stromal cells^50^, which were obtained from Dr. Gay Crooks (UCLA). Subsequently, both cell lines were maintained in our laboratory as described in the original references. Details of the differentiation cultures are given below under HSC Lymphoid Differentiation Assays.

### Cell preparation and staining

Femurs, tibias, pelvises, and the spine were dissected from euthanized mice and cleaned of muscles and connective tissue on ice in 1× PBS. Bone marrow cells were collected by crushing all the bones with a sterile mortar and pestle in FACS buffer: 1× PBS containing 2% Fetal Bovine Serum (FBS, Hyclone #SH30088.03) and 2 mM EDTA (Invitrogen #15575020). BM cells were enriched for cKit+ cells using mouse CD117 magnetic microbeads (Miltenyi Biotec) per the manufacturer’s protocol.

### Bone marrow analysis and flow cytometric isolation of hematopoietic stem cells

BM cells were stained with fluorochrome-conjugated antibodies as well as propidium iodide (Molecular Probes) or Zombie Aqua™ Fixable Viability Kit (BioLegend). All monoclonal antibodies were purchased from BioLegend and eBiosciences. The monoclonal antibodies included Phycoerythrin-Cyanine7 (PE-Cy7) antibodies to lineage (Lin) markers (CD3ε, CD4, CD8a, B220, Gr-1, Mac-1, and Ter119), Brilliant Violet 711 (BV711) or Brilliant Violet 605 to Sca-1, APC/Cyanine7 to cKit, FITC antibody to CD34, PE antibody to CD150 (SLAM), Pacific Blue to CD48, APC to CD45.2 and PerCP/Cyanine5.5 to CD45.1. Cells were analyzed or sorted using a FACS LSR II UV or Aria II cell sorter (BD Biosciences). In Fig. 1J, Kit expression thresholds were established using the top and bottom 30% of young bone marrow HSCs (Lineage-Sca-1+cKit+ CD34-CD48-Flt3-CD150+) and applied to old samples for comparison. For all experiments (transplantation and in vitro assays) in which Kit^hi^, Kit^lo^, or Kit HSCs were purified, they were double sorted to ensure >95% purity.

To calculate bone marrow cellularity and frequency of donor-derived hematopoietic precursors, two femurs and two tibias of the primary recipient (BALB/cJ) were flushed into FACS buffer at 8- or 20-weeks post-transplant. Collected cells were then incubated with red blood cell lysis buffer (ACK lysis buffer, Thermo Fisher Scientific #A1049201) for 8 min and then washed twice with PBS/2.5% FBS. Cells were resuspended and then stained in PBS/2.5% fetal calf serum with fluorochrome-conjugated antibodies (BioLegend, BD, eBiosciences) against lineage (Lin) markers (CD3ε, CD4, CD8a, B220, Gr-1, Mac-1, and Ter119) (PE-Cy5), Sca-1 (BV711), cKit (APC-Cyanine7), CD150/ SLAM (BV605), CD135/Flt3 (BV421 or PE-Cy5), CD127/IL7Ra (APC), CD48 (BV510), CD16/32 (AF700), CD34 (FITC), CD45.2 (BUV395), CD45.1 (PE-Cy7 or AF700), H-2Kd (PE), and H-2Kb (BV650 or BV786). For additional thymic adhesion molecule analyses, antibodies against CCR7 (BV421), CCR9 (FITC), and PSGL1 (PE) were used for chimerism studies. Following a 30–45 incubation on ice, cells were washed in PBS/2.5% fetal calf serum. Finally, propidium iodide (Molecular Probes) was added as a viability dye before acquiring the data. Cells were analyzed or sorted using a FACS LSR II UV (BD Biosciences) and analyzed using FlowJo (BD Biosciences). Further details on specific vendors, fluorochromes, catalog numbers, dilutions, and gating can be found in Supplementary Data 7.

### Transplantation experiments

For competitive transplantation assays, double-FACS-sorted Kit^hi^, Kit^lo^ HSC cells from young or old CD45.2 mice were mixed with unfractionated BMMCs from young CD45.1 mice. Cells were transplanted via retro-orbital sinus injections into lethally irradiated young (6–8-week-old) or middle-aged (14–16-month-old) BALB/cJ recipient mice (9 Gy, single dose, using a 79 X-Ray source) under isoflurane anesthesia, within 1 h post-irradiation. For the Kit^lo^ equal competition experiment, 375 double-FACS-sorted Kit^lo^ HSCs cells from both young (CD45.1/CD45.2) and old CD45.2 mice were combined with 5 × 10^5^ unfractionated BMMCs from young CD45.1 mice and transplanted into lethally irradiated 6–8-week-old BALB/cJ recipient mice. For Kit versus CD150 equal competition experiment, 400 double-FACS-sorted HSC cells representing specific subsets from two mouse strains were transplanted into lethally irradiated 6–8-week-old BALB/cJ recipient mice. These subsets included Kit^lo^, CD150^lo^Kit^lo^, and CD150^hi^Kit^lo^ HSC subsets from young (CD45.1/CD45.2), CD150^hi^ and CD150^lo^ HSC subsets from CD45.2 mice. The HSC subsets were combined with 5 × 10^5^ unfractionated BMMCs from young CD45.1 mice for transplantation. For RTE experiments, 750 double-FACS-sorted Kit^hi^, Kit^lo^ HSCs cells from young and old RAG2-GFP (CD45.1) mice were mixed with 5 × 10^5^ unfractionated BMMCs from young CD45.2 mice and transplanted into lethally irradiated 6–8-week-old BALB/cJ recipient mice. OT1 Transplant and Adoptive T cell transfer for functional assessment, 750 Kit^hi^ and Kit^lo^ HSCs were double-FACS-sorted from 8–10-week-old OT1 mice and competitively transplanted with 5 × 10^5^ unfractionated competitor BMMCs from (CD45.1) into lethally irradiated young (6–8-week-old) BALB/cJ (H-2Kd) recipients. Eight weeks post-transplant (BMT), the spleens of primary recipients were brought into single-cell suspension, and 1/10 of splenocytes were adoptively transferred into young (8–10wk) C57BL/6 secondary recipients. 4 h later, mice were infected with 5.000 CFU L. monocytogenes bacteria expressing chicken ovalbumin (LM-OVA) to evaluate functional CD8+ T cell response to primary infection. On day 8 post-infection, the spleens of infected mice were analyzed via flow cytometry. Counting beads were used to calculate absolute cell numbers.

### Peripheral blood analysis

Peripheral blood samples were collected in 50 mM EDTA solution via retro-orbital sinus bleeds. Thereafter, PB was incubated with red blood cell lysis buffer (ACK lysis buffer, Gibco #A10492-01) for 8 min and then washed twice with PBS/2.5% fetal bovine serum. Cells were resuspended and then stained in PBS/2.5% fetal calf serum with antibodies against CD3 (AF700), B220 (PE-Cy7), Gr-1 (APC), Mac-1 (APC), NK1-1 (BV605), CD45.2 (BUV395), CD45.1 (BV421), H-2Kd (PE) and H-2Kb (FITC) for chimerism studies for 30 min followed by wash in in PBS/2.5% FBS. Finally, propidium iodide (Molecular Probes) was added as a viability dye before acquiring the data. Cells were analyzed using a FACS LSR II UV and LSR Fortessa X50 (BD Biosciences) and analyzed using FlowJo. To generate absolute T cell counts, complete blood counts, including differentials, were obtained using a ProCyte Dx Hematology Analyzer (IDEXX).

### Thymi harvests: FACS and ELISA analysis

All steps were performed on ice unless indicated. Primary recipient (BALB/cJ) thymi at 8 or 20 wks post-transplant were excised and enzymatically digested following an adapted protocol^54^. Briefly, thymi were mechanically dissociated into ca. 2 mm pieces. Tissue pieces were incubated with a digestion buffer: RPMI 1640 (Thermo Fisher Scientific #11875093), 10% FCS, 62.5 um/mL liberase thermolysin medium (TM, Sigma Aldrich # 5401119001), 0.4 mg/ml DNase I (Thermo Fisher # EN0521) twice for 30 min at 37 °C. Between incubation steps, supernatant containing dissociated cells was transferred to 50 mL conical tubes equipped with a 100 µm filter.

For thymic immunophenotypic analysis, cells were first incubated with Fc block solution (anti-CD16/CD32 antibody) for 10 min on ice. Solution was discarded and cells were stained in PBS/2.5% fetal calf serum with fluorochrome-conjugated antibodies against lineage (Lin) markers (CD19, CD11b, NK1-1, TCRγδ, Gr-1, Ter119) (Biotin), Streptavidin (PE-TexasRed), CD4 (PE-Cy7), CD8 (BV711), CD25 (BV510), CD44 (AF700), cKit (APC), CD135/Flt3 (BV421), CD45.2 (BUV395), CD45.1 (BV605), H-2Kd (PE) and H-2Kb (FITC) for chimerism studies. To characterize the CD45- compartment, thymic cells were stained with antibodies against UEA-1 (FITC), 6C3 (PE), EPCAM (BV605), PDGFRa (BV421), MHC-II (APC), CD31 (PE-Cy7), CD45 (BUV395) and Ter119 (PE-Cy5.5) for 30 min followed by wash in in PBS/2.5% FBS. Antibodies were purchased from both BioLegend and eBiosciences, except Ulex europaeus agglutinin 1 (UEA-1), conjugated to FITC, was purchased from Vector Laboratories (Burlingame, CA). Finally, 7-AAD (Molecular Probes) was added as a viability dye before acquiring the data. Cytometric analysis was performed on FACS LSR II UV (BD Biosciences) and analyzed with FlowJo (BD Biosciences; v.10.8.2). Further details on specific vendors, fluorochromes, catalog numbers, dilutions, and gating can be found in Supplementary Data 7.

For all ELISA experiments, thymi were suspended by mechanical dissociation. The resultant supernatants were quantified using mouse cytokine (IL22 and RANKL) specific ELISA kits from R&D Systems (IL22: # M2200 and RANKL: # MTR00) and read on a TECAN Spark Multimode Microplate Reader.

### Spleen harvest and analysis

All steps were performed on ice unless indicated. Primary recipient (BALB/cJ) spleens at 8- or 20-weeks post-transplant were excised and crushed, filtered through a 70 µM strainer (pluriSelect), intermittently washed with PBS/2.5% fetal bovine serum, and collected in a 50 ml conical tube. Subsequently, the spleen pellet was incubated in red blood cell lysis buffer (ACK lysis buffer, Thermo Fisher Scientific) for 8 min and then washed twice with PBS/2.5% fetal bovine serum. Prior to staining, an aliquot was set aside for counts with the Nexcelom Cellometer K2. For spleen immunophenotypic analysis, cells were first incubated with Fc block solution (anti-CD16/CD32 antibody) for 10 min on ice. Solution was discarded and stained in PBS/2.5% fetal calf serum with fluorochrome-conjugated antibodies against lineage (Lin) markers (CD19, CD11b, NK1-1, TCRγδ, Gr-1, Ter119) (Biotin), Streptavidin (PE-TexasRed), CD4 (PE-Cy7), CD8 (BV711), CD62L (BV510), CD44 (AF700), CD45.2 (BUV395), CD45.1 (BV605), H-2Kd (PE), and H-2Kb (FITC). for chimerism studies.

### HSC lymphoid differentiation assays

#### S17 progenitor assay

Lymphoid progenitor production from young and old B6 bone marrow was measured by mixing FACS-purified 200 Kit^hi^ and Kit^lo^ HSCs with 50,000 S17 stromal cells^48^ in 1.5 mL of methylcellulose (MC) medium. MC medium was prepared by supplementing a-MEM (Thermo Fisher # 12571063) with 30% heat-inactivated FCS, 1% methylcellulose (STEMCELL Technologies #4444), 5 × 10^−^^5 ^M beta-mercaptoethanol (2ME, Gibco #21985023), 2 mM L-glutamine, 50 µg/mL gentamicin (Gibco #15710064), 100 U/mL streptomycin and 100 µg/mL penicillin (Gibco #15070063), 0.1 mM MEM vitamins (Gibco #11120037), 0.1 mM nonessential amino acids (Gibco # 11140050), 1 mM sodium pyruvate (Gibco # 11360070), 20 ng/mL SCF (stem cell factor, Peprotech # 250-03-10UG), 20 ng/mL Flt3L (Peprotech # 250-31L-10UG), and 50 ng/mL IL-7 (Biosource #217-17-10UG). The mixture was plated in non-tissue-culture-treated 3.5-cm^2^ dishes (Becton Dickinson). Following 12 days of culture, the contents of the plates were harvested, cells were enumerated, and examined for production of lymphoid progenitors by flow cytometry. All cultures were placed at 37 °C, 5% CO_2_ humidified incubators until processing.

#### Mouse ATO assay

Prior to setting up the ATO experiments, the MS5-mDLL4 stromal cell lines were thawed and expanded in culture for at least 7 days. The cell lines and ATOs were set up and maintained as initially described^50^.

For the mouse ATO system, FACS-purified 100 Kit^hi^ and Kit^lo^ HSC cells from young and old mice were combined with 150k MS5-mDLL4 stromal cells to form each murine ATO and seeded on culture inserts (Millipore Sigma #PICM0RG50). The media: DMEM/F-12 (Thermo Fisher Scientific #11320033), 50× B27 (Gibco #17504044), 100× Glutamax (Gibco #35050061), 100× Pen/Strep (Gibco #15070063), and 1000× Ascorbic acid (Sigma Aldrich #1043003) was changed twice a week, with 2ME and cytokines (5 ng/ml murine IL-7, 5 ng/ml Flt3L, and 5 ng/ml SCF) added fresh each time.

#### Human ATO and lympho-myeloid differentiation assay

For the human ATO system, healthy human bone marrow mononuclear cells (Supplementary Data 6) were purchased from BIOIVT (Johnson City, TN). The use of commercially purchased human BM material obtained from BIOIVT was approved under MSKCC Institutional IRB 16-834A(35). The collection of human material obtained from BIOIVT was approved by SERATRIALS, LLC under Protocol No. 2010-017, IRB Tracking No. 2016166. All approved healthy volunteers provided informed written consent through SERATRIALS, LLC- a wholly owned subsidiary of BioIVT that acts as the sponsor of biospecimen collections and conducts human research activities in accordance with regulations surrounding human subject safety and protection, including ethical principles originating from the Declaration of Helsinki and consistent with Good Clinical Practice guidelines. FACS-sorted Kit^hi^ and Kit^lo^ HSCs within phenotypic HSCs (CD34^+^CD38^−^CD10^−^CD45RA^−^CD90^+^) were used.

The sorted cells were then aggregated with MS5-hDLL4 (50–150 HSCs and 150k MS5-hDLL4 cells per ATO) and seeded on culture inserts (Millipore Sigma). The human ATO media: RPMI 1640, 25× B27, 100× Glutamax, 100× Pen/Strep, 1000× Ascorbic Acid was refreshed twice a week, supplemented with 2.5 ng/ml recombinant human IL-7 (Peprotech # 200-07-10UG), 5 ng/ml recombinant human Flt3L (Peprotech # 300-19-10 UG), and 5 ng/ml of human SCF (Peprotech # 300-07-10UG) was used only for the first week of differentiation. For analysis via FACS, the ATO was mechanically disrupted, filtered through a 70 µM strainer (pluriSelect), and counted with the Nexcelom Cellometer K2 prior to staining. Harvest timepoints varied, dependent on the initial primary cell population (M-ATO: 6–8 weeks; H-ATO: 8–10 weeks).

For the lympho-myeloid differentiation assay, MS5 cells (Creative Bioarray, #CSC-C2763) were seeded onto 0.1% gelatin (Sigma Aldrich #93482)-coated 24-well plate at an initiating density of 2 × 10^4^/well in α-MEM medium (Thermo Fisher # 12571063) supplemented with 10% FBS, 1% penicillin-streptomycin (Hyclone), and 1% GlutaMAX (Gibco)^79^. Twenty-four hours after plating of MS5 stroma, 200 cells were added into each well in the presence of 0.1 μM DuP-697 (Cayman Chemicals #70645), 20 ng/ml SCF, 10 ng/ml G-CSF (Peprotech # 300-23-10UG), 10 ng/ml FLT3L, 10 ng/ml IL-2 (Peprotech # 200-02-50UG), and 10 ng/ml IL-15 (Peprotech # 200-15-10UG). Cultures were maintained for 4 weeks with weekly half media changes (with 2× cytokines). Cocultures were transferred onto fresh MS5 stroma every two weeks through a 40 μm filter to remove the stromal cells. All the cells in each well were harvested and analyzed by flow cytometry at week 4. The antibodies used to read the lineage outputs in this assay were anti-human CD45-BV480, anti-human CD14-BUV805, anti-human CD15-BV421, anti-human CD19-FITC, anti-human CD56-BUV496, and anti-human CD235a-PE-Cy7. Also refer to Supplementary Data 7.

#### Lentiviral production

For deletion experiments, scramble and Zbtb1 guide RNAs (sgRNA#1: GTTTTAGAGCTAGAA ATAGCAAGTTAAAATAAGGCTAGTCCGTTATCAACTTGAAAAAGTGGCACCGAGTCGGTGC; sgRNA#2: GTTTTAGAGCTAGAAATAGCAAGTTAAAATAAGGCTAGTCCGTT ATCAACTTGAAAAAGTGGCACCGAGTCGGTGC) were cloned into U6-EFS-mCherry (VectorBuilder # VB230426-1731yvd), while Control and mouse Zbtb1 [NM_178744.3] were individually cloned into CMV-EFS-EGFP (VectorBuilder # VB231204-1559ezh) for overexpression experiments and utilized for lentivirus generation. Lentivirus was generated by cotransfecting the above plasmids containing either sgRNAs or cDNA, pMD2.G (Addgene #12259), and psPAX2 (Addgene#12260) into HEK 293T cells (ATCC# CRL-3216). SFEM Media was replaced 6 h post-transfection, and the virus suspension was harvested 36–48 h post-media change. The supernatant was collected and used for lentiviral transductions.

#### HSC lentiviral transduction

Sorted Kit^lo^ and Kit^hi^ HSCs from young, aged B6, and young Rosa26^Cas9^ KI mice were cultured overnight in SFEM (STEMCELL Technologies#9605) media supplemented with mouse cytokines, 50 ng/ml SCF and 10 ng/ml TPO (Peprotech # 315-14-10UG) in single-wells of a 96-well plate (TC treated). The cells were transduced with lentiviral suspensions by spinfection in retronectin (Takara Bio)-coated plates for 90 min. Post spinfection, cytokine-supplemented fresh media were added to the transduced wells. Twenty-four hours later, cells were either sorted for RFP and GFP positivity (for deletion experiments) or GFP expression (for overexpression experiments) using the BD Aria instrument. KO and OE were confirmed by flow cytometric analysis with antibodies against ZBTB1 (AlexaFluor647, Signalway Antibody #C47476-AF647).

#### Western blot analysis

Equal numbers of transduced HSPCs (20,000–30,000 GFP+RFP+ cells) were solubilized in lysis buffer (Cell Signaling Technologies#9803S) supplemented with 10 mM phenylmethylsulfonyl fluoride (PMSF, Sigma Aldrich#93482). Cell lysates were resolved by 4–15% Tris-Acetate Protein Gel (BioRad #4561095) and transferred to PVDF (Millipore#IPVH00010) membranes by a wet transfer protocol. The membrane was probed with the following antibodies from Cell Signaling Technologies: beta-actin (CST, 1:10,000) and ZBTB1 (Proteintech #26287-1-AP, 1:300). The membranes were incubated with chemiluminescent substrates (ZBTB1: SuperSignal West Femto Maximum Sensitivity substrate, Thermo Fisher Scientific #34096 and Actin: Thermo Scientific or Pico Immobilon Western Developing reagent, Millipore#WBLUC0100) and imaged by Amersham imager. Quantitation of total protein expression was performed by normalizing to the loading control.

#### Single-cell multiome ATAC and gene expression cell preparation

Single-cell multiome ATAC + Gene Expression was performed with the 10x Genomics system using Chromium Next GEM Single Cell Multiome Reagent Kit A (#1000282) and ATAC Kit A (#1000280) following the Chromium Next GEM Single Cell Multiome ATAC + Gene Expression Reagent Kits User Guide and demonstrated protocol - Nuclei Isolation for Single Cell Multiome ATAC + Gene Expression Sequencing. Briefly, cells (viability 95%) were lysed for 4 min and resuspended in Diluted Nuclei Buffer (10x Genomics #PN- 2000207). Lysis efficiency and nuclei concentration were evaluated on the Countess II automatic cell counter by trypan blue staining. Nuclei were loaded per transposition reaction, with a targeting recovery between 1000 and 10,000 nuclei after encapsulation. After the transposition reaction, nuclei were encapsulated and barcoded. Next-generation sequencing libraries were constructed following the User Guide, which were sequenced on an Illumina NovaSeq 6000 system.

### Sequencing data processing

#### Single-cell RNA-seq

##### i) Preprocessing and downstream data analysis

FASTQ files were processed using the 10x Cell Ranger package (v.7.01). The Cell Ranger generated filtered_feature_bc_matrix.h5 files were processed following the guidelines on the shunPykeR GitHub repository (https://github.com/kousaa/shunPykeR), an assembled pipeline of publicly available single-cell analysis packages put in coherent order, that allows for data analysis in a reproducible manner and seamless usage of Python and R code. Genes that were not expressed in any cell and ribosomal, and hemoglobin genes were removed from the downstream analysis. Each cell was then normalized to a total library size of 10,000 reads, and gene counts were log-transformed using the log(X + 1) formula, in which log denotes the natural logarithm. Principal component analysis (components = 20) was applied to reduce noise prior to data clustering. To select the optimal number of principal components to retain for each dataset, the knee point (eigenvalues smaller radius of curvature) was used. Leiden clustering^90^ (resolution = 0.9) was used to identify clusters within the PCA-reduced data.

Quality of the single cells (Supplementary Fig. 14A, B) was computationally assessed based on total counts, number of genes, mitochondrial and ribosomal fraction per cell, with low total counts, low number of genes (≤1000), and high mitochondrial content (≥0.2) as negative indicators of cell quality. Cells characterized by more than one negative indicator were considered “bad” quality cells. Although cells were negatively sorted prior to sequencing for the CD45 marker, a small amount of non-hematopoietic cells (expressing no Ptprc) were detected within our dataset. To remove bad quality cells and contaminants in an unbiased way, we assessed them on a cluster basis rather than individually. Leiden clusters with a “bad” quality profile and/or a high number of contaminating cells were removed. Finally, cells marked as doublets by Scrublet (v.0.2.1)^91^ were also filtered out. Overall, a total of 2325 cells, representing ~8.4% of all our data, were excluded from further analysis (see Fig. S2 for per-sample metrics). After removal of these cells, we calculated highly variable genes (HVG = 3000) and re-performed PCA with unsupervised clustering analysis (components = 20), followed by batch effect correction across all samples using Harmony (v.0.0.1)^92^ to assist annotation of cell-type subsets within the dataset, using sample as the batch key.

Batch effect correction was performed using Harmony with default parameters, using sample identity as the batch key. To assess integration quality, we used the diversity score, specifically the Local Inverse Simpson’s Index (iLISI)^92^. Prior to Harmony integration, analysis of our young and old samples revealed an integration score (iLISI) of 1.07, which indicates poorly integrated samples, and a cell-type identity score (cLISI) of 1.31. After Harmony integration, we observed a high median iLISI score of 2.3 when combining our old and young samples, which is indicative of well-integrated biological replicates. Additionally, we evaluated accuracy by examining the retention of cell-type distinctions post-integration, where the median cell-type LISI (cLISI) was 1.23, reflecting that our integration preserved biological differences among cell types effectively (Supplementary Fig. 14C). To transfer the ‘HSC subset’ annotations from our young reference dataset to the aged HSC dataset, we employed *scanpy*’s ingest function *(sc.tl.ingest())* using default parameters^93^. The integration was performed using pre-computed UMAP embedding coordinates generated from our young dataset as the spatial reference and the ‘HSC_subtype’ observation key for cell-type annotation transfer.

##### ii) Defining Kit^lo^ and Kit^hi^ HSC subsets

Owing to the significant drop-out effect in scRNA-seq data^94^, it is impossible to ascertain if a cell has low/mid (and sometimes high) Kit expression versus no expression. To methodically deal with this, we performed _data denoising and_ imputation using the MAGIC (Markov Affinity-based Graph Imputation of Cells, v3.0.0) method^95^, to denoise and distinguish between dropouts and genuinely low gene expression values. Post-MAGIC imputation, we proceeded to set the thresholds for *Kit*, specifically, we calculated the 20th and 80th percentile expression value across all cells. Subsequently, cells were classified into Kit^lo^ (*Kit* expression below the 20th percentile), Kit^hi^ (*Kit* expression above the 80th percentile), and Kit^mid^ (*Kit* expression between the 20th and 80th percentile). This approach helped to enhance the quality of the data by denoising and imputing the missing values, thereby providing a more accurate representation of the gene expression landscape.

##### iii) Differential expression analysis

Differential expression analysis for comparisons of interest was performed with MAST (Model-based Analysis of Single-cell Transcriptomics) using the likelihood ratio test^96^. In all cases, differentially expressed genes were considered statistically significant if the FDR-adjusted *p*-value was less than 0.05. Gene set score analysis between comparison groups was performed using the *sc.tl.scoregenes()* function from *scanpy*. This function calculates cluster-specific gene signatures by computing average expression scores and subtracting scores from a randomly sampled reference gene set. To ensure robust results, we implemented a detection threshold requiring each differentially expressed gene to be present in at least 10% of cells within each analyzed cluster. The comprehensive results of this analysis, including detailed per-cluster gene expression percentages, can be found in ‘Supplementary Data table 2_DEG analysis’.

##### iv) Differential abundance (DA) analysis

DA HSC subset across age groups was identified by sampling neighborhoods of cells from a k-nearest neighbors (k-NN) graph and looking for enrichment of either age in each neighborhood as implemented in MiloR^97^. MiloR is a graph-based statistical method to compute differential cellular abundances in neighborhoods of cells. The 15 batch-corrected latent dimensions from *scanpy* (v.1.11.2) were used for MiloR (v.3.21) k-NN graph construction (*k*  =  35) and neighborhood indexing (proportion = 0.1). DA testing was performed with generalized linear models, including age as covariates (neighborhoods significant if spatial corrected FDR  <  0.25).

### Single-cell ATAC sequencing

#### Preprocessing, dimensionality reduction, clustering

Single-cell ATAC-seq from young and old mice were aligned to the mm10 genome, and we processed the cellranger output file, fragments.tsv, with ArchR (v.1.0.3)^98^ was used for downstream analysis. We performed QC filtering on scATAC-seq using ArchR with the default parameters of *createArrowFiles*() (Supplementary Fig. 7). We retained cells containing at least 1000 and at most 100,000 fragments. Next, we filtered out the cells that did not pass QC in the corresponding scRNA-seq data, thus retaining 11,493 cells common to both scRNA-seq and scATAC-seq. We then performed dimensionality reduction using iterative latent semantic indexing (LSI) on the top 25,000 variable features from the tile matrix to get a reduced dimensionality of 30 components with *addIterativeLSI*() function in ArchR. To generate visualizations, we employed the *addUMAP*() function in ArchR with the following settings: nNeighbors = 20; minDist = 0.1. For clustering, we utilized the *addClusters*() function in ArchR, specifying the parameters as follows: method = ‘Seurat’, knnAssign = 10, and maxClusters = 10. Next, we used Harmony^92^ to perform batch effect correction and using the sample as the batch key.

#### Peak calling and TF motif accessibility scoring

Each individual sample was pseudo bulked for peak calling with MACS2 (v.2.2.9.1)^99^ peak caller and iterative peak overlapping removal within ArchR (using default settings). We subsequently added motif annotations using *addMotifAnnotations*() with the CisBP motif database and computed chromVAR^100^ deviations for each single cell with *addDeviationsMatrix*(). To identify differentially accessible motifs within each group of interest, we applied the *rank_genes_groups*() function in *scanpy*. With the following settings: method = ‘wilcoxon’ and corr_method = ‘benjamini-hochberg’, and performed the analysis on the *chromVAR* (v.1.30.1) z-score matrix.

#### Processing human single-cell CITE-seq

We analyzed the young human bone marrow CITE-seq from Sommarin et al.^76^ using Seurat (v.5.3.0)^101^. The authors provided the peak matrix aligned to the hg38 genome. We used the sample BM_34 for which the authors provided the cell-type annotations. We processed the scRNA-seq and CITE-seq data for young and old bone marrow using Seurat. The hashtagged cells were demultiplexed using *HTODemux*() from Seurat. The cell-type annotations were provided by the authors for young bone marrow samples labeled yBM1_hpc and yBM2_hpc. We predicted cell-type annotations for the old bone marrow cells by label transfer using *FindTransferAnchors*() and *TransferData*() functions. We obtained the Kit^lo^ gene signature from our mouse scRNA-seq data through differential gene expression analysis. To extend this signature to our human bone marrow HSPC dataset, we used Ensembl^102^ Biomart to identify human orthologues of the mouse genes. Subsequently, we scored the Kit^lo^ gene signature in human scBM HSCs using Seurat’s *AddModuleScore*() function.

### Statistical analysis

Data was processed in GraphPad Prism 10.0 software. Statistical comparisons between 2 groups were performed with either the nonparametric unpaired Mann–Whitney U test, two-way ANOVA, ComBat batch effect correction^103^, or Wilcoxon rank sum test with continuity correction as indicated.

### Reporting summary

Further information on research design is available in the Nature Portfolio Reporting Summary linked to this article.

## Supplementary information

Supplementary Information

Description of Additional Supplementary Files

Supplementary Data 1

Supplementary Data 2

Supplementary Data 3

Supplementary Data 4

Supplementary Data 5

Supplementary Data 6

Supplementary Data 7

Reporting Summary

Transparent Peer Review file

## Source data

Source Data

## Data Availability

All data are included in the Supplementary Information or available from the authors, as are unique reagents used in this Article. The raw numbers for charts and graphs are available in the Source Data file whenever possible. Raw Western blot images (related to Supplementary Fig. 8A) are provided as a source data file. The Multiome single-cell RNA and ATAC data generated in this study have been deposited in the GEO database under accession code GSE246464. Mouse CITE-seq data used in this study are available in the GEO database under accession code GSE243197 (related to Supplementary Fig. 2F, G, Solomon et al.^42^). Human bone marrow CITE-seq data from young and old donors used in this study were downloaded from OSF Archive [https://osf.io/vdf42/] (Related to Fig. 6, Sommarin et al.^76^). The processed multiome data are available at Zenodo (10.5281/zenodo.15521121). The raw numbers for charts and graphs are available in the Source Data file whenever possible. Source data are provided with this paper.

## Code availability

This paper does not report original code or any new algorithms. Code for figure reproducibility is available through https://github.com/HElias01/AgingHSC-Multiome.
