# Supplementary material for: Kitlo hematopoietic stem cells exhibit distinct lymphoid-primed chromatin landscapes that enhance thymic reconstitution
Source: Nat Commun. 2025 Jul 4;16:6170. doi: 10.1038/s41467-025-61125-1 (PMC12227609; doi:10.1038/s41467-025-61125-1)
Supplement: Supplementary file 1 — Supplementary Information [file 41467_2025_61125_MOESM1_ESM.pdf]

Supplemental information for:

Distinct chromatin states enhance multilineage function in HSC subsets that drive improved thymic reconstitution.

Harold K. Elias[1,2†], Michael G. Kharas[1†] and Marcel R.M. van den Brink[12†]

[†]Corresponding author
Correspondence: eliash@mskcc.org(HKE); kharasm@mskcc.org (MGK); mvandenbrink@coh.org (MRMvdB)

I. Supplementary Figures

II. Supplementary Figure Legends

III. Supplementary Data Table Legends

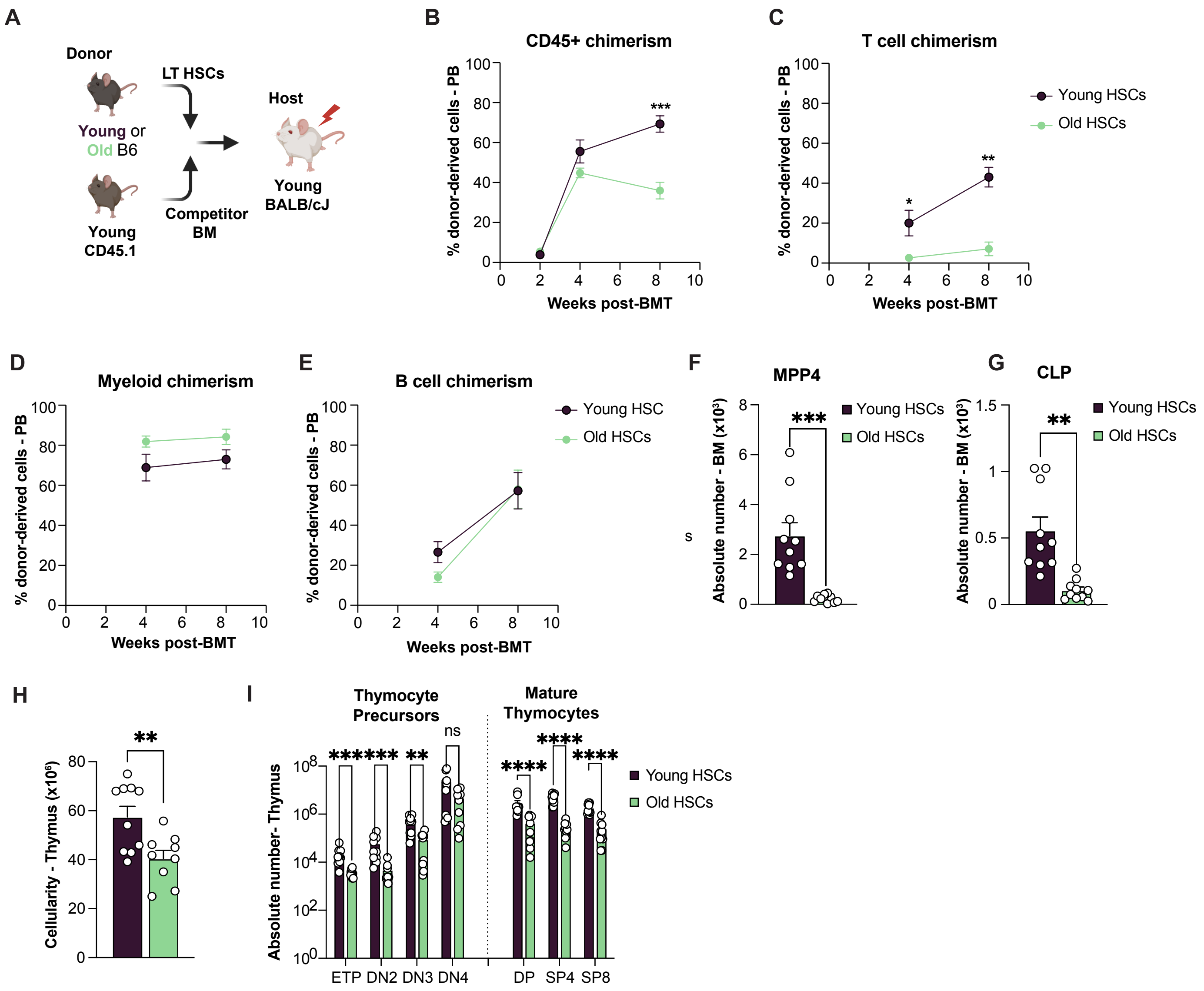
A
Donor
LT HSCs
Young or
Old B6
Host
Young
CD45.1
Competitor
BM
Young
BALB/cJ
B
CD45+ chimerism
% donor-derived cells - PB
Weeks post-BMT
***
C
T cell chimerism
Young HSCs
Old HSCs
*
**
D
Myeloid chimerism
E
B cell chimerism
Young HSC
Old HSCs
F
MPP4
Absolute number - BM (x10³)
***
Young HSCs
Old HSCs
G
CLP
**
H
Cellularity - Thymus (x10⁶)
**
I
Thymocyte
Precursors
Mature
Thymocytes
Absolute number- Thymus
ns
ETP
DN2
DN3
DN4
DP
SP4
SP8

<u>Supplementary Figure Legends</u>

Supplementary Figure 1: Reduced lymphoid output in aged HSCs impairs thymic recovery. **(A)** Experimental schema for competitive allogeneic HCT (allo-HCT) using 2-mo (young) and 24-mo (old) 750 HSCs [Lin^{-}c-Kit^{+}Sca1^{+} (L^{-}S^{+}K^{+}) CD150^{+} CD34^{-}CD48^{-}] from C57BL/6 mice with competitor bone marrow (BM) cells from B6.SJL-PtprcaPepcb/BoyJ mice transplanted into lethally irradiated 7-week-old (young) BALB/cJ recipients. **(B-E)** Frequency of donor-derived chimerism of all hematopoietic cells mature lineages **(B),** T cell **(C),** Myeloid **(D),** B cell **(E)** in the PB at the indicated time points. All data are from n=6-7 mice/group (young=6, old=7). **(F-G)** Eight weeks after competitive HCT enumeration of absolute number of LMPP cells (LMPP/MPP4: Lineage^{-}Sca-1^{+}cKit^{+} Flt3^{+}CD150^{-}) **(F)**, CLP cells (CLP: Lineage^{-}IL7Ra^{+}Flt3^{+}Sca$^{mid/lo}$ Kit^{lo}) **(G)**. **(H-J)** Post-HCT thymi analysis for total thymic cellularity **(H)**, enumeration of absolute number of donor-derived cells for thymocyte precursors (ETP: Lineage^{-} CD4^{-} CD8^{-}CD44^{+} CD25^{-}Kit^{+}; DN2: Lineage^{-} CD4^{-} CD8^{-}CD44^{+} CD25^{+}; DN3: Lineage^{-} CD4^{-} CD8^{-}CD44^{-} CD25^{+}), Mature T cells (DP: Lineage^{-} CD4^{+} CD8^{+}; SP4: Lineage^{-} CD4^{+} CD8^{-}; SP8: Lineage^{-} CD4^{-} CD8^{+}**)** **(I)**. Refer to Supplementary Figure 12 for gating strategies to define above populations. All data are from n=9-10 mice/group (young=10, old=9), across two independent experiments. Error bars represent mean ± SEM. *P<0.05, **P<0.01, ***P<0.001, ****P<0.0001. P values calculated by nonparametric unpaired two-tailed Mann-Whitney U test. Panel **A** was *Created in BioRender. Lab, K. (2025) https://BioRender.com/oeh4i7x.* Source data are provided as a Source Data file, Source Data Supplementary Figure 1.

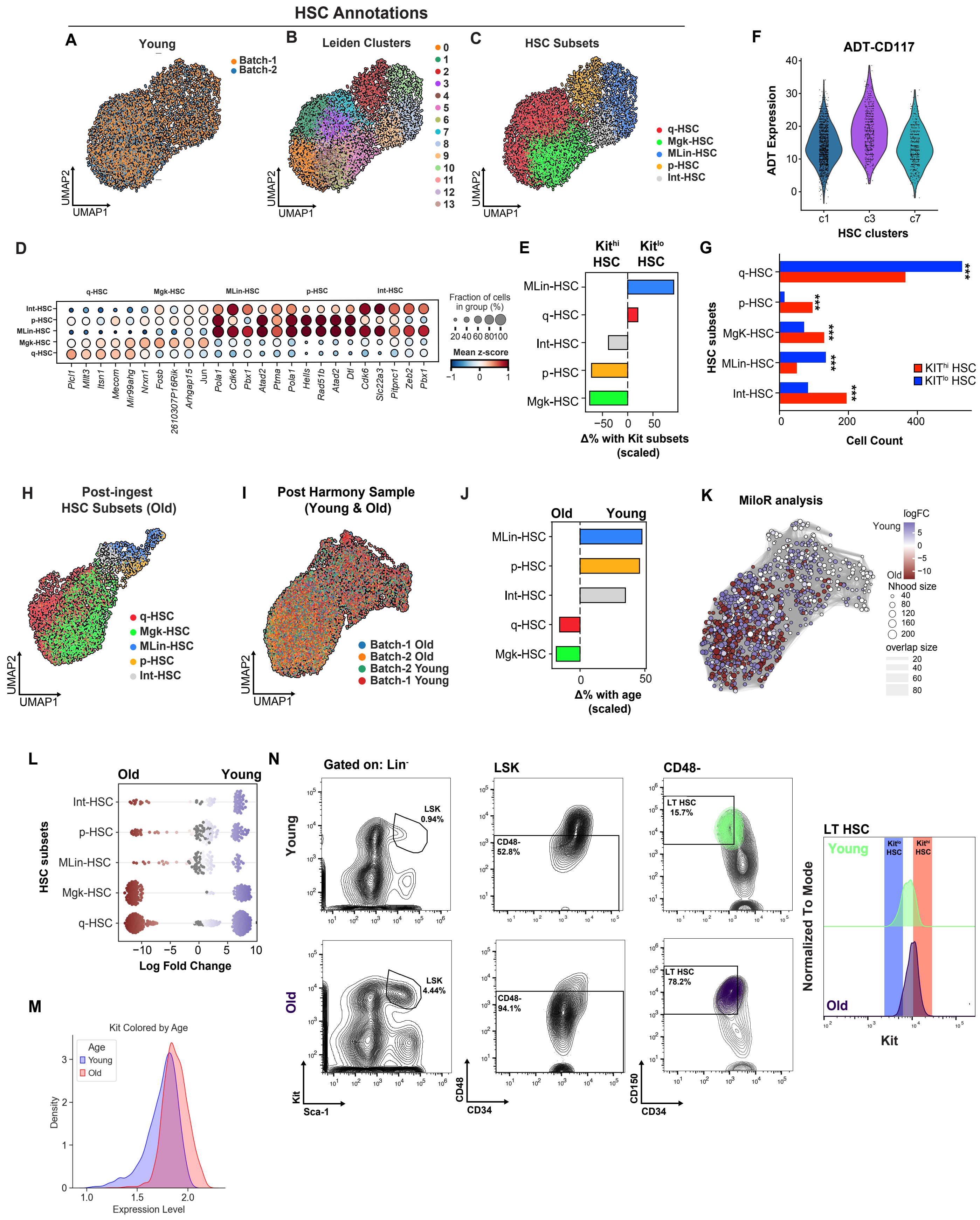
HSC Annotations
A
Young
Batch-1
Batch-2
UMAP2
UMAP1
B
Leiden Clusters
0
1
2
3
4
5
6
7
8
9
10
11
12
13
C
HSC Subsets
q-HSC
Mgk-HSC
MLin-HSC
p-HSC
Int-HSC
F
ADT-CD117
ADT Expression
c1
c3
c7
HSC clusters
D
q-HSC
Mgk-HSC
MLin-HSC
p-HSC
Int-HSC
Plcl1
Mllt3
Itsn1
Mecom
Mir99ahg
Nrxn1
Fosb
2610307P16Rik
Arhgap15
Jun
Pola1
Cdk6
Pbx1
Atad2
Ptma
Pola1
Hells
Rad51b
Atad2
Dll
Cdk6
Slc22a3
Pitpnc1
Zeb2
Pbx1
Fraction of cells in group (%)
20 40 60 80 100
Mean z-score
E
Kit^hi HSC
Kit^lo HSC
Δ% with Kit subsets (scaled)
G
HSC subsets
KIT^hi HSC
KIT^lo HSC
Cell Count
H
Post-ingest HSC Subsets (Old)
I
Post Harmony Sample (Young & Old)
Batch-1 Old
Batch-2 Old
Batch-2 Young
Batch-1 Young
J
Old
Young
Δ% with age (scaled)
K
MiloR analysis
logFC
Young
Old
Nhood size
overlap size
L
Old
Young
HSC subsets
Log Fold Change
M
Kit Colored by Age
Age
Young
Old
Density
Expression Level
N
Gated on: Lin-
LSK
CD48-
Young
Old
LSK 0.94%
LSK 4.44%
CD48- 52.8%
CD48- 94.1%
LT HSC 15.7%
LT HSC 78.2%
Kit
Sca-1
CD48
CD34
CD150
LT HSC
Kit^lo HSC
Kit^hi HSC
Normalized To Mode

Supplementary Figure 2: Single-cell analysis reveals Kit expression defines HSC functional heterogeneity. (A) UMAP of young HSCs after Harmony batch correction annotated by unsupervised Leiden cluster analysis (**B**), manually annotated HSC subsets (q-HSC: Quiescent HSCs; Mgk-HSCs: Platelet-biased HSCs; MLin-HSC: Multilineage HSCs; p-HSC: Proliferative HSCs; Int-HSC: Intermediate HSCs) (**C**). (**D**) Dot plot showing marker genes for each HSC subset. Circle size and color indicate percentage and expression level, respectively. (**E**) Composition of defined HSC subtypes in Kit^{hi} and Kit^{lo} subsets in young HSCs as shown by scaled change in frequency. (**F**) Violin plot of CD117 or Kit ADT-expression on HSC annotated LSK subsets in previously published CITE-seq dataset.[42] (**G**) Composition of annotated HSC subtypes (as defined in **Supplementary Figure 2B**) in KIT^{hi} and KIT^{lo} HSC subsets as shown by cell number within each group. Pearson's Chi-squared statistical analysis was performed to assess significance (***$p<0.001$). (**H**) UMAP of old HSCs annotated by HSC subsets identified by *ingest*() function of *scanpy*,[93] using young HSCs, as reference dataset. **(I)** UMAP of combined young and old HSCs after Harmony batch correction annotated by sample. (**J**) Composition of defined HSC subtypes in old and young HSCs as shown by scaled change in frequency. **(K)** A neighborhood graph of the results from Milo differential abundance testing. Nodes are neighborhoods, colored by their log fold change across ages. Non-differential abundance neighborhoods (FDR 10%) are colored white, and sizes correspond to the number of cells in each neighborhood. Graph edges depict the number of cells shared between neighborhoods. The layout of nodes is determined by the position of the neighborhood index cell in the UMAP in panel **Figure 1D. (L)** Beeswarm plot of the distribution of log fold change across age in neighborhoods containing cells from different cell type clusters. Differential abundance neighborhoods at FDR 35% are colored. HSCs detected as differentially abundant annotated by HSC subsets (as defined in **Figure 1A**). **(M)** Histogram for magic imputed

Kit gene expression by age. **(N)** Representative FACS plots showing the gating strategy to define Kit^{hi} and Kit^{lo} HSC subsets in BM from young and old mice.

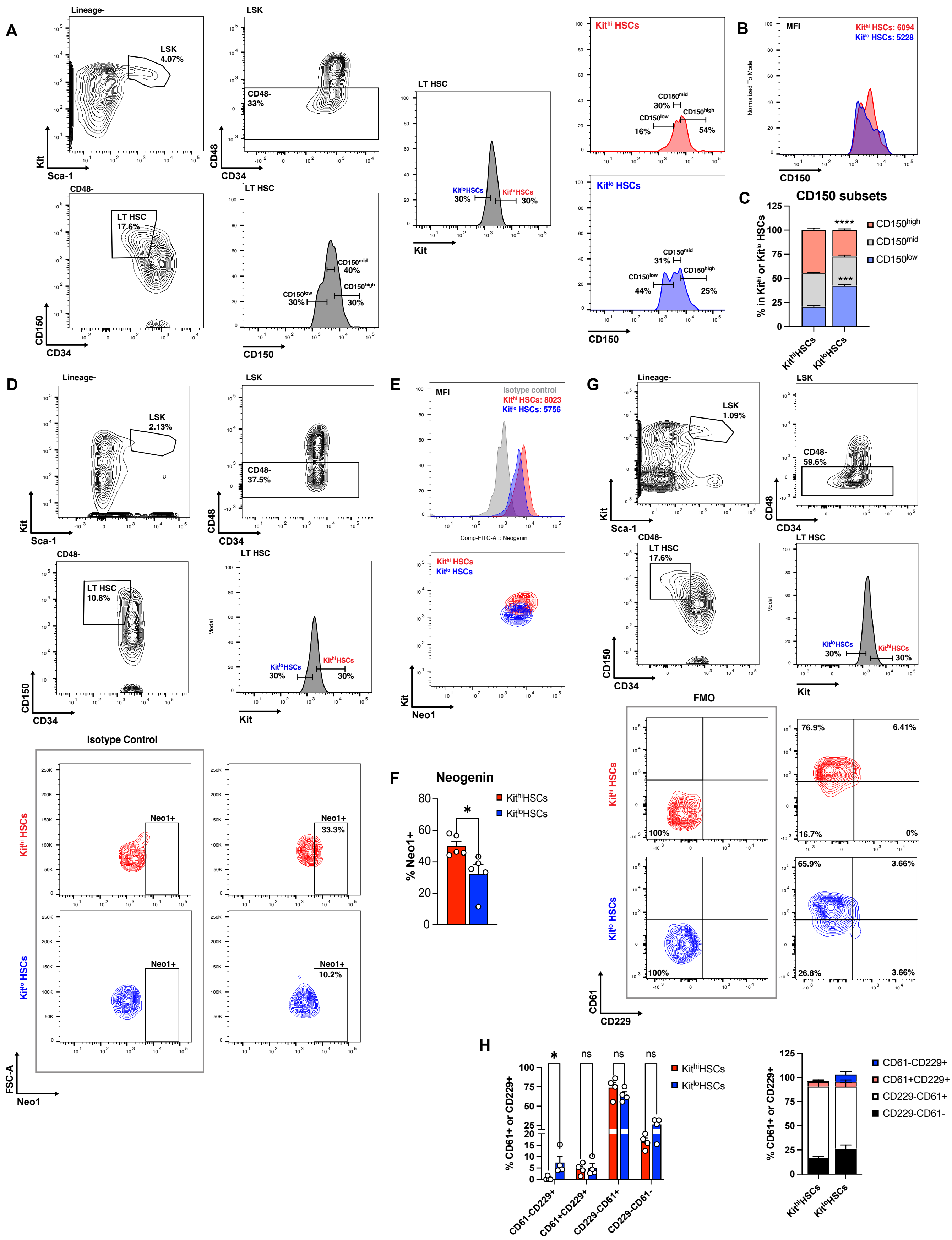
A
Lineage-
LSK
4.07%
Kit
Sca-1
CD48-
33%
CD48
CD34
LT HSC
17.6%
CD150
40%
30%
Kit
Kit^{hi} HSCs
Kit^{lo} HSCs
CD150^{mid}
CD150^{low}
CD150^{high}
16%
54%
31%
44%
25%
B
MFI
Kit^{hi} HSCs: 6094
Kit^{lo} HSCs: 5228
Normalized To Mode
C
CD150 subsets
% in Kit^{hi} or Kit^{lo} HSCs
****
***
Kit^{hi}HSCs
Kit^{lo}HSCs
D
2.13%
37.5%
10.8%
Modal
E
Isotype control
Kit^{hi} HSCs: 8023
Kit^{lo} HSCs: 5756
Comp-FITC-A :: Neogenin
Neo1
F
Neogenin
% Neo1+
*
G
1.09%
59.6%
FMO
76.9%
6.41%
16.7%
0%
100%
65.9%
3.66%
26.8%
CD61
CD229
Isotype Control
Neo1+
33.3%
10.2%
FSC-A
H
% CD61+ or CD229+
ns
CD61-CD229+
CD61+CD229+
CD229-CD61+
CD229-CD61-

Supplementary Figure 3: Phenotypic characterization of Kit^{hi} and Kit^{lo} HSC subsets. Characterization of BM KIT HSC subsets. Representative FACS plots showing the gating strategy to define Kit^{hi} and Kit^{lo} HSC subsets in BM from young based and assess for expression of CD150 (**A-C**), Neogenin (Neo1) (**D-F**), and CD61 and CD229 (**G-H**). All data are from n=4-5 mice/group. Error bars represent mean ± SEM. *P<0.05, **P<0.01, ***P<0.001. P values calculated by nonparametric unpaired two-tailed Mann-Whitney U test. Source data are provided as a Source Data file, Source Data Supplementary Figure 3.

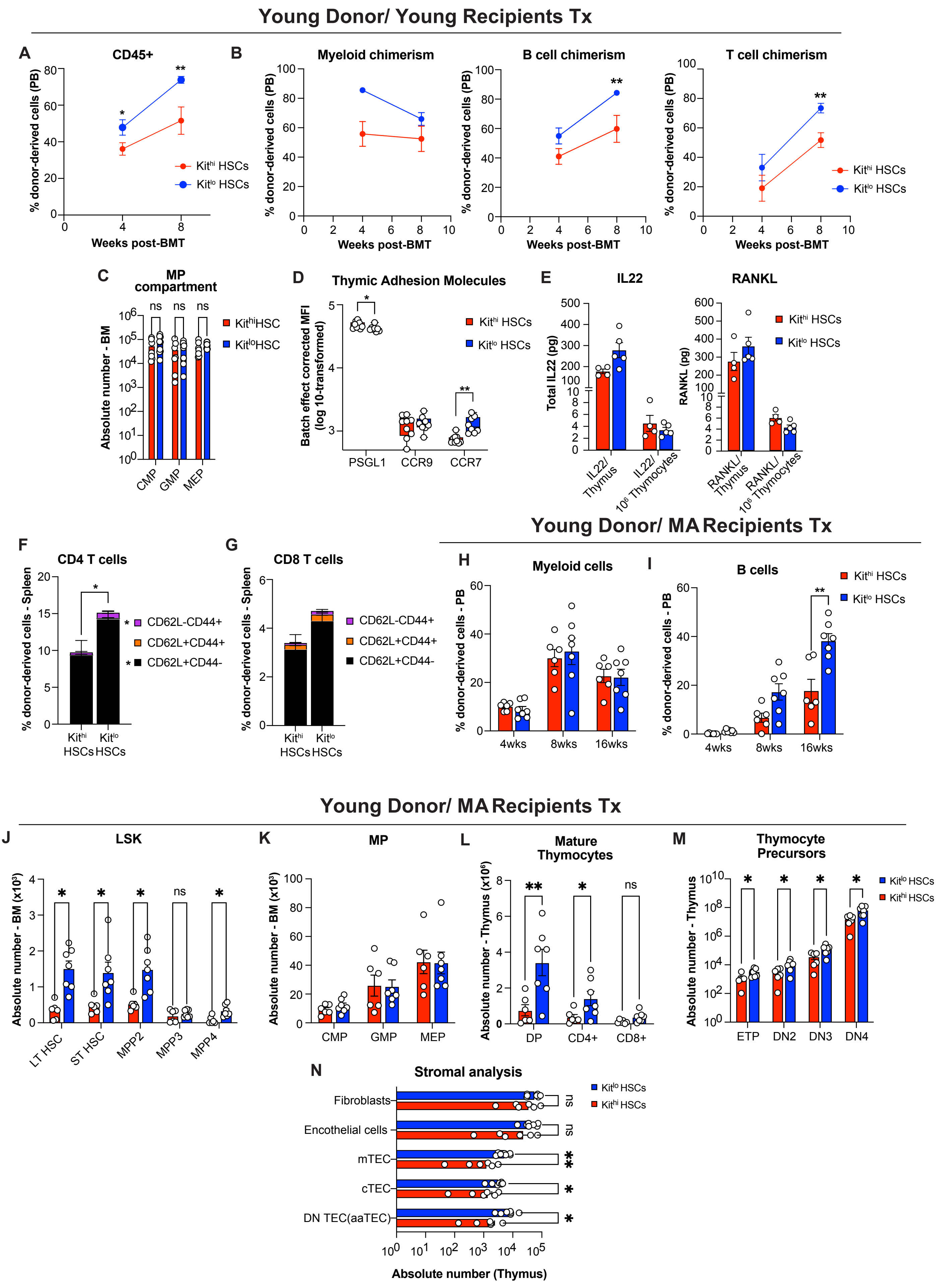

Young Donor/ Young Recipients Tx
A
CD45+
% donor-derived cells (PB)
Weeks post-BMT
Kit^hi HSCs
Kit^lo HSCs
B
Myeloid chimerism
B cell chimerism
T cell chimerism
C
MP compartment
Absolute number - BM
Kit^hi HSC
Kit^lo HSC
CMP
GMP
MEP
D
Thymic Adhesion Molecules
Batch effect corrected MFI (log 10-transformed)
PSGL1
CCR9
CCR7
E
IL22
RANKL
Total IL22 (pg)
RANKL (pg)
IL22/ Thymus
IL22/ 10^6 Thymocytes
RANKL/ Thymus
RANKL/ 10^6 Thymocytes
Young Donor/ MA Recipients Tx
F
CD4 T cells
G
CD8 T cells
% donor-derived cells - Spleen
CD62L-CD44+
CD62L+CD44+
CD62L+CD44-
Kit^hi HSCs
Kit^lo HSCs
H
Myeloid cells
I
B cells
% donor-derived cells - PB
4wks
8wks
16wks
Young Donor/ MA Recipients Tx
J
LSK
Absolute number - BM (x10^3)
LT HSC
ST HSC
MPP2
MPP3
MPP4
K
MP
L
Mature Thymocytes
Absolute number - Thymus (x10^6)
DP
CD4+
CD8+
M
Thymocyte Precursors
Absolute number - Thymus
ETP
DN2
DN3
DN4
N
Stromal analysis
Fibroblasts
Encothelial cells
mTEC
cTEC
DN TEC(aaTEC)
Absolute number (Thymus)

Supplementary Figure 4: Kit^{lo} HSCs exhibit enhanced thymic reconstitution capacity independent of recipient age. (A-H) Following allo-HCT (**as shown in Figure 2D**), frequency of donor-derived chimerism of hematopoietic cells **(A)**, mature lineages: Myeloid cells, B cells, and T cells **(B)**, in the PB at the indicated time points. **(C)** Enumeration of absolute number of donor-derived MP subset cells in the BM. All data are from n=10-11 mice/group (Kit^{hi}=10; Kit^{lo}= 11), across two independent experiments. **(D)** Relative expression of thymic-adhesion molecules (PSGL1, CCR7, and CCR9) on donor-derived CLPs (Statistical analysis was performed using ComBat[102] to perform batch-effect correction for the mean MFI across two experiments. Data are from n=8-9 mice/group, Kit^{lo}= 8; Kit^{hi}=9) across two independent experiments, **(E)** Absolute amount of intrathymic thymopoietic ligands, IL-22 (left), RANKL (right) was measured by ELISA after allo-HCT. All data are from n=4-5 mice/group (Kit^{hi}=4; Kit^{lo}= 5). **(F-G)** Frequency of donor-derived **(F)** CD4+ and **(G)** CD8+ T cell (naïve: CD62L+CD44- ; Central Memory: CD62L+CD44+; Effector Memory: CD62L-CD44+) subsets in the spleen. All data are from n=8-10 mice/group (Kit^{hi}=8; Kit^{lo}= 10), across two independent experiments. **(H-I)** Sixteen weeks following competitive HCT (**as shown in Figure 2P),** frequency of donor-derived chimerism of mature lineages, myeloid (**H**) and B cells (**I**) in the PB at the indicated timepoints. **(J-K)** Enumeration of absolute number of donor-derived LSK **(J) and** MP subset cells (**K**) in the BM**,** mature T cell **(L)** and thymocyte precursor subsets **(M)** in the thymi, and stromal cell subsets within thymic CD45- compartment **(O).** Refer to Supplementary Figure 12 for gating strategies to define above populations. All data are from n=6-7 mice/group, (Kit^{lo}= 7; Kit^{hi}=6), across two independent experiments. Error bars represent mean ± SEM. *P<0.05, **P<0.01, ***P<0.001, ****P<0.0001. P values calculated by nonparametric unpaired two-tailed Mann-Whitney U test. Source data are provided as a Source Data file, Source Data Supplementary Figure 4.

Young 20-weeks Post-HCT

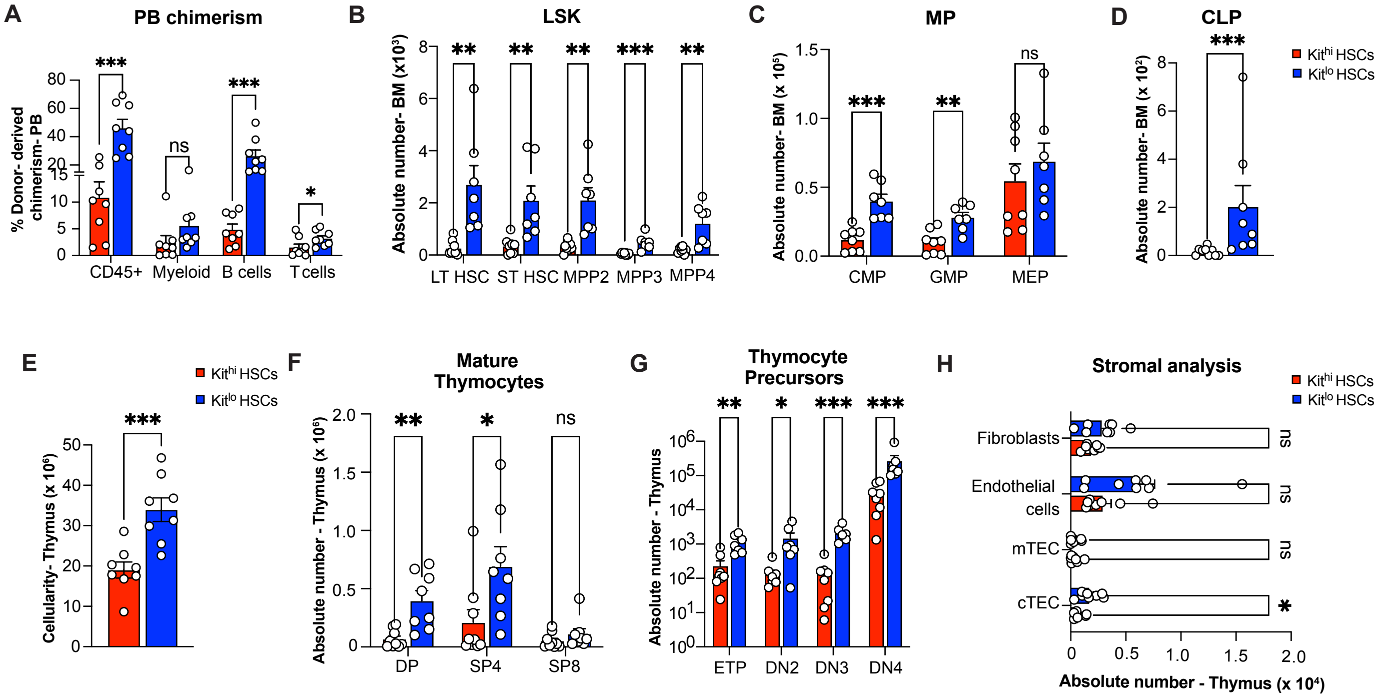

Supplementary Figure 5: Kit^{lo} HSCs maintain thymic reconstitution advantage long-term. Competitive allo-HCT using Kit subsets from young mice, as described in **Figure 2D**. Twenty weeks after competitive HCT, (**A**) frequency of donor-derived chimerism of mature lineages in the peripheral blood. Enumeration of absolute number of donor-derived cells in the BM for LSK cell subsets (**B**), MP cell subsets (**C**), CLP cells (**D**), total thymic cellularity (**E**), donor-derived mature T cells (**F**) and T cell precursor thymocyte subsets (**G**), thymic stromal compartment (CD45- cells) (**H**). Refer to Supplementary Figure 12 for gating strategies to define above populations. All data are from n=7-8 mice/group, (Kit^{lo}= 7; Kit^{hi}=8), across two independent experiments. Error bars represent mean ± SEM. *P<0.05, **P<0.01, ***P<0.001. P values calculated by nonparametric unpaired two-tailed Mann-Whitney U test. Source data are provided as a Source Data file, Source Data Supplementary Figure 5.

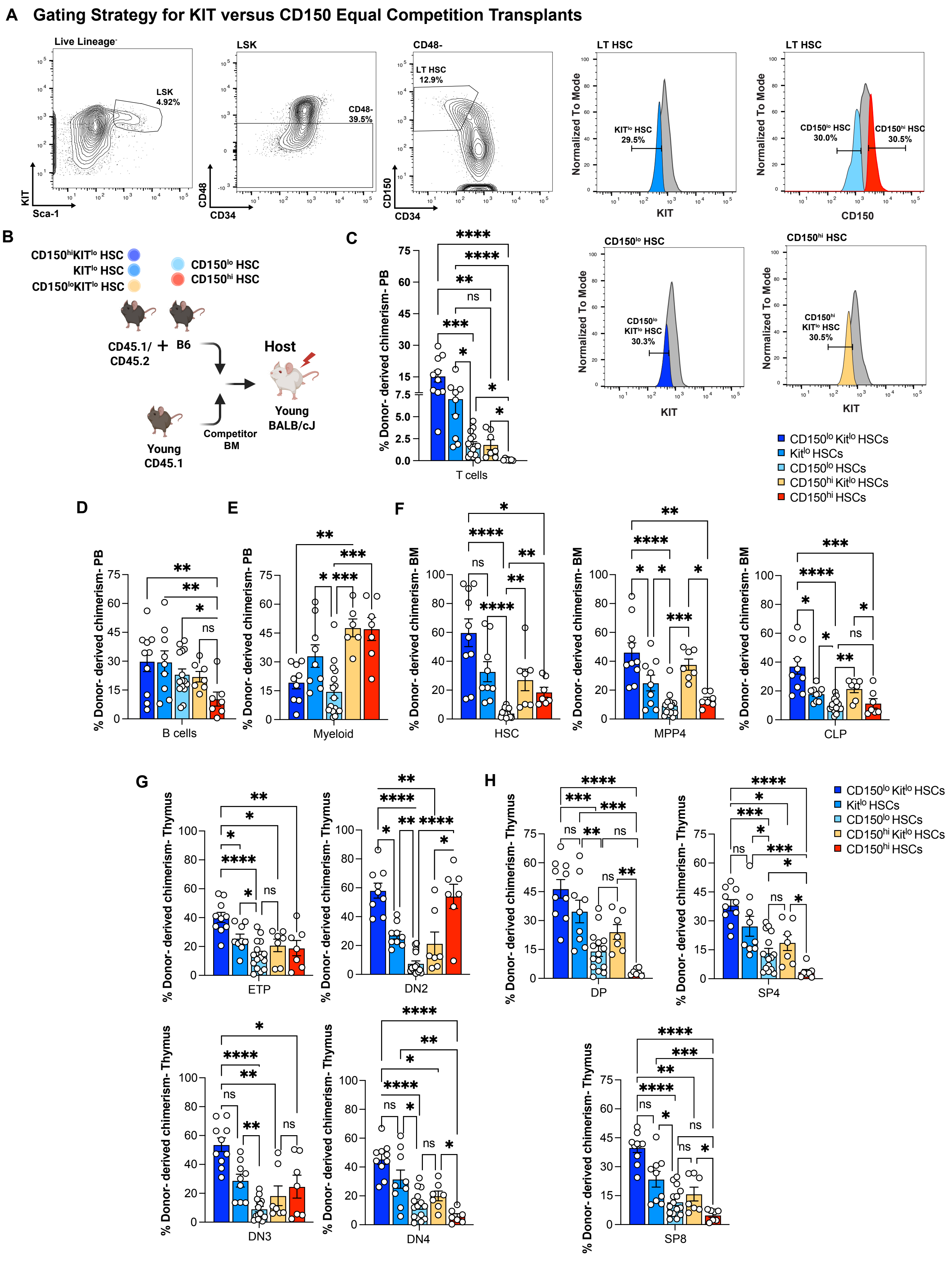
A Gating Strategy for KIT versus CD150 Equal Competition Transplants
Live Lineage-
LSK 4.92%
LSK
CD48- 39.5%
CD48-
LT HSC 12.9%
LT HSC
KIT^lo HSC 29.5%
CD150^lo HSC 30.0%
CD150^hi HSC 30.5%
Normalized To Mode
KIT
Sca-1
CD48
CD34
CD150
B
CD150^hiKIT^lo HSC
KIT^lo HSC
CD150^loKIT^lo HSC
CD150^lo HSC
CD150^hi HSC
CD45.1/ CD45.2
B6
Host
Young CD45.1
Competitor BM
Young BALB/cJ
C
% Donor- derived chimerism- PB
T cells
CD150^lo HSC
CD150^lo KIT^lo HSC 30.3%
CD150^hi HSC
CD150^hi KIT^lo HSC 30.5%
CD150^lo Kit^lo HSCs
Kit^lo HSCs
CD150^lo HSCs
CD150^hi Kit^lo HSCs
CD150^hi HSCs
D
E
F
B cells
Myeloid
% Donor- derived chimerism- BM
HSC
MPP4
CLP
G
H
% Donor- derived chimerism- Thymus
ETP
DN2
DP
SP4
DN3
DN4
SP8

Supplementary Figure 6: Comparative HSC subset analysis identifies optimal thymic reconstituting populations. Competitive allogeneic hematopoietic cell transplantation (allo-HCT) to compare thymic reconstitution capacity of different HSC subsets from young mice. **(A)** Representative FACS plots showing gating strategy used to isolate Kit^{lo}, $CD150^{lo}$, $Kit^{lo}CD150^{lo}$, $CD150^{hi}$, or $CD150^{hi}Kit^{lo}$ HSCs. (**B**) Experimental schema for competitive transplantation where equivalent numbers of each defined HSC subset from young donor mice (CD45.1/CD45.2 and CD45.2) were combined with competitor bone marrow (BM) cells from B6.SJL-PtprcaPepcb/BoyJ mice transplanted into lethally irradiated 7-week-old (young) BALB/cJ recipients. Six weeks after competitive HCT, frequency of donor-derived chimerism of mature lineages: T cells (**C**), B cells (**D**), and myeloid cells (**E**) in the peripheral blood as described in **Figure 2E**, donor-derived chimerism of HSC and lymphoid progenitor (LMPP and CLP) cell subsets in the BM as defined in **Figure 2F** (**F**), and donor-derived chimerism of T cell precursor thymocyte (**G**) and mature T cell subsets in the thymus as defined in Figures 3I-3J (**H**). Refer to Supplementary Figure 12 for gating strategies to define above populations. All data are from n=7-15 mice/group (Kit^{lo}=9, $CD150^{lo}$=15, $CD150^{lo}Kit^{lo}$=10, $CD150^{hi}$=7, and $CD150^{hi}Kit^{lo}$=7), across two independent experiments. Error bars represent mean ± SEM. *P<0.05, **P<0.01, ***P<0.001, ****P<0.0001. P values calculated by nonparametric unpaired two-tailed Mann-Whitney U test. Panel B was *Created in BioRender. Lab, K. (2025)* https://BioRender.com/oeh4i7x. Source data are provided as a Source Data file, Source Data Supplementary Figure 6.

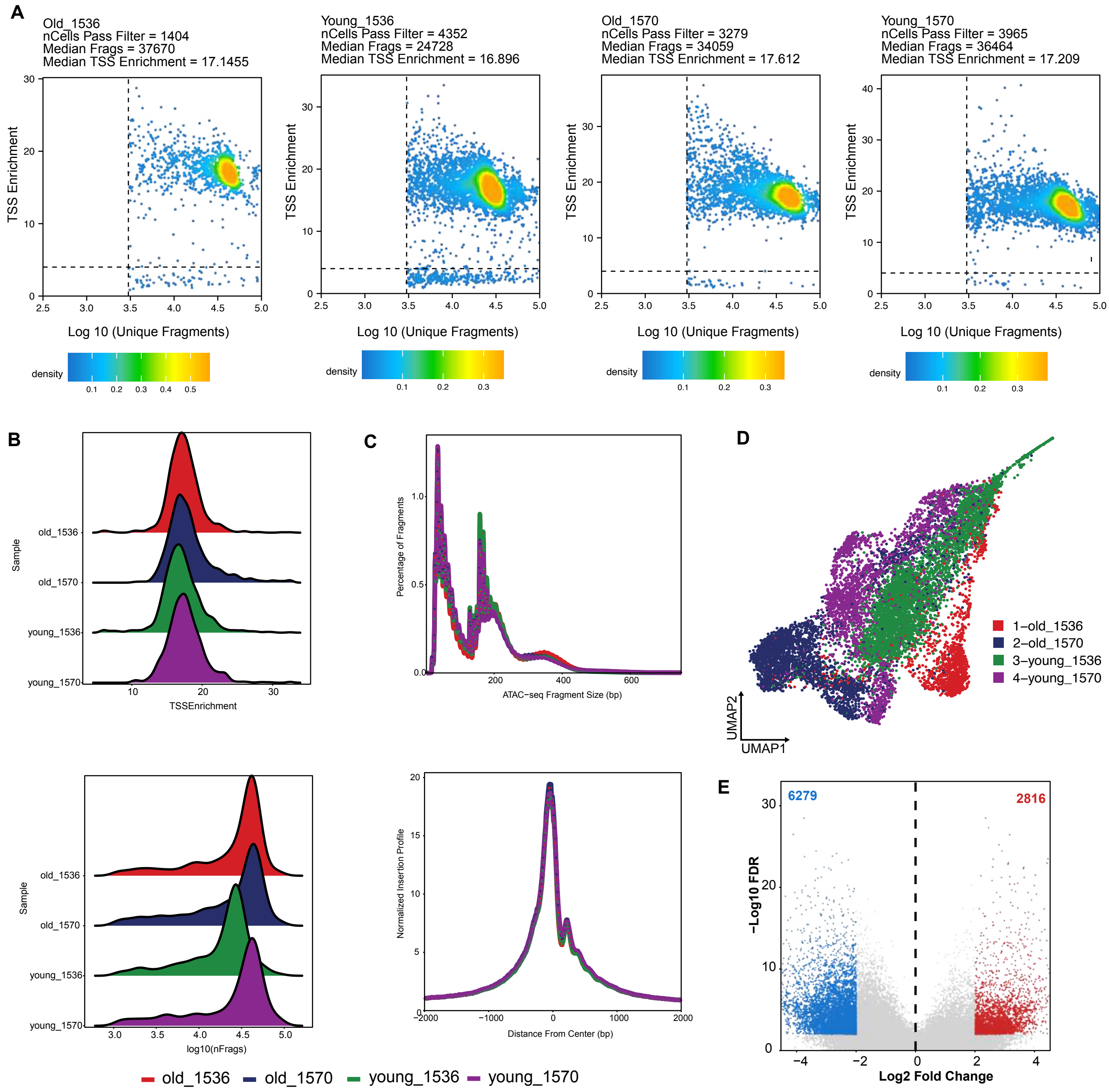

A
Old_1536
nCells Pass Filter = 1404
Median Frags = 37670
Median TSS Enrichment = 17.1455
Young_1536
nCells Pass Filter = 4352
Median Frags = 24728
Median TSS Enrichment = 16.896
Old_1570
nCells Pass Filter = 3279
Median Frags = 34059
Median TSS Enrichment = 17.612
Young_1570
nCells Pass Filter = 3965
Median Frags = 36464
Median TSS Enrichment = 17.209
TSS Enrichment
Log 10 (Unique Fragments)
density
B
Sample
old_1536
old_1570
young_1536
young_1570
TSSEnrichment
log10(nFrags)
C
Percentage of Fragments
ATAC−seq Fragment Size (bp)
Normalized Insertion Profile
Distance From Center (bp)
D
1−old_1536
2−old_1570
3−young_1536
4−young_1570
UMAP1
UMAP2
E
6279
2816
−Log10 FDR
Log2 Fold Change
old_1536 old_1570 young_1536 young_1570

Supplementary Figure 7: Single-cell ATAC seq pre-processing and QC analysis. (A) Comparison of TSS enrichment score and number of fragments. The dashed lines depict the cutoffs used. **(B)** Ridge plots showing distribution of TSS enrichment scores per sample (**top**) and distribution of unique nuclear fragments per sample (**bottom**). **(C)** Plots showing fragment size distribution (**top**) and TSS enrichment profile (**bottom**) across samples. (**D**) scATAC-seq UMAP without batch correction colored by sample. **(E)** Volcano plots of differentially accessible (DA) peaks (fold change> 2, FDR <0.01) for HSC subset Cluster 3 vs. Cluster 6 (Wilcoxon rank-sum test).

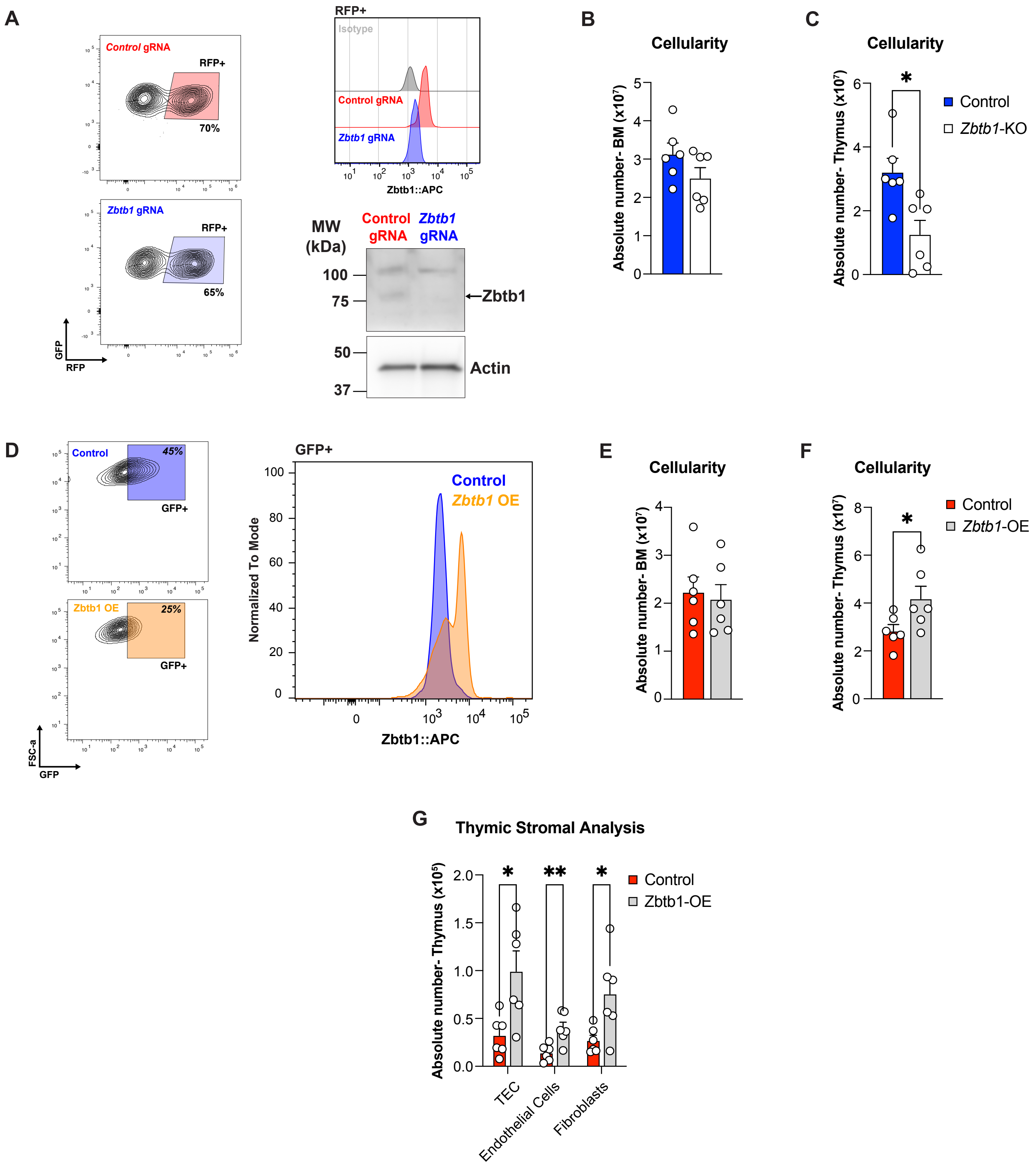

A
Control gRNA
RFP+
70%
Zbtb1 gRNA
RFP+
65%
GFP
RFP
RFP+
Isotype
Control gRNA
Zbtb1 gRNA
Zbtb1::APC
MW (kDa)
Control gRNA
Zbtb1 gRNA
100
75
50
37
←Zbtb1
Actin
B
Cellularity
Absolute number- BM (x10^7)
C
Cellularity
Absolute number- Thymus (x10^7)
Control
Zbtb1-KO
D
Control
45%
GFP+
Zbtb1 OE
25%
GFP+
FSC-a
GFP
GFP+
Control
Zbtb1 OE
Normalized To Mode
Zbtb1::APC
E
Cellularity
Absolute number- BM (x10^7)
F
Cellularity
Absolute number- Thymus (x10^7)
Control
Zbtb1-OE
G
Thymic Stromal Analysis
Absolute number- Thymus (x10^5)
Control
Zbtb1-OE
TEC
Endothelial Cells
Fibroblasts

Supplementary Figure 8: ZBTB1 drives HSC subset-specific thymic reconstitution capacity. (A) Zbtb1 expression assessed by flow cytometric and immunoblot analysis following knock out (KO) in Rosa26Cas9 KI Kit^{lo} HSCs. (**B-C**) Post-HCT analysis (as shown in Figure 4A) for BM cellularity (**B**) and thymic cellularity (**C**) All data are from n=6 mice/group, (Control = 6; *Zbtb1*-KO=6), across two independent experiments. (**D**) Zbtb1 expression assessed by flow cytometric and following OE of Zbtb1 cDNA in Kit^{hi} HSCs. (**E-G**) Post-HCT analysis (as shown in Figure 4F) for BM cellularity (**E**) and thymic analysis for cellularity (**F**) and thymic stromal compartment (CD45- cells) (**G**). All data are from n=6 mice/group, (Control = 6; *Zbtb1*-OE=6), across two independent experiments. Error bars represent mean ± SEM. *P<0.05, **P<0.01, ***P<0.001. P values calculated by nonparametric unpaired two-tailed Mann-Whitney U test. Source data are provided as a Source Data file, Source Data Supplementary Figure 8.

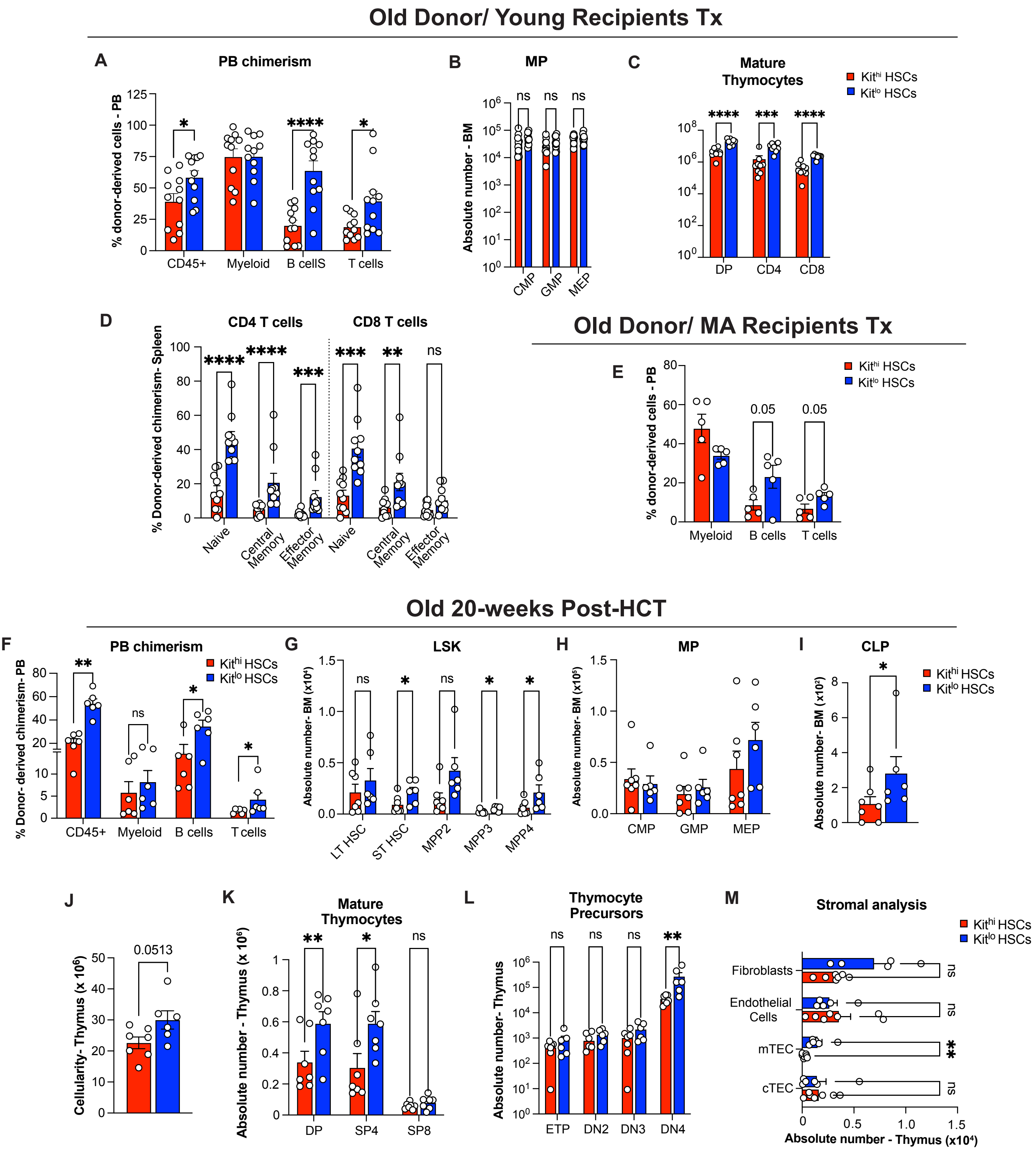
Old Donor/ Young Recipients Tx
A
PB chimerism
% donor-derived cells - PB
CD45+
Myeloid
B cellS
T cells
B
MP
Absolute number - BM
CMP
GMP
MEP
C
Mature Thymocytes
DP
CD4
CD8
Kithi HSCs
Kitlo HSCs
D
CD4 T cells
CD8 T cells
% Donor-derived chimerism- Spleen
Naive
Central Memory
Effector Memory
Old Donor/ MA Recipients Tx
E
% donor-derived cells - PB
Myeloid
B cells
T cells
0.05
Old 20-weeks Post-HCT
F
PB chimerism
% Donor- derived chimerism- PB
G
LSK
Absolute number- BM (x10⁴)
LT HSC
ST HSC
MPP2
MPP3
MPP4
H
MP
Absolute number- BM (x10⁵)
I
CLP
Absolute number- BM (x10²)
J
0.0513
Cellularity- Thymus (x 10⁶)
K
Mature Thymocytes
Absolute number - Thymus (x 10⁶)
SP4
SP8
L
Thymocyte Precursors
Absolute number- Thymus
ETP
DN2
DN3
DN4
M
Stromal analysis
Fibroblasts
Endothelial Cells
mTEC
cTEC
Absolute number - Thymus (x10⁴)
ns

Supplementary Figure 9: **Aged Kit^{lo} HSCs retain enhanced thymic reconstitution capacity.** (**A-F**) Eight weeks following competitive HCT (**as shown in Figure 5C**), frequency of donor-derived chimerism of mature lineages in the PB (Kit^{lo}= 11; Kit^{hi}=11) **(A)**. **(B-F)** Enumeration of absolute number of donor-derived MP subset cells in the BM (Kit^{hi}=10; Kit^{lo}= 10). **(B),** donor-derived mature T cells **(C),** and donor-derived CD4+ and CD8+ T cell subsets in the spleen (**D**) (Kit^{hi}=10; Kit^{lo}= 10). Aggregated data across two independent experiments. **(E)** Following allo-HCT (**as shown in Figure 5K**), frequency of donor-derived chimerism of mature lineages in the peripheral blood. Analyses were performed at 8 weeks following allo-HCT. All data are from n=5 mice/group (Kit^{lo}= 5; Kit^{hi}=5). **(F-M)** Competitive allo-HCT using Kit subsets from old mice, as described in **Figure 5C**. Twenty weeks after competitive HCT, (**F**) frequency of donor-derived chimerism of mature lineages in the peripheral blood. Enumeration of absolute number of donor-derived cells in the BM for LSK (**G**) and MP cell subsets (**H**), CLP cells (**I**), total thymic cellularity (**J**), donor-derived mature T cells (**K**) and T cell precursor thymocyte subsets (**L**), thymic stromal compartment (CD45- cells) (**M**). Refer to Supplementary Figure 12 for gating strategies to define above populations. All data are from n=6-7 mice/group, (Kit^{lo}= 6; Kit^{hi}=7). Aggregated data across two independent experiments. Error bars represent mean ± SEM. Error bars represent mean ± SEM. *P<0.05, **P<0.01, ***P<0.001, ****P<0.0001. P values calculated by nonparametric unpaired two-tailed Mann-Whitney U test. Source data are provided as a Source Data file, Source Data Supplementary Figure 9.

Equal Competition Tx

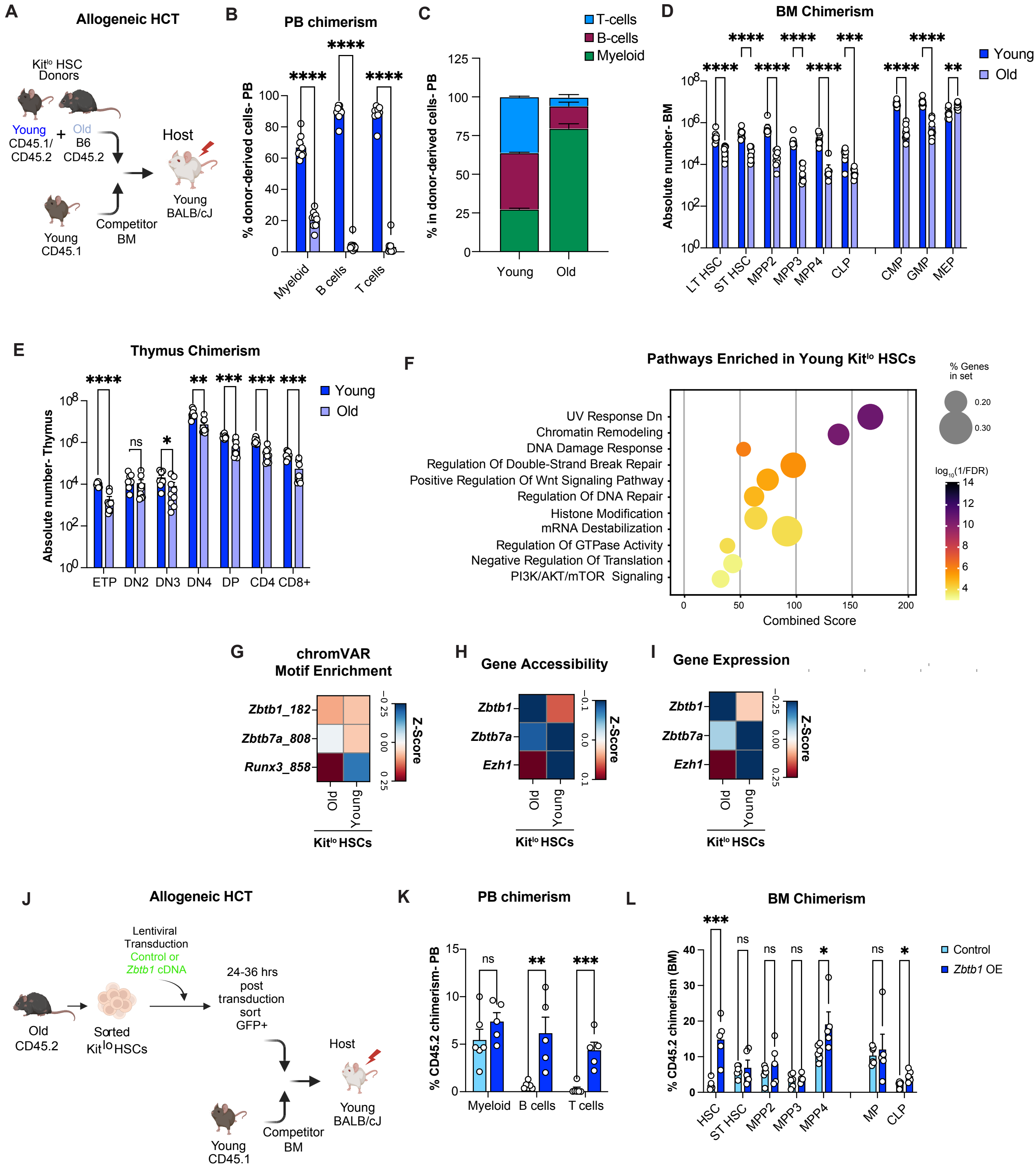

Supplementary Figure 10: Age-related functional decline and Zbtb1 rescue in Kit^{lo} HSCs. Equal Competition allo-HCT using Kit^{lo} HSCs from young and old mice. (**A**) Experimental schema for equal competition allogeneic HCT (allo-HCT) using Kit^{lo} HSCs from 2-mo young (blue) and 22-24-mo old (light blue) C57BL/6 mice combined with competitor bone marrow (BM) cells from B6.SJL-PtprcaPepcb/BoyJ mice transplanted into lethally irradiated 7-week-old (young) BALB/cJ recipients. Twenty weeks after competitive HCT, (**B**) frequency of donor-derived chimerism of mature lineages in the peripheral blood. (**C**) Lineage distribution in donor-derived CD45+ cells. Enumeration of absolute number of donor-derived cells in the BM for LSK and MP cell subsets (**D**), donor-derived T cell precursor thymocyte subsets and mature T cells (**E**). Refer to Supplementary Figure 12 for gating strategies to define above populations. All data are from n=9 mice/group (young= 9; old=9). Aggregated data across two independent experiments. Error bars represent mean ± SEM. (**F**) Pathway enrichment analysis performed on differentially expressed genes comparing old versus young Kit^{lo} HSCs by *gseapy*()[103] using Hallmark_2020 and GO_Biological_Process_2023 libraries. Bubble plot showing representative pathways enriched in young Kit^{lo} HSCs. (**G-I**) Matrix plots showing motif enrichment identified by ChromVAR (G), gene accessibility score (**H**), and gene expression (**I**) for Kit^{lo} subset in young and old HSCs. (J) Competitive allogeneic HCT with *Zbtb1*-OE Kit^{lo} HSCs generated with old mice. Ten weeks after competitive HCT, frequency of donor-derived chimerism of mature lineages in the peripheral blood (K) and donor-derived chimerism of LSK and MP cell subsets in the BM (L). Refer to Supplementary Figure 12 for gating strategies to define above populations. All data are from n=5-6 mice/group, (Control = 6; *Zbtb1*-OE=5), across two independent experiments. *$P<0.05$, **$P<0.01$, ***$P<0.001$. P values calculated by nonparametric unpaired two-tailed Mann-Whitney

U test. Panels A and J were *Created in BioRender. Lab, K. (2025)* [*https://BioRender.com/oeh4i7x*](https://BioRender.com/oeh4i7x). Source data are provided as a Source Data file, Source Data Supplementary Figure 10.

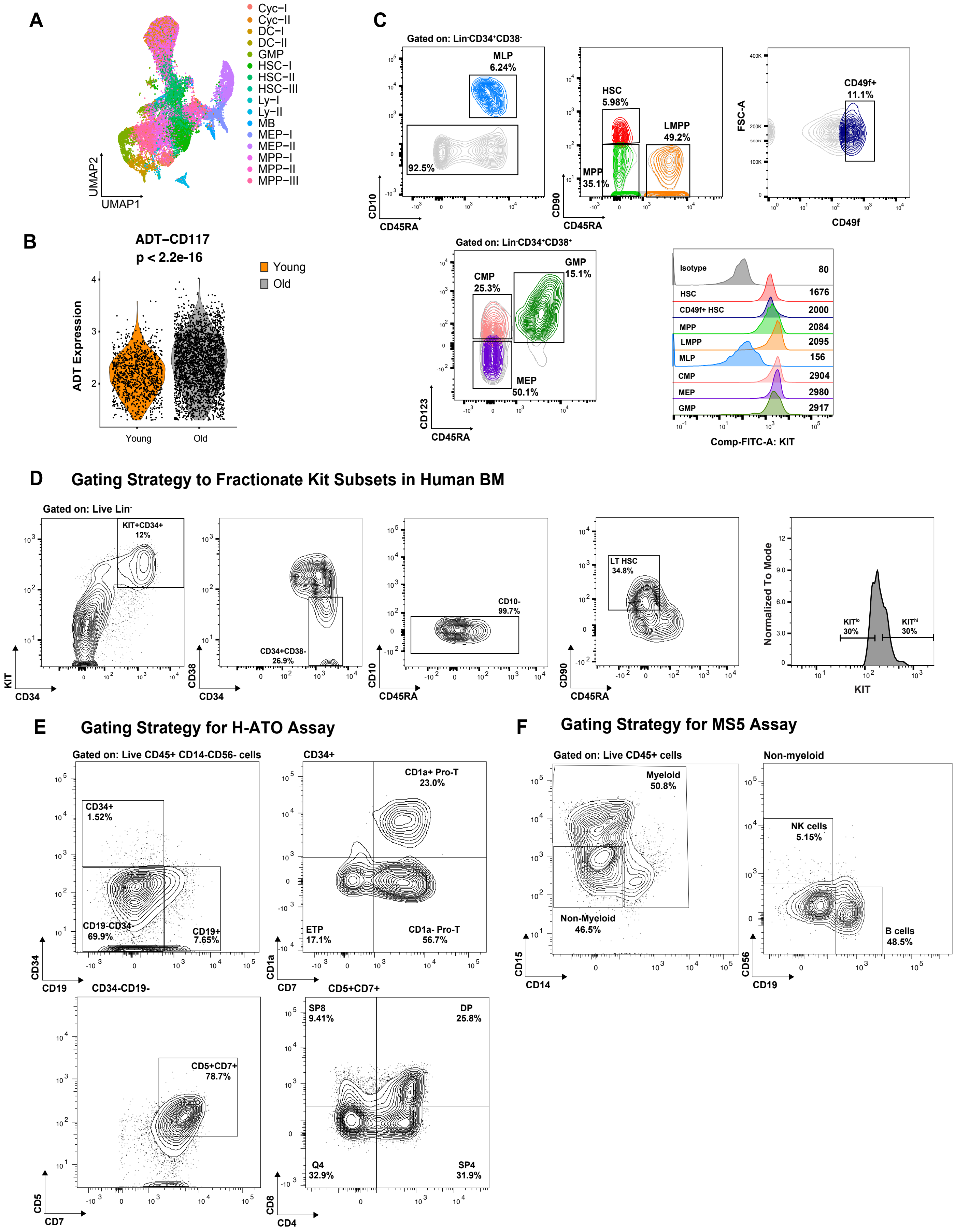
A
Cyc−I
Cyc−II
DC−I
DC−II
GMP
HSC−I
HSC−II
HSC−III
Ly−I
Ly−II
MB
MEP−I
MEP−II
MPP−I
MPP−II
MPP−III
UMAP1
UMAP2
B
ADT−CD117
p < 2.2e-16
Young
Old
ADT Expression
C
Gated on: Lin-CD34+CD38-
MLP 6.24%
92.5%
HSC 5.98%
LMPP 49.2%
MPP 35.1%
CD49f+ 11.1%
FSC-A
CD49f
CD10
CD45RA
CD90
Gated on: Lin-CD34+CD38+
CMP 25.3%
GMP 15.1%
MEP 50.1%
CD123
Isotype 80
HSC 1676
CD49f+ HSC 2000
MPP 2084
LMPP 2095
MLP 156
CMP 2904
MEP 2980
GMP 2917
Comp-FITC-A: KIT
D
Gating Strategy to Fractionate Kit Subsets in Human BM
Gated on: Live Lin-
KIT+CD34+ 12%
CD34+CD38- 26.9%
CD10- 99.7%
LT HSC 34.8%
KITlo 30%
KIThi 30%
Normalized To Mode
KIT
CD34
CD38
E
Gating Strategy for H-ATO Assay
Gated on: Live CD45+ CD14-CD56- cells
CD34+ 1.52%
CD19-CD34- 69.9%
CD19+ 7.65%
CD34+
CD1a+ Pro-T 23.0%
ETP 17.1%
CD1a- Pro-T 56.7%
CD34-CD19-
CD5+CD7+ 78.7%
CD5+CD7+
SP8 9.41%
DP 25.8%
Q4 32.9%
SP4 31.9%
CD19
CD1a
CD7
CD5
CD8
CD4
F
Gating Strategy for MS5 Assay
Gated on: Live CD45+ cells
Myeloid 50.8%
Non-Myeloid 46.5%
Non-myeloid
NK cells 5.15%
B cells 48.5%
CD15
CD14
CD56

Supplementary Figure 11: Human HSCs recapitulate Kit expression patterns and differentiation potential observed in mice. (A) UMAP of CD34+ young and old human BM CITE-seq[76] annotated with 15 clusters. **(B)** Violin plot for CD117 ADT-expression by age. Statistical analysis performed using Wilcoxon test. **(C)** Representative FACS plots of human BM samples showing gating strategy to define HSPC subsets. **(D)** Representative FACS plots of human BM samples showing gating strategy to define KIT^{hi} and KIT^{lo} HSCs in human BM. (E) Representative FACS plots of human artificial thymic organoid (H-ATO) co-culture assay showing gating strategy to define B cell and T cell developmental populations. First, based on a Live parent gate, viable Human hematopoietic cells were then identified by $hCD45^{+}$. Within the $hCD45^{+}$ population, cells were further gated as $CD14^{-}CD56^{-}$ to exclude monocytes and NK cells from the analysis. B cell and T cell lineages were then identified by CD19 expression as $hCD45^{+}CD14^{-}CD56^{-}CD19^{+}$ cells. T cell precursor populations were distinguished within the $CD34^{+}CD19^{-}$ compartment by differential expression of CD1a and CD7. Specifically, early thymic progenitors (ETP) were identified as $CD34^{+}CD1a^{-}CD7^{-}$, $CD1a^{-}$negative pro-T cells as $CD34^{+}CD1a^{-}CD7^{+}$, and $CD1a^{-}$positive pro-T cells as $CD34^{+}CD1a^{+}CD7^{+}$. Mature T cell populations were identified within the $CD34^{-}CD19^{-}$ compartment by co-expression of CD5, CD7, CD4, and CD8. Double positive thymocytes were identified as $CD34^{-}CD5^{+}CD7^{+}CD4^{+}CD8^{+}$, while single positive populations were identified as SP4 ($CD34^{-}CD5^{+}CD7^{+}CD4^{+}CD8^{-}$) and SP8 ($CD34^{-}CD5^{+}CD7^{+}CD4^{-}CD8^{+}$) cells. (F) Representative FACS plots of MS5 assay showing gating strategy to define human myeloid and lymphoid cell populations in MS5 co-culture differentiation assays. First, based on a Live parent gate, viable cells were identified. Human hematopoietic cells were then identified by positive expression of hCD45 ($hCD45^{+}$) to distinguish from MS5 stromal cells. Within the $hCD45^{+}$ population, differentiated myeloid and lymphoid lineages were assessed

by expression of lineage-specific markers to evaluate differentiation potential. Myeloid differentiation was assessed by identifying neutrophil-granulocytes as $hCD45^{+}CD15^{+}$ and monocyte-macrophages as $hCD45^{+}CD14^{+}$. Lymphoid differentiation was evaluated by identifying B cells as $hCD45^{+}CD19^{+}$ and NK cells as $hCD45^{+}CD56^{+}$.

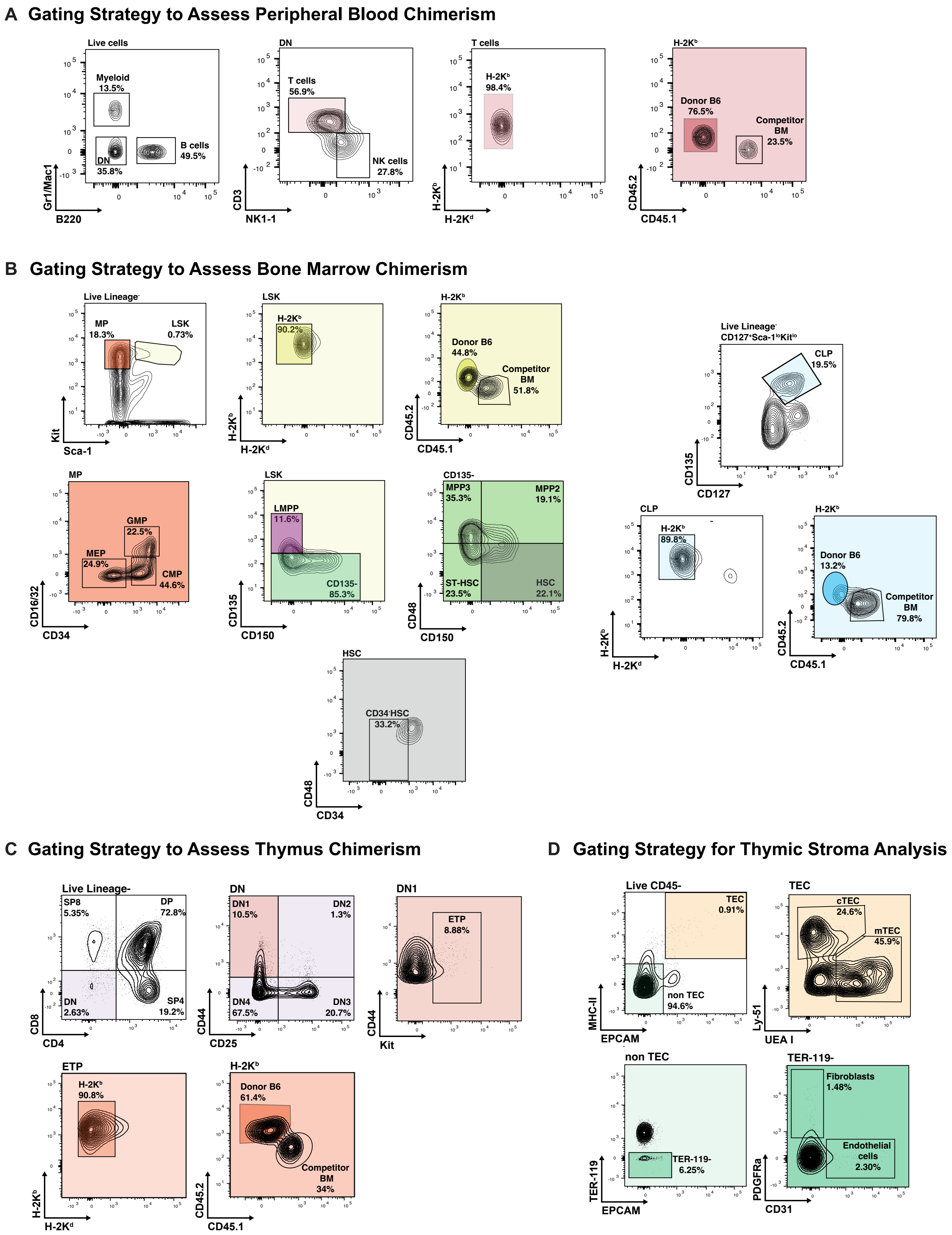

A Gating Strategy to Assess Peripheral Blood Chimerism
Live cells
Myeloid 13.5%
DN 35.8%
B cells 49.5%
Gr1/Mac1
B220
DN
T cells 56.9%
NK cells 27.8%
CD3
NK1-1
T cells
H-2Kᵇ 98.4%
H-2Kᵇ
H-2Kᵈ
H-2Kᵇ
Donor B6 76.5%
Competitor BM 23.5%
CD45.2
CD45.1
B Gating Strategy to Assess Bone Marrow Chimerism
Live Lineage-
MP 18.3%
LSK 0.73%
Kit
Sca-1
LSK
H-2Kᵇ 90.2%
H-2Kᵇ
H-2Kᵈ
H-2Kᵇ
Donor B6 44.8%
Competitor BM 51.8%
CD45.2
CD45.1
Live Lineage-
CD127+Sca-1loKitlo
CLP 19.5%
CD135
CD127
MP
GMP 22.5%
MEP 24.9%
CMP 44.6%
CD16/32
CD34
LSK
LMPP 11.6%
CD135- 85.3%
CD135
CD150
CD135-
MPP3 35.3%
MPP2 19.1%
ST-HSC 23.5%
HSC 22.1%
CD48
CD150
CLP
H-2Kᵇ 89.8%
H-2Kᵇ
H-2Kᵈ
H-2Kᵇ
Donor B6 13.2%
Competitor BM 79.8%
CD45.2
CD45.1
HSC
CD34-HSC 33.2%
CD48
CD34
C Gating Strategy to Assess Thymus Chimerism
Live Lineage-
SP8 5.35%
DP 72.8%
DN 2.63%
SP4 19.2%
CD8
CD4
DN
DN1 10.5%
DN2 1.3%
DN4 67.5%
DN3 20.7%
CD44
CD25
DN1
ETP 8.88%
CD44
Kit
ETP
H-2Kᵇ 90.8%
H-2Kᵇ
H-2Kᵈ
H-2Kᵇ
Donor B6 61.4%
Competitor BM 34%
CD45.2
CD45.1
D Gating Strategy for Thymic Stroma Analysis
Live CD45-
TEC 0.91%
non TEC 94.6%
MHC-II
EPCAM
TEC
cTEC 24.6%
mTEC 45.9%
Ly-51
UEA I
non TEC
TER-119- 6.25%
TER-119
EPCAM
TER-119-
Fibroblasts 1.48%
Endothelial cells 2.30%
PDGFRa
CD31

Supplementary Figure 12: FACS-based gating strategies for hematopoietic cell and stromal analysis following HCT. Representative FACS plots of Post HCT analysis to evaluate donor-derived chimerism in the Peripheral blood, Bone Marrow, and Thymus. (A) Gating strategy and identification of cell populations within the peripheral blood. First, based on a Live+ parent gate, hematopoietic cells were identified. Specifically, myeloid and B-cells were identified by expression of CD11b and Gr-1, while B cells expressed B-220. Then, double negative cells were assessed for CD3 expression to identify T cells. Within each lineage, cells were assessed for dual expression of CD45.2 and H-2Kb, while negative for H-2Kd and CD45.1, to identify donor-derived populations. (B) Gating strategy and identification of hematopoietic stem and progenitor cell populations within the bone marrow. First, based on a Live and Lineage (CD3, CD4, CD8, B220, CD11b, Gr-1, CD19, NK1.1, Ter119) negative parent gate, lineage^{-} hematopoietic precursor cells were identified. Specifically, LSK cells were identified by dual expression of Sca-1 and Kit, while MP cells expressed Kit but were negative for Sca-1. Within the LSK population, Flt3^{+}CD150^{-} cells were identified as MPP4. Furthermore, within the CD150^{+} compartment, CD150^{+}CD48^{-}, CD150^{+}CD48^{-}, CD150^{+}CD48^{+}, and CD150^{+}CD48^{+} cells were identified as HSC, ST-HSC, MPP2, and MPP3, respectively. Subsequently, HSC cells were assessed for CD34^{-}negative expression to identify LT-HSCs. Within the MP population, CD34^{-}CD16/32^{-}, CD34^{+}CD16/32^{-}, and CD34^{+}CD16/32^{+} cells were identified as MEP, CMP, and GMP, respectively. CLPs were first gated on Live Lin^{-}, then on Sca-1^{lo}Kit^{lo} cells. Within this Sca-1^{lo}Kit^{lo} population, CD127^{+}Flt3^{+} cells were identified as the entire CLP population. Within each stem and progenitor subset, cells were assessed for dual expression of CD45.2 and H-2Kb, while negative for H-2Kd and CD45.1, to identify donor-derived populations. (C) Gating strategy and identification of precursor and mature thymocytes within the thymus. First, based on a Live and

Lineage (B220, CD11b, Gr-1, CD19, NK1.1, Ter119, TCRgd) negative parent gate, lineage^{-} thymocytes were identified. T cell precursors were then assessed based on CD4 and CD8 expression to identify double negative (DN) populations. Within the DN compartment, precursor subsets were identified by differential expression of CD44, CD25, and Kit. Specifically, ETP cells were identified as $CD4^{-}CD8^{-}CD44^{+}CD25^{-}Kit^{+}$, DN2 cells as $CD4^{-}CD8^{-}CD44^{+}CD25^{+}$, and DN3 cells as $CD4^{-}CD8^{-}CD44^{-}CD25^{+}$. Mature T cell populations were identified by CD4 and CD8 co-expression patterns. Double positive (DP) thymocytes were identified as $CD4^{+}CD8^{+}$, while single positive populations were identified as SP4 ($CD4^{+}CD8^{-}$) and SP8 ($CD4^{-}CD8^{+}$) cells. Within each thymocyte subset, cells were assessed for dual expression of CD45.2 and H-2Kb, while negative for H-2Kd and CD45.1, to identify donor-derived populations. (D) Gating strategy for thymic stromal analysis. First, based on a Live and CD45^{-} parent gate, non-hematopoietic stromal cells were identified. Thymic epithelial cells (TEC) were then identified by positive expression of EpCAM ($CD45^{-}EpCAM^{+}$). Within the TEC population, cortical and medullary subsets were distinguished by differential expression of UEA-1, 6C3, and MHCII. Specifically, cortical TEC (cTEC) were identified as $CD45^{-}EpCAM^{+}UEA\text{-}1^{lo}6C3^{hi}MHCII^{hi/lo}$, while medullary TEC (mTEC) were identified as $CD45^{-}EpCAM^{+}UEA\text{-}1^{hi}6C3^{lo}MHCII^{hi/lo-}$. Non-epithelial stromal populations were identified within the $CD45^{-}EpCAM^{-}$ compartment after excluding Ter-119^{-} cells. Endothelial cells were identified as $CD45^{-}EpCAM^{-}Ter\text{-}119^{-}PDGFR\alpha^{-}CD31^{+}$, while fibroblasts were identified as $CD45^{-}EpCAM^{-}Ter\text{-}119^{-}CD31^{-}PDGFR\alpha^{+}$ cells.

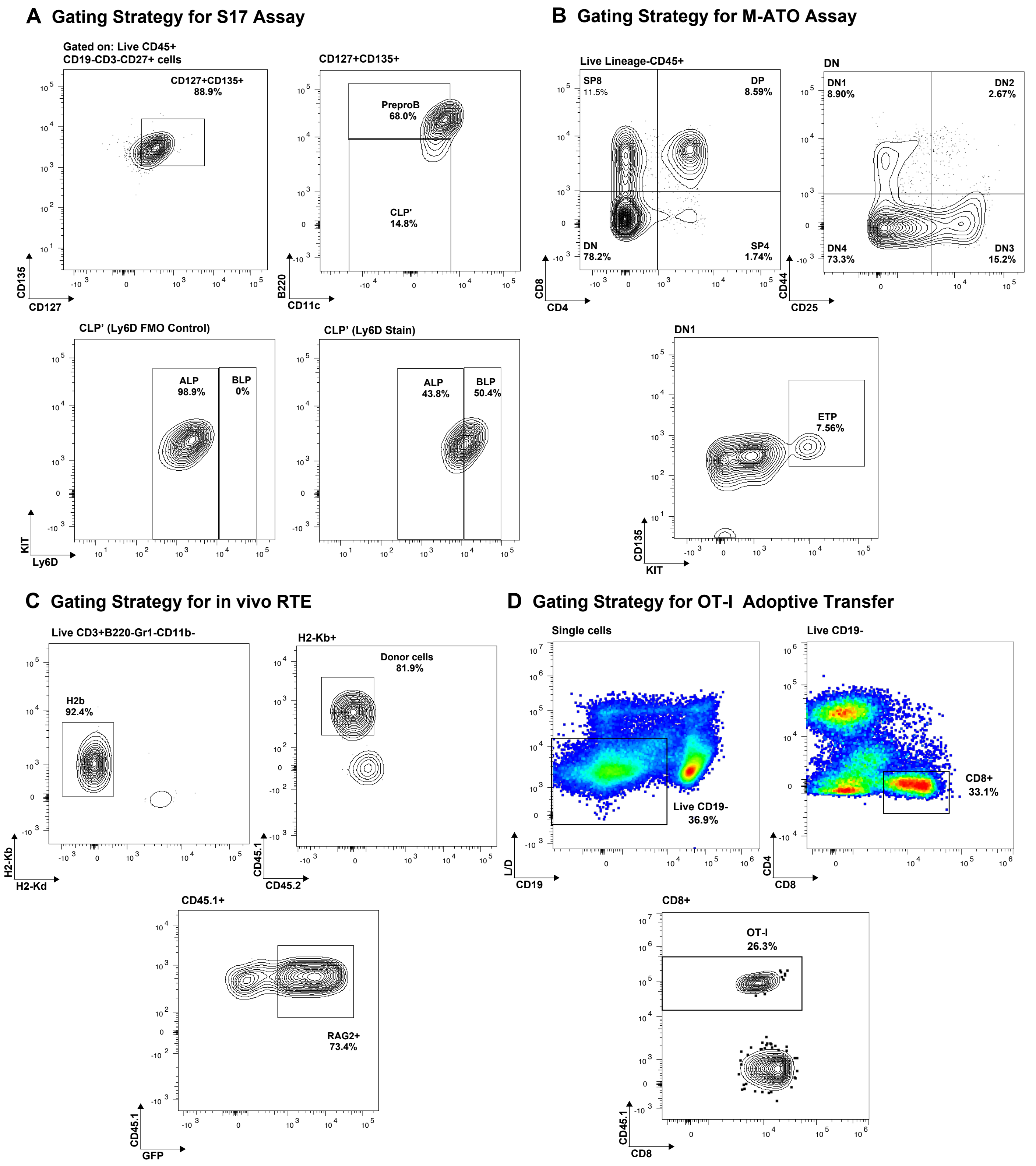

A Gating Strategy for S17 Assay
Gated on: Live CD45+ CD19-CD3-CD27+ cells
CD127+CD135+ 88.9%
CD135
CD127
CD127+CD135+
PreproB 68.0%
CLP' 14.8%
B220
CD11c
CLP' (Ly6D FMO Control)
ALP 98.9%
BLP 0%
CLP' (Ly6D Stain)
ALP 43.8%
BLP 50.4%
KIT
Ly6D
B Gating Strategy for M-ATO Assay
Live Lineage-CD45+
SP8 11.5%
DP 8.59%
DN 78.2%
SP4 1.74%
CD8
CD4
DN
DN1 8.90%
DN2 2.67%
DN4 73.3%
DN3 15.2%
CD44
CD25
DN1
ETP 7.56%
CD135
KIT
C Gating Strategy for in vivo RTE
Live CD3+B220-Gr1-CD11b-
H2b 92.4%
H2-Kb
H2-Kd
H2-Kb+
Donor cells 81.9%
CD45.1
CD45.2
CD45.1+
RAG2+ 73.4%
CD45.1
GFP
D Gating Strategy for OT-I Adoptive Transfer
Single cells
Live CD19- 36.9%
L/D
CD19
Live CD19-
CD8+ 33.1%
CD4
CD8
CD8+
OT-I 26.3%
CD45.1
CD8

Supplementary Figure 13: FACS-based gating strategies for in vitro co-culture assays, recent thymic emigrant analysis, and adoptive T cell transfer studies. (A) Representative FACS plots of mouse S17 co-culture assay showing gating strategy to define lymphoid precursor populations. (B) Representative FACS plots of mouse artificial thymic organoid (M-ATO) co-culture assay showing gating strategy to define T cell developmental populations. (C) Representative FACS plots of post-HCT analysis to define recent thymic emigrant populations (RTEs; $RAG2^+$ T cells). (D) Representative FACS plots of adoptive OT-I T cell transfer analysis to evaluate donor-derived T cell functionality.

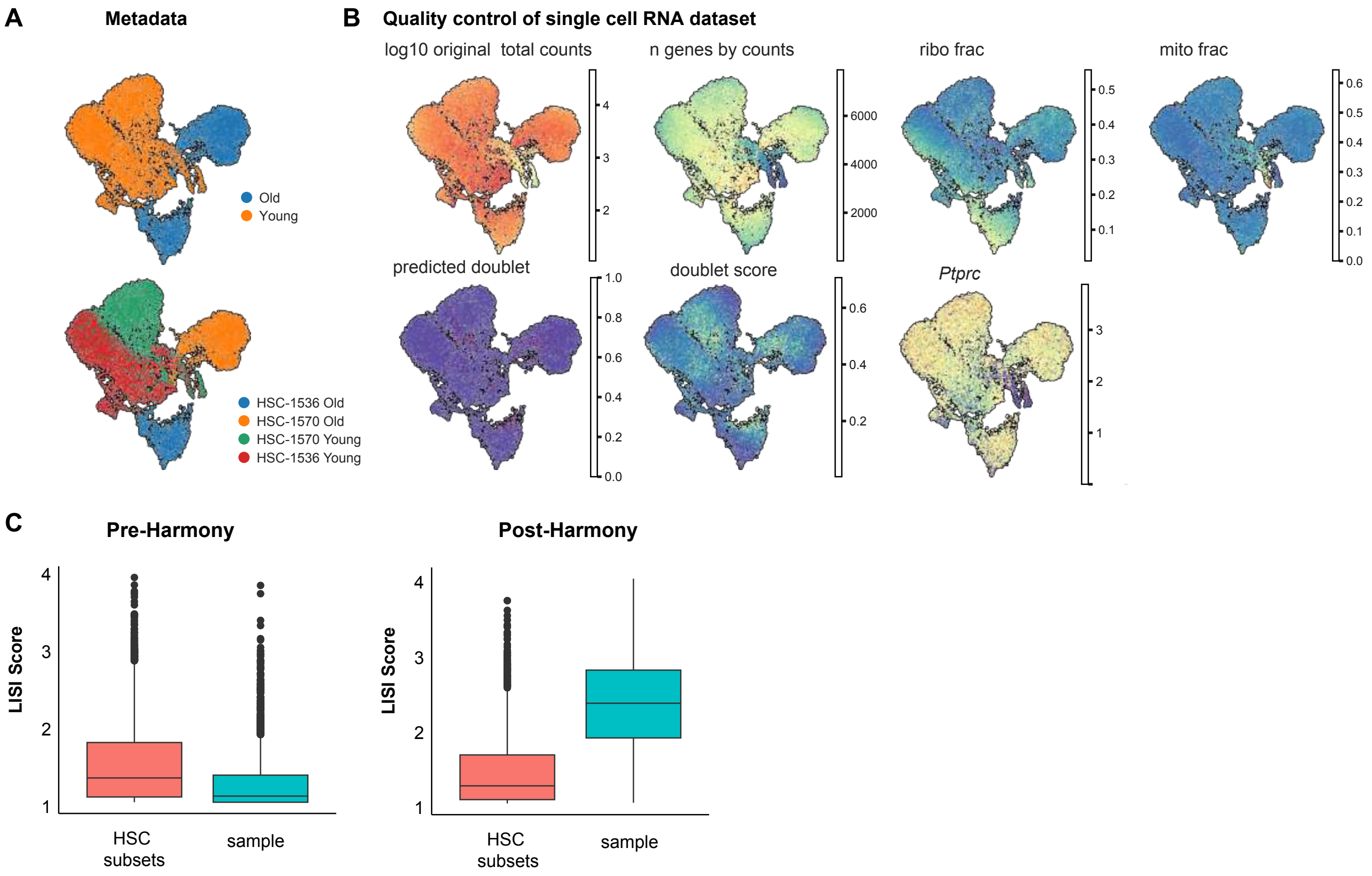
A
Metadata
Old
Young
HSC-1536 Old
HSC-1570 Old
HSC-1570 Young
HSC-1536 Young
B
Quality control of single cell RNA dataset
log10 original total counts
n genes by counts
ribo frac
mito frac
predicted doublet
doublet score
Ptprc
C
Pre-Harmony
Post-Harmony
LISI Score
HSC subsets
sample

Supplementary Figure 14: Single-cell RNA sequencing quality control and batch correction validation. (A) UMAPs of 14,672 cells from 2mo (young) or 22-24mo (old) HSCs prior to quality control. UMAPs in sequence display age cohort (top) and origin sample (Metadata info; bottom). (B) QC info in sequence displays total counts in log10 scale, number of genes, ribosomal fraction, mitochondrial fraction, predicted doublet and doublet per cell (QC info); CD45- (non-hematopoietic) contaminating cells expressing Ptprc (Contaminants). (C) Boxplots of LISI scores to assess quality of pre harmony (left) and post-harmony (right) integration based on HSC subsets (cell-type) and samples (biological replicates).
