# Supplementary material for: Kitlo hematopoietic stem cells exhibit distinct lymphoid-primed chromatin landscapes that enhance thymic reconstitution
Source: Nat Commun. 2025 Jul 4;16:6170. doi: 10.1038/s41467-025-61125-1 (PMC12227609; doi:10.1038/s41467-025-61125-1)
Supplement: Supplementary file 2 — Description of Additional Supplementary Files [file 41467_2025_61125_MOESM2_ESM.pdf]

Supplementary Data 1: Top marker genes for each HSC subset.

Supplementary Data 2: Dataset of differentially expressed genes (DEGs) comparing young and old HSCs, HSC subsets, and young and old Kit^{lo} HSCs at steady state.

Supplementary Data 3: Zbtb1-targets enriched in Kit^{lo} HSCs

Supplementary Data 4: Pathway enrichment analysis of Non-Notch1 Zbtb1 targets enriched in Kit^{lo} HSCs

Supplementary Data 5: Pathway enrichment analysis of differentially expressed genes comparing young and old Kit^{lo} HSCs

Supplementary Data 6: Human Bone Marrow Donor Information

Supplementary Data 7: Reagents and Animal Strains
