# Supplementary material for: Kitlo hematopoietic stem cells exhibit distinct lymphoid-primed chromatin landscapes that enhance thymic reconstitution
Source: Nat Commun. 2025 Jul 4;16:6170. doi: 10.1038/s41467-025-61125-1 (PMC12227609; doi:10.1038/s41467-025-61125-1)
Supplement: Supplementary file 10 — Reporting Summary [file 41467_2025_61125_MOESM10_ESM.pdf]

nature portfolio

NCOMMS-24-37587B
Corresponding author(s): Drs. Harold K. Elias, Michael G. Kharas, and Marcel R.M. van den Brink
Last updated by author(s): 05-31-2025

Reporting Summary

Nature Portfolio wishes to improve the reproducibility of the work that we publish. This form provides structure for consistency and transparency in reporting. For further information on Nature Portfolio policies, see our Editorial Policies and the Editorial Policy Checklist.

Statistics

For all statistical analyses, confirm that the following items are present in the figure legend, table legend, main text, or Methods section.

n/a	Confirmed	
[ ]	[x]	The exact sample size (*n*) for each experimental group/condition, given as a discrete number and unit of measurement
[ ]	[x]	A statement on whether measurements were taken from distinct samples or whether the same sample was measured repeatedly
[ ]	[x]	The statistical test(s) used AND whether they are one- or two-sided<br>*Only common tests should be described solely by name; describe more complex techniques in the Methods section.*
[x]	[ ]	A description of all covariates tested
[ ]	[x]	A description of any assumptions or corrections, such as tests of normality and adjustment for multiple comparisons
[ ]	[x]	A full description of the statistical parameters including central tendency (e.g. means) or other basic estimates (e.g. regression coefficient) AND variation (e.g. standard deviation) or associated estimates of uncertainty (e.g. confidence intervals)
[x]	[ ]	For null hypothesis testing, the test statistic (e.g. *F*, *t*, *r*) with confidence intervals, effect sizes, degrees of freedom and *P* value noted<br>*Give P values as exact values whenever suitable.*
[x]	[ ]	For Bayesian analysis, information on the choice of priors and Markov chain Monte Carlo settings
[x]	[ ]	For hierarchical and complex designs, identification of the appropriate level for tests and full reporting of outcomes
[ ]	[x]	Estimates of effect sizes (e.g. Cohen's *d*, Pearson's *r*), indicating how they were calculated

Our web collection on statistics for biologists contains articles on many of the points above.

Software and code

Policy information about availability of computer code

Data collection

Single Cell Multiome ATAC and Gene Expression Cell Preparation
Single Cell Multiome ATAC + Gene Expression was performed with the 10X genomics system using Chromium Next GEM Single Cell Multiome Reagent Kit A (catalog no. 1000282) and ATAC Kit A (catalog no. 1000280) following Chromium Next GEM Single Cell Multiome ATAC + Gene Expression Reagent Kits User Guide and demonstrated protocol - Nuclei Isolation for Single Cell Multiome ATAC + Gene Expression Sequencing. Briefly, cells (viability 95%) were lysed for 4min and resuspended in Diluted Nuclei Buffer (10x Genomics, PN- 2000207). Lysis efficiency and nuclei concentration was evaluated on Countess II automatic cell counter by trypan blue staining. Nuclei were loaded per transposition reaction, with a targeting recovery between 1,000 and 10,000 nuclei after encapsulation. After transposition reaction nuclei were encapsulated and barcoded. Next-generation sequencing libraries were constructed following User Guide, which were sequenced on an Illumina NovaSeq 6000 system.

Data analysis

SEQUENCING DATA PROCESSING
Single Cell RNA-seq
i) Preprocessing and downstream data analysis
FASTQ files were processed using the 10x Cell Ranger package (v7.01). The Cell Ranger generated filtered_feature_bc_matrix.h5 files were processed following the guidelines on the shunPykeR GitHub repository(https://github.com/kousaa/shunPykeR), an assembled pipeline of publicly available single cell analysis packages put in coherent order, that allows for data analysis in a reproducible manner and seamless usage of Python and R code. Genes that were not expressed in any cell and ribosomal and hemoglobin genes were removed from downstream analysis. Each cell was then normalized to a total library size of 10,000 reads and gene counts were log-transformed using the log (X+1) formula, in which log denotes the natural logarithm. Principal component analysis (components =20) was applied to reduce noise prior to data clustering. To select the optimal number of principal components to retain for each dataset, the knee point (eigenvalues smaller

radius of curvature) was used. Leiden clustering90 (resolution = 0.9) was used to identify clusters within the PCA-reduced data.

Quality of the single cells (Supplementary Figures 13A-B) was computationally assessed based on total counts, number of genes, mitochondrial and ribosomal fraction per cell, with low total counts, low number of genes (≤1000) and high mitochondrial content (≥0.2) as negative indicators of cell quality. Cells characterized by more than one negative indicator were considered as "bad" quality cells. Although cells were negatively sorted prior to sequencing for the CD45 marker, a small amount of non-hematopoietic cells (expressing no Ptprc), were detected within our dataset. To remove bad quality cells and contaminants in an unbiased way, we assessed them on a cluster basis rather than individually. Leiden clusters with a "bad" quality profile and/or a high number of contaminating cells were removed. Finally, cells marked as doublets by scrublet91 were also filtered out. Overall, a total of 2325 cells, representing ~8.4% of all our data, was excluded from further analysis (see Figure S2 for per sample metrics). After removal of these cells, we calculated highly variable genes (HVG=3000) and re-performed PCA with unsupervised clustering analysis (components =20), followed by batch effect correction across all samples using harmony92 to assist annotation of cell type subsets within the dataset, using sample as the batch key.

Batch effect correction was performed using Harmony with default parameters, using sample identity as the batch key. To assess integration quality, we used the diversity score, specifically the Local Inverse Simpson's Index (iLISI).92 Prior to Harmony integration, analysis of our young and old samples revealed an integration score (iLISI) of 1.07, which indicates poorly integrated samples, and a cell-type identity score (cLISI) of 1.31. After Harmony integration, we observed a high median iLISI score of 2.3 when combining our old and young samples, which is indicative of well-integrated biological replicates. Additionally, we evaluated accuracy by examining the retention of cell-type distinctions post-integration, where the median cell-type LISI (cLISI) was 1.23, reflecting that our integration preserved biological differences among cell types effectively (Supplementary Figure 13C). To transfer the 'HSC subset' annotations from our young reference dataset to the aged HSC dataset, we employed scanpy's ingest function (sc.tl.ingest()) using default parameters. The integration was performed using pre-computed UMAP embedding coordinates generated from our young dataset as the spatial reference and the 'HSC_subtype' observation key for cell type annotation transfer.

ii) Defining Kitlo and Kithi HSC subsets

Owing to the significant drop-out effect in scRNA-seq data,93 it is impossible to ascertain if a cell has low/mid (and sometimes high) Kit expression versus no expression. To methodically deal with this, we performed _data denoising and_ imputation using the MAGIC (Markov Affinity-based Graph Imputation of Cells) method,94 to denoise distinguish between dropouts and genuinely low gene expression values. Post-MAGIC imputation, we proceeded to set the thresholds for Kit, specifically, we calculated the 20th and 80th percentile expression value across all cells. Subsequently, cells were classified into Kitlo (Kit expression below the 20th percentile), Kithi (Kit expression above the 80th percentile), and Kitmid (Kit expression between the 20th and 80th percentile). This approach helped to enhance the quality of the data by denoising and imputing the missing values, thereby providing a more accurate representation of the gene expression landscape.

iii) Differential expression analysis

Differential expression analysis for comparisons of interest was performed with MAST (Model-based Analysis of Single-cell Transcriptomics) using the likelihood ratio test.95 In all cases, differentially expressed genes were considered statistically significant if the FDR-adjusted p-value was less than 0.05. Gene set score analysis between comparison groups was performed using the sc.tl.scoregenes() function from scanpy. This function calculates cluster-specific gene signatures by computing average expression scores and subtracting scores from a randomly sampled reference gene set. To ensure robust results, we implemented a detection threshold requiring each differentially expressed gene to be present in at least 10% of cells within each analyzed cluster. The comprehensive results of this analysis, including detailed per-cluster gene expression percentages, can be found in 'Supplementary Data table 2_DEG analysis'.

iv) Differential abundance (DA) analysis

DA HSC subset across age groups was identified by sampling neighborhoods of cells from a k-nearest neighbors (k-NN) graph and looking for enrichment of either age in each neighborhood as implemented in MiloR.96 MiloR is a graph-based statistical method to compute differential cellular abundances in neighborhoods of cells. The 15 batch-corrected latent dimensions from scanpy were used for MiloR (v.0.99.19) k-NN graph construction (k=35) and neighborhood indexing (proportion=0.1). DA testing was performed with generalized linear models, including age as covariates (neighborhoods significant if spatial corrected FDR<0.25).

Single- Cell ATAC sequencing

Preprocessing, dimensionality reduction, clustering

Single-cell ATAC-seq from young and old mice were aligned to the mm10 genome and we processed the cellranger output file, fragments.tsv, with ArchR (v.1.0.2)97 was used for downstream analysis. We performed QC filtering on scATAC-seq using ArchR with the default parameters of createArrowFiles() ((Supplementary Figure 7). We retained cells containing at least 1,000 and at most 100,000 fragments. Next, we filtered out the cells that did not pass QC in the corresponding scRNA-seq data, thus retaining 11493 cells common to both scRNA-seq and scATAC-seq. We then performed dimensionality reduction using iterative latent semantic indexing (LSI) on the top 25,000 variable features from the tile matrix to get a reduced dimensionality of 30 components with addIterativeLSI() function in ArchR. To generate visualizations, we employed the addUMAP() function in ArchR with the following settings: nNeighbors=20; minDist=0.1. For clustering, we utilized the addClusters() function in ArchR, specifying the parameters as follows: method='Seurat', knnAssign=10, and maxClusters=10. Next, we used Harmony92 to perform batch effect correction and using sample as the batch key.

Peak-calling and TF motif accessibility scoring

Each individual sample was pseudo bulked for peak calling with MACS298 peak caller and iterative peak overlapping removal within ArchR (using default settings). We subsequently added motif annotations using addMotifAnnotations() with the CisBP motif database and computed chromVAR99 deviations for each single cell with addDeviationsMatrix(). To identify differentially accessible motifs within each group of interest, we applied the rank_genes_groups() function in scanpy. With the following settings: method='wilcoxon' and corr_method = 'benjamini-hochberg' and performed the analysis on the chromVAR zscore matrix.

Processing Human Single-Cell CITE-seq

We analyzed the young human bone marrow CITE-seq from Sommarin et al.76 using Seurat.100 Authors provided the peak matrix aligned to the hg38 genome. We used the sample BM_34 for which the authors provided the cell type annotations. We processed the scRNA-seq and CITE-seq data for young and old bone marrow using Seurat. The hash-tagged cells were demultiplexed using HTODemux() from Seurat. The cell type annotations were provided by the authors for young bone marrow samples labeled yBM1_hpc and yBM2_hpc. We predicted cell type annotations for the old bone marrow cells by label transfer using FindTransferAnchors() and TransferData() functions. We obtained the Kitlo gene signature from our mouse scRNAseq data through differential gene expression analysis. To extend this signature to our human bone marrow HSPC dataset, we used Ensembl101 Biomart to identify human orthologues of the mouse genes. Subsequently, we scored the Kitlo gene signature in human scBM HSCs using Seurat's AddModuleScore() function.

For manuscripts utilizing custom algorithms or software that are central to the research but not yet described in published literature, software must be made available to editors and reviewers. We strongly encourage code deposition in a community repository (e.g. GitHub). See the Nature Portfolio guidelines for submitting code & software for further information.

Data

Policy information about availability of data

All manuscripts must include a data availability statement. This statement should provide the following information, where applicable:

- Accession codes, unique identifiers, or web links for publicly available datasets
- A description of any restrictions on data availability
- For clinical datasets or third party data, please ensure that the statement adheres to our policy

All data are included in the Supplementary Information or available from the authors, as are unique reagents used in this Article. The raw numbers for charts and graphs are available in the Source Data file whenever possible. Raw Western blot images (related to Supplementary Figure 8A) are available at https://doi.org/10.6084/m9.figshare.28899050. The Multiome single- cell RNA and ATAC data generated in this study have been deposited in the GEO database under accession code GSE246464. Mouse CITE-seq data used in this study are available in the GEO database under accession code GSE243197 ((related to Supplementary Figures 2F-2G, Solomon et al. JEM 2024). Human bone marrow CITE-seq data from young and old donors used in this study are available at OSF Archive (Related to Figure 6, Sommarin et al. Biorxiv 2021). The processed multiome data are available at zenodo (https://zenodo.org/uploads/15521122). The raw numbers for charts and graphs are available in the Source Data file whenever possible.

Research involving human participants, their data, or biological material

Policy information about studies with human participants or human data. See also policy information about sex, gender (identity/presentation), and sexual orientation and race, ethnicity and racism.

Reporting on sex and gender	All patient derived materials used in this study were selected randomly and hence biological gender was not taken into consideraton. Race, ethnicity or other socially relevant groupings were not taken under consideration as all samples from patients were randomly selected.
Reporting on race, ethnicity, or other socially relevant groupings	For this study, human BM samples are randomly selected for this study and collected from age range of 23-60 and include only females to match our mouse studies.
Population characteristics	For this study, human BM samples are randomly selected for this study and collected from age range of 23-60 and include only females to match our mouse studies.
Recruitment	BM samples were obtained from BioIVT, LLC
Ethics oversight	Healthy human bone marrow mononuclear cells (Supplementary Data Table 6) were purchased from BIOIVT (Johnson City, TN). The commercial human material obtained from BIOIVT was approved by SERATRIALS, LLC under Protocol No. 2010-017, IRB Tracking No. 20161665. All approved healthy volunteers provided informed written consent through SERATRIALS, LLC- a wholly owned subsidiary of BioIVT that acts as the sponsor of biospecimen collections and conducts human research activities in accordance with regulations surrounding human subject safety and protection, including ethical principles originating from the Declaration of Helsinki and consistent with Good Clinical Practice guidelines.

Note that full information on the approval of the study protocol must also be provided in the manuscript.

Field-specific reporting

Please select the one below that is the best fit for your research. If you are not sure, read the appropriate sections before making your selection.

[x] Life sciences [ ] Behavioural & social sciences [ ] Ecological, evolutionary & environmental sciences

For a reference copy of the document with all sections, see nature.com/documents/nr-reporting-summary-flat.pdf

Life sciences study design

All studies must disclose on these points even when the disclosure is negative.

Sample size	For invivo competitive transplants, sample sizes were determined based on previous experience for providing high quality reproducible results. Sufficient "N" number is used to obtain reliable statistical results. All mouse in vitro studies, n=5; human in vitro studies, n=5-8 independent BM samples; mouse in vivo studies, n=5-10, for all experimental and control groups. Sample size across each experiment represented in our figures is provided in their respective figure legends in our manuscript.
Data exclusions	No data exclusions were done
Replication	All in vitro experiments were conducted across three independent experiments either in 2-3 technical replicates.. In vivo experiments were conducted across two independent experiments Consistent results were obtained even during confirmation or preliminar
Randomization	Recipient mice were randomized based on body weight to ensure comparable weights (20-22g) between experimental groups prior to transplantation.

Blinding | The authors involved in the study were not blinded before the experiment or during results assessment.

Reporting for specific materials, systems and methods

We require information from authors about some types of materials, experimental systems and methods used in many studies. Here, indicate whether each material, system or method listed is relevant to your study. If you are not sure if a list item applies to your research, read the appropriate section before selecting a response.

Materials & experimental systems

n/a	Involved in the study
☐	☒ Antibodies
☐	☒ Eukaryotic cell lines
☒	☐ Palaeontology and archaeology
☐	☒ Animals and other organisms
☒	☐ Clinical data
☒	☐ Dual use research of concern
☒	☐ Plants

Methods

n/a	Involved in the study
☒	☐ ChIP-seq
☐	☒ Flow cytometry
☒	☐ MRI-based neuroimaging

Antibodies

Antibodies used

Item Clones Catalog Number Dilution/ Concentration
CD3ε 145-2C11 100320, 100318, 100304 1:300
CD4 RM4-5 100528, 100524 1:300
CD8a/CD8 53-6.7 100722, 100744, 100720 1:300
B220 RA3-6B2 103224, 103222 1:300
Gr-1 RB6-8C5 108424, 108422, 108412, 108404 1:300
Mac-1 M1/70 101224, 101212, 101222 1:300
Ter119 TER-119 116222, 116224, 116220, 116204 1:300
CD19 6D5 115503 1:300
NK1-1 PK136 108724, 108722, 108741, 108704 1:300
TCRγδ GL3 118404 1:300
Sca-1 D7 108118, 108126, 108130 1:100
c-Kit 2B8 105826, 105812 1:100
CD34 RAM34 119316, 119304 1:50
CD150 (SLAM) TC15-12F12.2 115912, 115908, 115932 1:100
CD48 HM48-1 103032, 103432 1:100
CD135/Flt3 A2F10 135312, 135308 1:100
CD127/IL7Ra A7R34 135012 1:50
CD45.1 A20 110728, 110724, 110732, 110712, 110738 1:200
CD45.2 104 109812, 109832 1:200
CD25 PC61 102032 1:300
CD44 IM7 103030 1:300
CD16/32 93 101330 1:100
CD62L MEL-14 104432 1:300
H-2Kd SF1-1.1 116608 1:200
H-2Kb AF6-88.5.5.3 116612, 116624, 116605 1:200
CCR7 4B12 120112 1:100
CCR9 CW-1.2 125805 1:100
PSGL1 2PH1 137408 1:100
UEA-1 Lectin FL-1061 1:100
6C3 6C3 123408 1:100
EPCAM G8.8 118228 1:100
PDGFRa APA5 135912 1:100
MHC-II M5/114.15.2 107614 1:200
CD31 MEC13.3 102420 1:100
Ly6D 49-H4 138603 1:100
CD45 HI30 304028, 368510, 304012 1:200
CD14 M5E2 612902 1:200
CD15 W6D3 301918 1:200
CD56 B159, HCD56 757178, 318317 1:200
CD235a HI264 349116 1:200
CD1a HI149 300110 1:100
CD19 HIB19 302206 1:200
CD3 OKT3 317334 1:200

CD4 OKT4 317414 1:200
CD8 SK1 344712 1:200
CD11b ICRF44 301322 1:200
CD20 2H7 302312 1:200
CD5 UCHT2 300620, 300622 1:200
CD7 CD7-6B7 343104, 343114 1:100
CD38 HIT2 303532, 303506 1:100
CD10 HI10a 312212 1:100
CD45RA HI100 740298, 304112 1:100
CD90 5E10 328122 1:50
CD49F GoH3 313616 1:100
CD123 6H6 751841 1:100
CD117 104D2 313204 1:100
CD34 581 343620 1:100
Streptavidin 3A20.2 410501 1:300
ZBTB1 26287-1-AP 1:300
ZBTB1 C47476-AF647 1:50
Beta-actin N/A 1:10,000
Mouse FCR block 130-092-575 1:100
Human FCR block 130-059-901 1:100
Propidium iodide N/A 1:5000
7-AAD N/A 1:200
Zombie Aqua™ Fixable Viability Kit N/A 1:200
CD117 magnetic microbeads N/A variable

Validation

All antibodies are commercially available and their use has been validated by previous literature available through our group or other laboratories.

Eukaryotic cell lines

Policy information about cell lines and Sex and Gender in Research

Cell line source(s)

For lymphoid progenitor generation in vitro, we used the S17 stromal cells, obtained from K. Dorshkind (UCLA). To support in vitro T-cell differentiation, we used the MS5-mDLL4 or MS5-hDLL4 stromal cells, which obtained from Dr. Gay Crooks (UCLA).

Authentication

All cell lines have been previously validated in literature through other laboratories.

Mycoplasma contamination

All cell lines were tested for mycoplasma (and results are negative) prior to use.

Commonly misidentified lines
(See ICLAC register)

Misidentified lines is non applicable

Animals and other research organisms

Policy information about studies involving animals; ARRIVE guidelines recommended for reporting animal research, and Sex and Gender in Research

Laboratory animals

Young female mice (6-10 weeks of age): C57BL/6J (CD45.2/H-2Kb, JAX#000664), B6.SJL-PtprcaPepcb/BoyJ (CD45.1, JAX# 002014), and B6(C)-Gt (ROSA)26Sorem1.1(CAG-cas9*-EGFP) Rsky/J (Rosa26Cas9 KI, JAX#028555) mice were purchased from The Jackson Laboratory (JAX). CD45.1 (Stem)-CD45.2 chimeric female mice were obtained from Dr. Joseph Sun. Aged C57BL/6 (CD45.2/H-2Kb) mice (23-24 months of age) were obtained from National Institute of Aging (Baltimore, MD). Aged female mice (ranging between 18-24 months of age) correlate with humans ranging between 56-69 years of age. For recipients, we either used young (6-10 weeks of age) BALB/cJ (H-2Kd, JAX#000651) or generated middle-aged BALB/cJ mice (ranging between 14-16 months of age) by initially purchasing young BALB/cJ (H-2Kd) mice from JAX and subsequently allowing them to age under controlled conditions in our facility. Middle-aged mice (ranging between 14-16 months of age) correlate with humans ranging between 40-60 years of age. For consistency across all our transplantation experiments only female recipient mice were used. RAG2-EGFP-CD45.1 chimeric female mice were generated by crossing FVB-Tg (RAG2-EGFP)1Mnz/J (JAX# 005688) and B6.SJL-PtprcaPepcb/BoyJ (CD45.1, JAX# 002014). Young TCR-OT-1 (C57BL/6-Tg (TcraTcrb)1100Mjb/J, JAX#003831) transgenic female mice were obtained from Dr. Andrea Schietinger.

Wild animals

No wild animals were used in this study

Reporting on sex

All experiments were done on female mice hence gender was not taken into consideration

Field-collected samples

No field collected samples were used in this study

Ethics oversight

All the animal experiments were approved, and mice were euthanized with CO2 gas inhalation under an MSKCC Institutional Animal Care and Use Committee-approved protocol.

Note that full information on the approval of the study protocol must also be provided in the manuscript.

Plants

Seed stocks: n/a

Novel plant genotypes: n/a

Authentication: n/a

Flow Cytometry

Plots

Confirm that:

- [x] The axis labels state the marker and fluorochrome used (e.g. CD4-FITC).
- [x] The axis scales are clearly visible. Include numbers along axes only for bottom left plot of group (a 'group' is an analysis of identical markers).
- [x] All plots are contour plots with outliers or pseudocolor plots.
- [x] A numerical value for number of cells or percentage (with statistics) is provided.

Methodology

Sample preparation

In vivo analysis
PB Chimerism:Peripheral blood samples were collected in 50 mM EDTA solution (Thermo Fisher Scientific) via retro-orbital sinus bleeds. Thereafter PB was incubated with red blood cell lysis buffer (ACK lysis buffer, Thermo Fisher Scientific) for 8 minutes and then washed twice with PBS/2.5% fetal bovine serum. Cells were resuspended and then stained in PBS/2.5% fetal calf serum
BM chimerism:To calculate bone marrow cellularity and frequency of donor-derived hematopoietic precursors, two femurs and two tibias of primary recipient (BALB/cJ) were flushed into FACS buffer at 8- or 20-weeks post-transplant. Collected cells were then incubated with red blood cell lysis buffer (ACK lysis buffer, Thermo Fisher Scientific) for 8 minutes and then washed twice with PBS/2.5% FBS. Cells were resuspended and then stained in PBS/2.5% fetal calf serum.
Thymus processing: Briefly, thymi were mechanically dissociated into ca. 2 mm pieces. Tissue pieces were incubated with a digestion buffer (RPMI, 10% FCS, 62.5 um/mL liberase TM, 0.4 mg/ml DNase I) twice for 30 min at 37 C. Between incubation steps, supernatant containing dissociated cells was transferred to 50 mL conical tubes equipped with 100 um filter.
For thymic immunophenotypic analysis, cells were first incubated with Fc block solution (anti-CD16/CD32 antibody) for 10 min on ice. Solution was discarded and cells were stained in PBS/2.5% fetal calf serum.
Spleen analysis: spleens at 8- or 20-weeks post-transplant were excised and crushed, filtered through a 70uM strainer (pluriSelect), intermittently washed with PBS/2.5% fetal bovine serum and collected in a 50ml conical tube. Subsequently, spleen pellet was incubated in red blood cell lysis buffer (ACK lysis buffer, Thermo Fisher Scientific) for 8 minutes and then washed twice with PBS/2.5% fetal bovine serum. Prior to staining, an aliquot was set aside for counts with the Nexcelom Cellometer K2. For spleen immunophenotypic analysis, cells were first incubated with Fc block solution (anti-CD16/CD32 antibody) for 10 min on ice. Solution was discarded and stained in PBS/2.5% fetal calf serum
In vitro analysis
Cocultures were transferred through 40 μm filter to remove the stromal cells. All the cells in each well were harvested and analyzed by flow cytometry. The antibodies used to read the lineage outputs in this assay were anti-human CD45-BV480, anti-human CD14-BUV805, anti-human CD15-BV421, anti-human CD19-FITC, anti-human CD56-BUV496, and anti-human CD235a-PE-Cy7.

Instrument

Cells were analyzed using a FACS LSR II UV and LSR Fortessa X50 (BD Biosciences). Cells were sorted using Aria II or Aria IV cell sorter (BD Biosciences).

Software

Flow cytometry data were acquired using FACSDiva software (version 9.0, BD Biosciences) and analyzed using FlowJo software (version 10.8.2). Compensation was performed using single-stained controls, and gates were established using fluorescence minus one (FMO) controls, or isotype controls for intracellular flow detection.

Cell population abundance

For all experiments (transplantation and in vitro assays) in which Kithi, Kitlo or Kit HSCs were purified, they were double sorted to ensure >95% purity.

Gating strategy

We have provided a gating strategy to identify respective populations identified in our study in our supplementary figures.

- [x] Tick this box to confirm that a figure exemplifying the gating strategy is provided in the Supplementary Information.
