# Supplementary material for: Kitlo hematopoietic stem cells exhibit distinct lymphoid-primed chromatin landscapes that enhance thymic reconstitution
Source: Nat Commun. 2025 Jul 4;16:6170. doi: 10.1038/s41467-025-61125-1 (PMC12227609; doi:10.1038/s41467-025-61125-1)
Supplement: Supplementary file 11 — Transparent Peer Review file [file 41467_2025_61125_MOESM11_ESM.pdf]

nature portfolio

Peer Review File

Ultrapure and efficient electroluminescence in alkali metal doped inorganic perovskite quantum wires arrays

Corresponding Author: Professor Zhiyong Fan

Parts of this Peer Review File have been redacted as indicated to remove third-party material.

This file contains all reviewer reports in order by version, followed by all author rebuttals in order by version.

Version 0:

Reviewer comments:

Reviewer #1

(Remarks to the Author)
In this paper, Cao et al. reported the study of Rb and K ion-doped perovskite nanowire light-emitting diodes. They found that Rb and K ions could enter the lattice, causing lattice contraction, defect passivation, and improving device performance. Although the authors reported relatively high device efficiency, the scientific rigor of this paper is lacking. the authors claimed that the understanding of the principles of Rb and K doping in the perovskite field is insufficient. However, the characterization techniques they used and the results they obtained do not differ much from what is already known in the community. In brief, the authors believed they demonstrated that Rb and K could enter the lattice, which differs from previous reports in the literature. However, many reports indicate that at low doping levels, they can enter the lattice, but as the concentration increases, they tend to form aggregates (e.g. Nature, 2018, 555, 497–501; J. Am. Chem. Soc. 2017, 139, 14173; Adv. Optical Mater.2019, 7, 1901440). Besides, the authors characterization of the perovskite nanowires shows no difference from what they had previously reported (e.g. Nature Communications 14, 4611 (2023)). I also notice some technical issues in this paper. Therefore, I believe this paper is not suitable for publication in Nature Communications."

Here some technical concerns:

1. The authors claimed they prepared “quantum wires”. However, the emission for the perovskite without Cl is still in green region (peaking at 510~520 nm), suggesting no or very weak exciton confinement.
2. This is also contradicted with their temperature dependent PL measurements showing high exciton binding energy over 200 meV. The authors contribute to the large exciton binding energies to the small crystal size and strong quantum confinement (line 213). As in Q1, there is no distinct bandgap shift.
3. The authors should measure fluence dependent PLQY to evaluate the exciton recombination dynamics, if they believe the exciton binding energies are high.
4. The authors used DFT calculations to propose that the presence of AAO decreases the formation energies of Rb and K doped perovskites. And they also find a shrink of crystal lattice from XRD. However, If the doping of Rb and K is really facilitated by AAO, I believe Rb and K doping alone can effective tune the bandgap and thus the addition of Cl is not necessary.
5. The authors conclude that the Rb and K ions are not concentrated at the grain boundary. Such a result is simply indicated by the absence of other diffraction features from XRD and GIWAXS. However, the Rb and K salts (such as $RbPbX_3$, K_2PbX_4, and KX) can be amorphous.
6. As to the determination of Urbach energy, the authors claim largely reduced values from more than ~60 meV to around 20 meV with Rb and K addition. However, I notice that the absorption edge for $CsPb(BrCl)_3$ (e.g. Fig. S14a, 68.5 eV) is much more steeper compared to those with Rb/K (e.g. Fig. S14e, 23.8 eV). It is clearly incorrect.
7. There are clear steps for the absorption edge of $RbKCsPbBr_3$ in Fig S14f. Why
8. The authors believe that the PL lifetime is prolonged, suggesting a reduced trap-assisted non-radiative recombination. However, there is no clear difference in table S2 (7~9ns for the averaged lifetime).
9. Determination of shelf lifetime in dry air is completely meaningless (fig. 3)
10. The authors find that the addition of Rb/K leads to smaller FWHM. But the interpretation of these data is not solid. Perhaps it is more related electron phonon coupling. In addition, the results are similar to a recent report suggesting that the alkalic cations can homogenize Br/Cl distribution and thus improve the homogeneity and reduced the FWHM (Matter 2024,

7, 1054-1070).
11. The authors claimed that the Rb/K addition did not generate interstitial sites, suggested by TEM. However, it is not easy to visualize capture the interstitials in perovskites by HRTEM.
12. There is a typo in the caption of Figure S2. It should be Cs_4PbBr_6 not Cs_2PbBr_6.

Reviewer #2

(Remarks to the Author)
The manuscript describes the results of alkali metal doping studies of metal halide perovskites towards ultrapure and efficient light emitting diodes. The main founding presented in the manuscript is demonstration of the highly pure and efficient electroluminescence.
The results are very well presented. The conclusions are very much supported by the data. I recommend the manuscript for publication after minor revision.
(1) Beside the emission bandwidth, the emission peak wavelenths are also important to wide-color-gamut display applications, maybe 478nm, 532nm, 638nm for the blue, green and red ones.
(2) The operating half lifetimes are relatively low, especially for the blue ones. Could authors comment on this point.
(3) Why choose HAT TAPC as hole transporting layers instead of Pedots:PSS
(4) The open voltages of these leds are relatively high. Could authors comment on this point.

Reviewer #3

(Remarks to the Author)
In this article, Fan and co-workers report efficient sky blue (476 nm, 485 nm, and 490 nm) and green perovskite LEDs (512 nm) using nanopatterned alumina templates. Their green devices showed a remarkable EQE of ~30%. Using DFT, the authors demonstrate that the Al-Br and Pb-O bonds at the interface are responsible for reduced defects and enhanced PLQYs. At the same time, the authors employ Rb^+, and K^+ dopant ions to fabricate inorganic perovskite layers in patterned templates. Overall, this work is interesting, and the authors performed numerous studies to analyze the perovskite layers and consequently constructed efficient devices. However, I have several concerns that are listed below, which need to be addressed before publication in Nature Communications.

1) First, it is unclear where the enhancement in EQE is coming from. Is it due to the templated structure leading to an improved optical coupling or due to the doping? I am curious to see how undoped devices behave in the templated structure. The authors provide no convincing evidence that doping benefits the device performance.

2) Also, the authors used XRD peak shift to support the substitutional doping of Rb^+ and K^+, like many other reports, but they claim that this is unambiguous evidence of substitutional doping. This evidence is not enough to support their bold claims. Also, why do you see a peak shift in those planes, where the reflections are from octahedra and A-site has no major role?. Moreover Rb and Cs have similar ionic radii. The shift in the peaks, sometimes, is also attributed to the “height effect,” where it is possible that the resultant templated structure may have different sample heights.

3) The primary blue 476 nm LEDs show a remarkable max EQE of ~17%, whereas their PLQY is only 66%. In addition, there is an insulating Al_2O_3 in the template. Despite these, the performance is very high. To me, this indicates that there was nearly no loss in the free carriers, and the majority of the injected carriers led to the emission. Can you explain what is responsible for this?, are these PLQYs measured in templated structures?. Generally, mixed halides tend to suffer more from defects, which are detrimental in device EQE, but authors seem to have no such issues.

4) Do you see a shift the EL peak with time when mixed halides are used in a device?

Other minor points
1) The PLQYs and EQEs are reported as 4 sig figs, which is not appropriate unless the authors perform a statistical analysis to prove that all the numbers are significant. So, I encourage the authors to round it to 2 sigfigs.
2) The figure legends are hard to read due to the font size and the color in Figures 3 and 4

Version 1:

Reviewer comments:

Reviewer #1

(Remarks to the Author)
I appreciate the efforts made by the authors, but unfortunately, I am not convinced.

I agree that utilizing identical techniques to those employed by others does not inherently indicate a lack of novelty. However, my primary concern is that the paper's conclusion—that Rb and K can replace Cs—is not novel. Furthermore, the authors assert that 20% and 13.3% of Rb and K have been incorporated into the crystal lattice, yet the photoluminescence (PL) and electroluminescence (EL) exhibit minimal changes (e.g., perovskite emission remains at 512 nm).This is certainly unreasonable. As a large number of doping results in lattice construction and significantly enlarged bandgap. Here is more

literature for the references (e.g. Nanoscale, 2018,10, 3429-3437; Nature Communications 2019, 10, 1868), where 20% Rb incorporation results in a big shift in the bandgap.

I would expect to observe a continuously enlarged bandgap of perovskites with varying Rb/K concentrations without the aid of Cl ions. It is possible that only a small fraction of Rb or K ions are successfully integrated into the lattice at high doping levels, while the majority form aggregates. The XRD results are not particularly informative in this scenario, as aggregates may be amorphous and thus not easily detectable. Additionally, HRTEM is limited in its ability to provide comprehensive insights, as it examines only very small regions of the material. In my view, synchrotron-based nano X-ray fluorescence microscopy and solid-state NMR would be very helpful to detect whether Rb/K cations are uniformly distributed into perovskites or being aggregates, upon different Rb/K content. Besides, XPS may also help if the Rb/K doping ratio is indeed as high as what the authors claim. It is unclear why the authors did not include Pb, Rb, and K core-level spectra in Figure S9, as this data would be critical for supporting their claims, especially for those with high Rb/K contents.

In addition, the three papers I mentioned aimed to show that for different cases, including perovskite stoichiometry and K/Rb doping level, the role of these alkalic ions can be different.

For more responses:

1. The authors claimed that their perovskites are quantum wires, despite the emission of $CsPbBr_3$ being in the green spectrum. They attribute this to the confinement dimensionality being lower than that of quantum dots (QDs). While this rationale is correct, it raises a critical question: if the confinement is not sufficiently strong, is it appropriate to classify these materials as quantum wires? This issue is analogous to the distinction between nanocrystals and quantum dots. Additionally, the authors reference a Nature publication concerning the in-situ formation of perovskite QDs. In that study, the authors successfully fabricated quantum-confined $CsPbBr_3$ QDs exhibiting deep blue emission, which starkly contrasts with the current findings. The authors also cite their previous work; however, it does not make their argument to be correct. Regarding questions Q1 and Q2, I recommend that the authors consult the study by Annamaria's group (J. Am. Chem. Soc. 2014, 136 (51), pp. 17730–17733) for further insights and clarification.

2. As I said, if the perovskite samples in this paper are QWs and the binding energy is high, we should see a big difference compared to bulk in recombination dynamics. Fluence dependent PLQY or fluence dependent decay are necessary to extract the recombination constants.

3. For Q4, While the authors provide extensive discussion on Q4, they fail to address my concern. I would like to see a continuously shifted bandgap of AAO-CsPbBr3 with increasing the doping ratio of Rb or K. Demonstrating such a shift would serve as compelling evidence that the incorporation of Rb and K is facilitated by AAO. Additionally, including a comparison with an AAO-free sample would enhance the validity of this claim by clearly illustrating the role of AAO in the doping process.

4. For Q5, the resolution of EDS and its ability to distinguish different metallic element is not good enough. For instance, from the mapping of Rb, it seems that it is concentrated in top contact, but this is not true. Combining XRD and GIWAXS, I agree that K and Rb cations can enter the lattice. However, it does not imply that all the K and Rb ions can do that. The authors should give more evidence to demonstrate that the Rb and K are not concentrated somewhere, especially for high doping levels such as 20%.

5. For Figure S14, please unify the photon energy range on the horizontal axis.

6. For the PL lifetime, I still believe it is over-interpreted. There is no difference between 7 and 9 ns. Furthermore, considering that an increase in Rb/K or Cl doping typically leads to an enlarged exciton binding energy, one would expect a corresponding acceleration in recombination rates. This anticipated faster recombination contradicts the observed PL decay behavior. Therefore, the PL decay alone cannot be used for understanding the defect physics and recombination dynamics. Other tests including fluence-dependent PLQY/decay or TA to extract the dynamics are important.

7. For Q9, I do not believe a test for shelf stability in dry air (10-20% humidity) is meaningful. There are several reasons:
i) Although OLED emitter films exhibit instability in ambient air, this does not present a significant barrier to their commercialization. In practical applications, protective encapsulation effectively mitigates these stability issues.
ii) State-of-the-art perovskite solar cells, lots of which are also mixed halide perovskites, can be stable even in water. Basically, a test close to 85°C/85% relative humidity test for thousands of hours is required.
Besides, in our lab, the mixed halide perovskite emitters can be stored in dry air for several months without any negative effect on their optical properties. With all respect, I do not think anyone cares about that.

Reviewer #2

(Remarks to the Author)
Fan et al. have solved my previos concerns. Published as is.

Reviewer #3

(Remarks to the Author)
I am fairly satisfied with the authors response to my comments. I recommend this work for publication in Nature Communication. However, I suggest authors to revise their figures and replace with high quality and legible ones. The lables, and legens are hard to read.

Version 2:

Reviewer comments:

Reviewer #1

(Remarks to the Author)
The author believes that what they are dealing with is a quantum wire, but I still believe this is incorrect, as evidenced by the author's own spectral characterization. Unfortunately, I cannot recommend the acceptance of this manuscript.
Firstly, if it was truly a quantum wire, the first-order exciton radiation recombination would be dominate, rather than bimolecular radiation recombination. However, from the nature of PL decay curve (figure R10, in line with double exponential fitting), it is more likely that bimolecular recombination is the dominant recombination pathway.
Secondly, From the author's characterization, the first-order recombination (k1) rate appears to have slowed down compared to the bulk perovskites, which is not reasonable. There is no doubt that quantum wires are of faster first-order recombination rates, due to the contribution from exciton recombination.
Additionally, for quantum materials with quantum or dielectric confinement, Auger recombination (k3) is typically much greater than in bulk materials. This is determined by the photophysical nature, that is, the enhanced exciton-exciton interactions.
Besides, I find it hard to believe that Auger recombination in perovskites could reach a level of 10^{-30} cm^{-6} s^{-1}, as this is slower than what is observed in current perovskite photovoltaics or single-crystal materials, making it, in my view, implausible.
I believe the author is, on the one hand, parroting others, and on the other hand, while I acknowledge that some of their previous articles demonstrated clear evidence of confinement, such confinement is absent here, and thus it does not meet the definition of a quantum wire. The author likes to use TEM to prove their nanostructures, but as I mentioned before, TEM is not comprehensive. From some of the localized region, it appears to be a nanowire or with nanostructure. However, if viewed globally, the perovskite material observed from this angle may extend over a much larger area, allowing charge carriers to move across a significantly broader region. Anyway, the results provided by optical methods are more comprehensive and accurate.

Version 3:

Reviewer comments:

Reviewer #1

(Remarks to the Author)
I appreciate the authors' efforts in investigating carrier dynamics and acknowledge that their conclusion—excitonic recombination dominance—could support the achievement of quantum wires. Although I remain somewhat puzzled by how the authors arrived at results that appear to completely contradict their previous findings within such a short timeframe and using identical technology, I see no reason to reject this manuscript.
However, I remain unconvinced by the shelf lifetime data (Fig. 3e), as I said it is not meaningful. I guess the authors are not willing to show their short operational lifetime in their main context, and thus use the shelf lifetime instead. Given the high impact of Nature Communications, I am concerned that the current presentation of these results may potentially mislead the perovskite research community.

Reviewer #3

(Remarks to the Author)
I think authors demonstrated extensive studies to prove that they are synthesizing perovskite quantum wires. Their absorption data both steady-state and transient now shows convincing evidence of nanowire formation. I strongly encourage the authors to replace both Absorption and TA data in the SI with high resolution figures. Overall, I am satisfied with authors response and I recommend this work for publication in Nat comm.

Open Access This Peer Review File is licensed under a Creative Commons Attribution 4.0 International License, which permits use, sharing, adaptation, distribution and reproduction in any medium or format, as long as you give appropriate credit to the original author(s) and the source, provide a link to the Creative Commons license, and indicate if changes were made.
In cases where reviewers are anonymous, credit should be given to 'Anonymous Referee' and the source.
The images or other third party material in this Peer Review File are included in the article's Creative Commons license, unless indicated otherwise in a credit line to the material. If material is not included in the article's Creative Commons license and your intended use is not permitted by statutory regulation or exceeds the permitted use, you will need to obtain permission directly from the copyright holder.
To view a copy of this license, visit https://creativecommons.org/licenses/by/4.0/

Response to the Reviewers' Comments

[Nature Communications NCOMMS-24-55954-T]: Ultrapure and efficient electroluminescence in alkali metal doped inorganic perovskite quantum wires arrays

We would like to thank the reviewers and the editors for their valuable comments on our work. Such productive feedback from the experts in this field helped us to improve our manuscript. Here we provide a point-by-point response below to the reviewers' questions. We also revised our manuscript to comply with the reviewers' comments and have highlighted the revised parts in red.

Reviewer #1 (Remarks to the Author):

In this paper, Cao et al. reported the study of Rb and K ion-doped perovskite nanowire light-emitting diodes. They found that Rb and K ions could enter the lattice, causing lattice contraction, defect passivation, and improving device performance. Although the authors reported relatively high device efficiency, the scientific rigor of this paper is lacking. the authors claimed that the understanding of the principles of Rb and K doping in the perovskite field is insufficient. However, the characterization techniques they used and the results they obtained do not differ much from what is already known in the community. In brief, the authors believed they demonstrated that Rb and K could enter the lattice, which differs from previous reports in the literature. However, many reports indicate that at low doping levels, they can enter the lattice, but as the concentration increases, they tend to form aggregates (e.g. Nature, 2018, 555, 497–501; J. Am. Chem. Soc. 2017, 139, 14173; Adv. Optical Mater.2019, 7, 1901440). Besides, the authors characterization of the perovskite nanowires shows no difference from what they had previously reported (e.g. Nature Communications 14, 4611 (2023)). I also notice some technical issues in this paper. Therefore, I believe this paper is not suitable for publication in Nature Communications."

Response: We thank the reviewer for pointing out the ambiguity in our discussion of these

experimental and theoretical results, which have helped us to prepare a revised paper with improved scientific depth and clarity. We hope the reviewer can find our substantially revised paper suitable for publication.

The reviewer mentioned in the comments that the characterization techniques we used and the results we collected have no difference from what's already known in the research community. Besides, the characterization techniques we conducted show no difference from our previous works. With all due respect, we think these comments are prejudiced. For example, a work (Joule, Volume 7, Issue 12, 2905 - 2922) with similar topic published in Joule, providing unambiguous evidence for the formation of tetragonal β-(DMA, Cs)PbI_3 by substituting the small ionic radius Cs^+ with the large organic cation DMA^+. Before the publication of this work, some assert that DMA^+ helps to form an intermediate phase that facilitates the formation of black CsPbI3 without being incorporated into the perovskite structure while others argue that DMA^+ can be incorporated into perovskite structure. The characterization techniques they used include ultra-low dose transmission electron microscopy (ULD-TEM), high-angle annular dark-field scanning transmission electron microscopy (HAADF-STEM), XRD, elemental analysis by nuclear magnetic resonance (NMR), time-of-flight secondary-ion mass spectrometry (ToF-SIMS), and thermogravimetric (TG) analysis, which are very common and widely used in the research community. In addition, the results they obtained are also similar to other reports (ACS Energy Lett. 2020, 5, 1, 263–270, Angew. Chem. Int. Ed. 2019, 58, 16691). Moreover, many excellent works published in top journals (Nature 612, 679–684 (2022), Nat. Photon. 18, 363–370 (2024), Nature 633, 344–350 (2024)) are using common and well-established characterization techniques. Thus, we believe that it's unreasonable to consider the use of well-established characterization techniques as a basis for rejection.

The reviewer listed three papers to support the argument that many reports indicate that Rb^+ and K^+ can enter the lattice at low doping levels, but they tend to form aggregates as the concentration increases. However, the conclusion of the first two papers differs from the reviewer's comment. In Nature, 2018, 555, 497–501, the conclusion is "Collectively, these results indicate the formation of potassium halide (particularly bromide-rich) passivation layers

decorating the surfaces, where the potassium is NOT incorporated into the perovskite lattice.”. As for J. Am. Chem. Soc. 2017, 139, 14173, the conclusions are “Rubidium, on the other hand, does NOT form a solid alloy with FA in any of the studied compositions. Rather, it separates into a mixture of rubidium-rich phases ($RbPbI_3$ mixed cesium–rubidium lead iodides, mixture of rubidium halides, various rubidium lead bromides, depending on the exact composition).” and “We have also found that potassium, which has a size similar to rubidium, is NOT incorporated into the $MAPbI_3$ lattice.”. Similar to the DMA^+ mentioned above, controversy remains regarding the specific location of Rb^+ and K^+ after doping. Even though some reports suggest that Rb^+ and K^+ can replace Cs^+ at A-site, the majority still believes the opposite. The majority claims that they tend to occupy the interstitial site in the lattice or form Rb^+-rich and K^+-rich compounds at the grain boundary. In contrast, we demonstrated the possibility of Rb+ and K+ can substitute A-site Cs+ in the perovskite lattice of our confined quantum wires with sufficient evidence. Furthermore, the doping concentration of Rb^+ and K^+ are quite high in our case, which are 20% and 13.3% respectively. However, Rb^+ and K^+ still can substitute Cs^+ at A-site.

Actually, the characterization techniques, which were used to determine the specific location of Rb^+ and K^+, of cited references in the introduction (Revised manuscript line 72-74) include XRD, GIWAXS, TEM, HRTEM, HAADF STEM and so forth. However, the majority of cited references only utilized one or two of the listed characterization techniques, a practice we consider to be inadequate. Therefore, we conducted atomically resolved low-dose HRTEM to reveal the atomic structure of Rb^+ and K^+ doped PeQWs and DFT calculation to obtain the formation energy of Rb^+ and K^+ replacing Cs^+ with and without Al_2O_3. Combining with XRD and GIWAXS results, we are confident to conclude that Rb^+ and K^+ can substitute Cs^+ at A-site in our case.

Here some technical concerns:

1. The authors claimed they prepared “quantum wires”. However, the emission for the perovskite without Cl is still in green region (peaking at 510~520 nm), suggesting no or very weak exciton confinement.

Response: We appreciate the reviewer bringing up this important point. The quantum confinement effect in our PeQWs is slightly less pronounced than that in perovskite QDs. This is due to perovskite QDs exhibiting a three-dimensional quantum confinement effect, whereas our PeQWs only demonstrate a two-dimensional quantum confinement effect. The exciton Bohr diameter of $CsPbBr_3$ is around 7 nm. The diameter of our $CsPbBr_3$ QWs is ~6.4 nm which is slightly smaller than the exciton Bohr diameter of $CsPbBr_3$, meaning that our PeQWs are in the quantum-confinement domain. Due to the diameter of our PeQWs is closed to the exciton Bohr diameter, the quantum size effect is expected to be less pronounced. In a published report (Nature 612, 679–684 (2022)), the authors reported the direct synthesis-on-substrate of films of suitably coupled, monodispersed, ultrasmall perovskite QDs. By changing the ligand concentration, they successfully obtained perovskite QDs with different diameters (Figure R1). Similarly, perovskite QDs with 6.4 nm diameter emit green light peaking at ~515 nm.

[FIGURE REDACTED]

Figure R1. Relationship among ligand concentration, the peak wavelength of QD films and the size of the QDs. (Nature 612, 679–684 (2022))

In our previous report (Nat. Photon 2022, 16, 284), we also demonstrated $MAPbBr_3$ NWs with different diameters. The PL peak gradually shifts from 530 nm to 517 nm as the diameter reduces from 250 nm to 6.4 nm. In another report (Sci. Adv. 2017;3: e1700738), the authors compared the PL peak of $CsPbBr_3$ QWs and bulk $CsPbBr_3$. The PL peak shifts from ~530 nm to 512 nm. Thus, we believe that it's reasonable that the emission of our $CsPbBr_3$ QWs is still in the green region.

[FIGURE REDACTED]

Figure R2. a, PLQY and PL peak position of NW arrays with diameters decreasing from the bulk (280.0 nm) to quantum (6.4 nm) regime. (Nat. Photon 2022, 16, 284) b, PL of ncMAPb3 grown in npAAO (solid red line), blue-shifted by 62 nm. ncMAPbBr3 (solid green line) and ncCsPbBr3 (solid cyan line) are shifted by 14 and 16 nm, respectively. Dashed lines show bulk film PL. (Sci. Adv. 2017;3: e1700738)

2. This is also contradicted with their temperature dependent PL measurements showing high exciton binding energy over 200 meV. The authors contribute to the large exciton binding energies to the small crystal size and strong quantum confinement (line 213). As in Q1, there is no distinct bandgap shift.

Response: We thank the reviewer for the valuable comments. Typically, significant quantum confinement results in strong excitonic confinement, leading to a large exciton binding energy. To the contrary, it may not necessarily hold true. A clear example was demonstrated by Prof. Tae-Woo Lee and co-works in the Nature paper entitled "Ultra-bright, efficient and stable perovskite light-emitting diodes" (Nature 611, 688–694 (2022)). They used BPA treatment to shrink the perovskite grain size from 205 ± 97 nm to 10± 2 nm. However, the emission peak of BPA treated perovskite film only blue-shifted a few nanometers. Besides, 10± 2 nm grain size is still beyond the exciton Bohr diameter of $FAPbBr_3$. By conducting dependent PL measurements, the exciton binding energy was increased from 90 meV to 220 meV for perovskite film with and without BPA treatment. They attributed the enhanced exciton binding

energy to the strong confinement effect from the grain size reduction. We believe that it's logical to conclude that our 6.4 nm PeQWs experience strong confinement effect since the crystal size of our PeQWs is much smaller than the BPA treated perovskite films.

There are many factors that will influence the exciton binding energy of perovskite. The exciton binding energy can be altered by the chemical composition, dimensionality, crystal structure, internal defects, and other properties of perovskite. The quantum confinement effect from the ultra-small diameter is only one of the reasons why our PeQWs have lager exciton binding energy. That's also why our PeQWs have high exciton binding energy over 150 meV in our previous report (Nature Communications 14, 4611 (2023)). After doping Rb^+ and K^+, the exciton binding energy was increased to over 200 meV. We believe that it's related to the reduced internal defects and the shrinkage of perovskite lattice. We have revised the manuscript accordingly.

The question related to the bandgap shift has been answered in Q1.

Revision: Page 10 line 212-215 in the revised manuscript.

3. The authors should measure fluence dependent PLQY to evaluate the exciton recombination dynamics, if they believe the exciton binding energies are high.

Response: We are grateful to the reviewer for the helpful advice. The most common and effective technique to evaluate the exciton recombination dynamics is transient absorption spectroscopy, which has been widely used in many published reports (Nat. Photonics 13, 418–424 (2019), *Nat Commun* 12, 361 (2021), Science 371, 1129-1133 (2021), Nature 612, 679–684 (2022), Nature 622, 493–498 (2023), Nat. Photon. 18, 425–431 (2024), Nat. Photon. 18, 363–370 (2024), Nature 631, 73–79 (2024)). And we have already given a clear explanation on the exciton recombination dynamics of our PeQWs in our previous report (ACS Nano 2022, 16, 5, 8388–8398). Therefore, we didn't intend to discuss more about this again in this paper. Typically, the exciton binding energy is obtained through temperature dependent PL

measurements (Nat Commun 12, 336 (2021), Nature 612, 679–684 (2022), Nature 611, 688–694 (2022), Nature 622, 493–498 (2023), Nature 631, 73–79 (2024)). Thus, we believe that the exciton binding energy extracted from temperature dependent PL measurements is solid. Unfortunately, we are unable to measure fluence-dependent PLQY at present because our equipment is not equipped with an intensity-tunable laser diode.

4. The authors used DFT calculations to propose that the presence of AAO decreases the formation energies of Rb and K doped perovskites. And they also find a shrink of crystal lattice from XRD. However, If the doping of Rb and K is really facilitated by AAO, I believe Rb and K doping alone can effectively tune the bandgap and thus the addition of Cl is not necessary.

Response: We appreciate the insightful comments. There are many approaches to tune the bandgap of perovskite, including dimensionality engineering and chemical composition engineering. In dimensionality engineering, two prevalent methods to manipulate the bandgap are reducing the crystal size to induce quantum size effect and introducing long-chain ligand to form qusi-2D or pure-2D perovskite (Nat. Nanotechnol. 15, 668–674 (2020), Nature 612, 679–684 (2022), Nature 622, 493–498 (2023), Nat. Photon. 18, 425–431 (2024)). As for chemical composition engineering, changing the anion or cation at A, B, X-site can also tune the bandgap (Nat Commun 10, 1868 (2019), Nature 618, 74–79 (2023), Adv. Mater. 2021, 33, 2101903, Adv. Optical Mater. 2020, 8, 2001073, J. Phys. Chem. Lett. 2021, 12, 6, 1747–1753, Nat Commun 12, 361 (2021)). Different approaches will result in diverse material properties and device performance. Indeed, only doping Rb^+ and K^+ without the addition of Cl can be a very interesting topic to study and we will explore this approach in the future. However, mixed-halide perovskite is also a very interesting category to study. And doping chlorine to perovskite to achieve blue emission is very common. Bromide-based perovskite can be entirely transformed into pure chloride-based perovskite. However, it's possible that the A-site substitution with Rb^+ and K^+ may not succeed under high doping concentration (> 50%), due to small tolerance factor (< 0.8) of $RbPbBr_3$ and $KPbBr_3$. Thus, we think that the addition of chlorine is necessary.

5. The authors conclude that the Rb and K ions are not concentrated at the grain boundary. Such a result is simply indicated by the absence of other diffraction features from XRD and GIWAXS. However, the Rb and K salts (such as $RbPbX_3$, $K2PbX_4$, and KX) can be amorphous.

Response: The reviewer is acknowledged for raising this interesting point. If amorphous Rb-rich and K-rich compounds concentrated at the grain boundary, we will observe distinct signal showing enrichment of Rb and K elements at a specific location in the EDS mapping images (ACS Energy Lett. 2018, 3, 11, 2671–2678). However, it was not the case for our EDS mapping images. Besides, we will observe Rb-rich and K-rich compounds at the boundary between PeQWs and Al_2O_3 in Figure 1 h-j, if they are enriched there. Combining XRD, GIWAXS, HRTEM and EDS mapping results, we conclude that Rb and K didn't concentrate at the grain boundary.

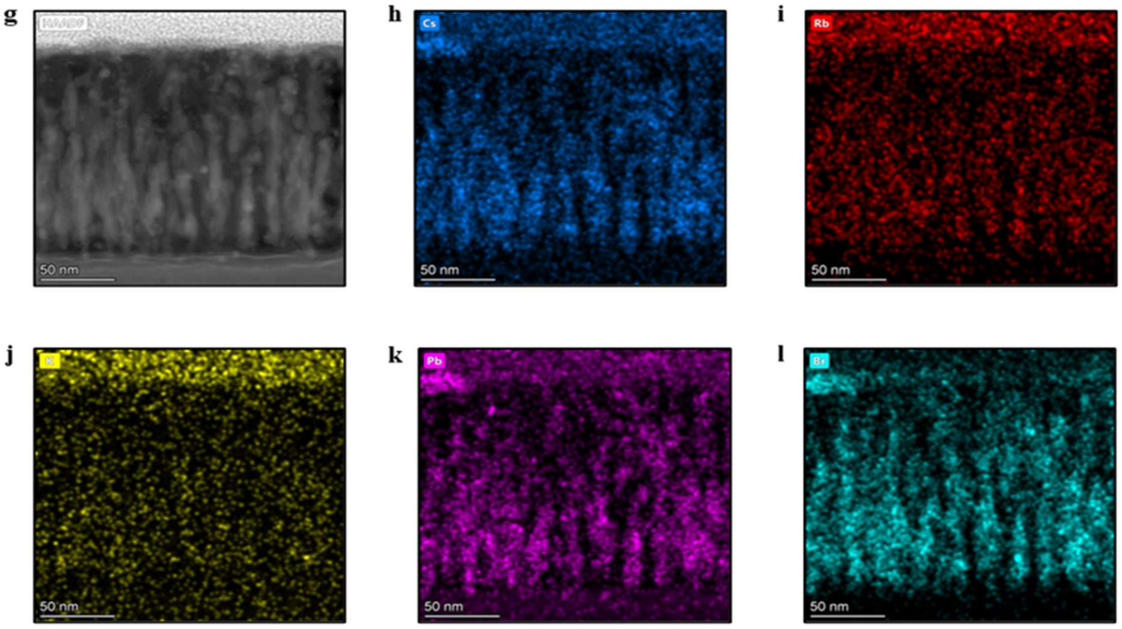

Supplementary Figure 3. g, Cross-sectional TEM images of Rb^+, K^+: $CsPbBr_3$ QWs. The corresponding EDS mapping images of h, Cs, i, Rb, j, K, k, Pb and l, Br elements.

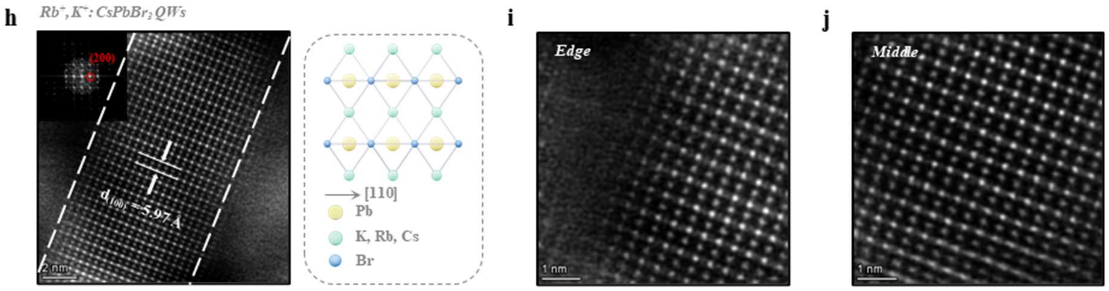

Figure 1. h, Atomically resolved low-dose HRTEM image of single Rb^+, K^+: CsPbBr3 QW with atomic structure model projected along <110> direction. Inset, fast Fourier transform diffractograms showing the cubic lattice structure. i, Zoom-in low-dose HRTEM image of perovskite lattice in the edge of Rb^+, K^+: CsPbBr3 QWs. j, Zoom-in low-dose HRTEM image of perovskite lattice in the middle of Rb^+, K^+: CsPbBr3 QWs.

6. As to the determination of Urbach energy, the authors claim largely reduced values from more than ~60 meV to around 20 meV with Rb and K addition. However, I notice that the absorption edge for $CsPb(BrCl)_3$ (e.g. Fig. S14a, 68.5 meV) is much more steeper compared to those with Rb/K (e.g. Fig. S14e, 23.8 meV). It is clearly incorrect.

Response: We appreciate the reviewer's careful observation. The absorption edge for pristine $CsPb(Br_{1-x}Cl_x)_3$ QWs appears steeper compared to those with Rb, K: $CsPb(Br_{1-x}Cl_x)_3$ QWs due to the fact that these two plots are presented on different scales. Actually, after doping Rb^+ and K^+, the absorption coefficient changed dramatically. If we plot them into one figure, you will notice that the absorption edge for Rb, K: $CsPb(Br_{1-x}Cl_x)_3$ QWs is steeper than those with pristine $CsPb(Br_{1-x}Cl_x)_3$ QWs.

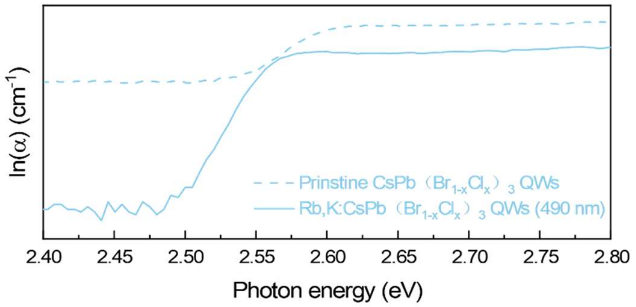

Figure R3. Absorption coefficient spectra for pristine $CsPb(Br_{1-x}Cl_x)_3$ QWs and Rb, K: $CsPb(Br_{1-x}Cl_x)_3$ QWs.

7. There are clear steps for the absorption edge of $RbKCsPbBr_3$ in Fig S14f. Why

Response: We are grateful to the reviewer's careful observation. We believe that there are some unknown problems with the previous sample, which leads to the abnormal result. We have measured more samples and found the steps disappeared. Below please find a representative result which we have updated in Fig S14f.

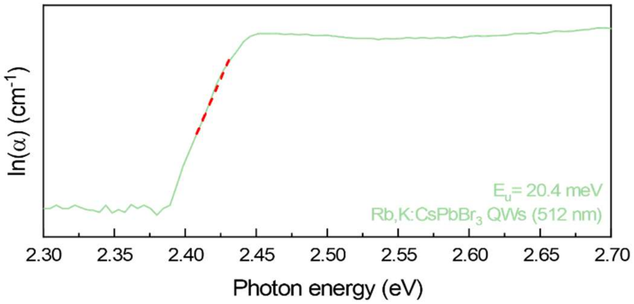

Figure R4. Absorption coefficient spectra for Rb, K: $CsPbBr_3$ QWs.

Revision: Supplementary Figure 14 in the revised supplementary information.

8. The authors believe that the PL lifetime is prolonged, suggesting a reduced trap-assisted non-radiative recombination. However, there is no clear difference in table S2 (7~9ns for the averaged lifetime).

Response: We thank the reviewer for pointing out this issue. We made several new batches of samples for time-resolved photoluminescence decay test. The representative results are plotted in Figure R5. The average PL lifetimes distribution of PeQWs is shown in Figure R6. We have to clarify that we compared pristine $CsPbBr_3$ QWs (513 nm) with Rb, K: $CsPbBr_3$ QWs (512 nm) and pristine $CsPb(Br_{1-x}Cl_x)_3$ QWs (486 nm) with Rb, K: $CsPb(Br_{1-x}Cl_x)_3$ QWs (490 nm).

We didn't increase the chloride concentration for pristine $CsPb(Br_{1-x}Cl_x)_3$ QWs because the average PL lifetimes of pristine $CsPb(Br_{1-x}Cl_x)_3$ QWs (486 nm) is already shorter than Rb, K: $CsPb(Br_{1-x}Cl_x)_3$ QWs (476 nm). As shown in Figure S4, the average PL lifetime for alkali metals doped PeQWs is prolonged compared with the pristine PeQWs. As we mentioned in the manuscript, the PL decay curves are fitted into two regions, with a short lifetime (τ_1) associated with trap-mediated nonradiative recombination, and a longer lifetime (τ_2) ascribed to radiative recombination. Therefore, the calculated average PL lifetime reflects a trade-off between trap-mediated nonradiative recombination rate and radiative recombination rate. We also summarized the τ_1 and τ_2 in the supplementary table 2. You can clearly notice that the τ_1 for alkali metals doped PeQWs is definitely longer than those for pristine PeQWs.

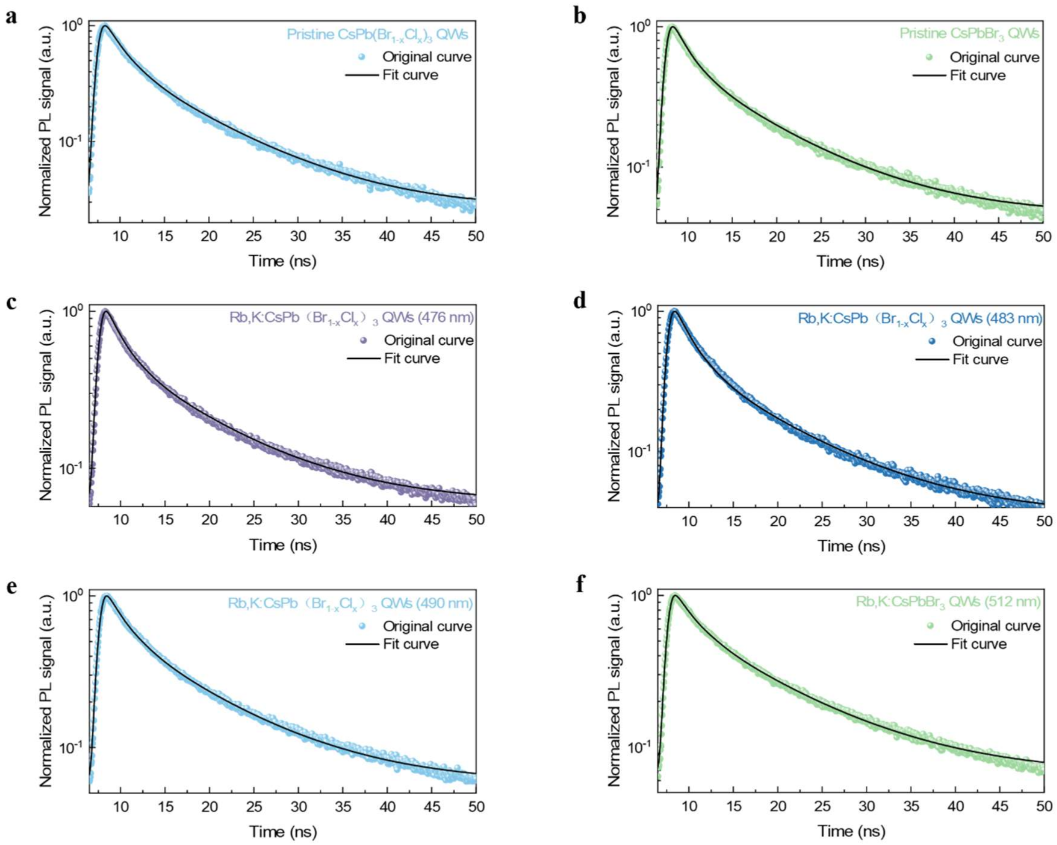

Figure R5. Normalized time-resolved photoluminescence decay curves for a, b, pristine PeQWs and c-f, alkali metals doped PeQWs.

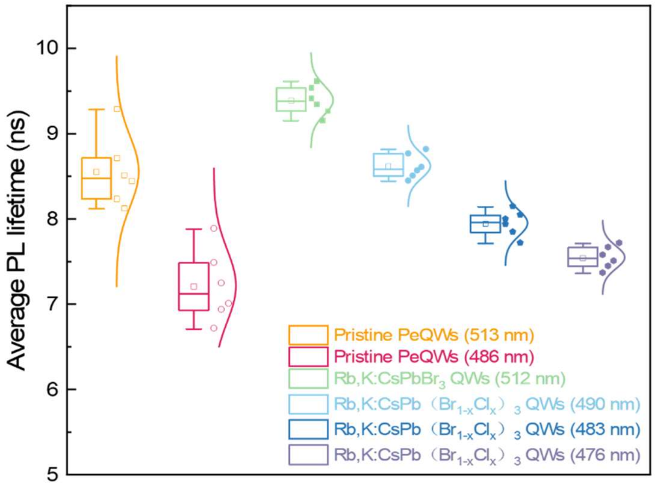

Figure R6. The average PL lifetimes distribution of PeQWs.

9. Determination of shelf lifetime in dry air is completely meaningless (fig. 3)

Response: We thank the reviewer for this critical comment. We fabricated new batch of samples for shelf lifetime in ambient condition with 24-28°C and 60-70% related humidity. As you can see from the figure below, Rb, K: $CsPbBr_3$ QWs (512 nm) and Rb, K: $CsPb(Br_{1-x}Cl_x)_3$ QWs (490 nm) can retain 100% of the initial PL intensity after 744 hours in this ambient condition. The PL intensity of Rb, K: $CsPb(Br_{1-x}Cl_x)_3$ QWs (483 nm) reduced to 99.5% of the initial PL intensity after 744 hours. However, it reduced to 98.2% for Rb, K: $CsPb(Br_{1-x}Cl_x)_3$ QWs (476 nm) after 744 hours. We attribute such a high environmental stability to the protection provided by our NAT against water and oxygen. We also believe that the shelf lifetime in dry air is meaningful, because most mixed-halide perovskites even can't survive in dry air without encapsulation (Journal of Luminescence 263 (2023) 120114).

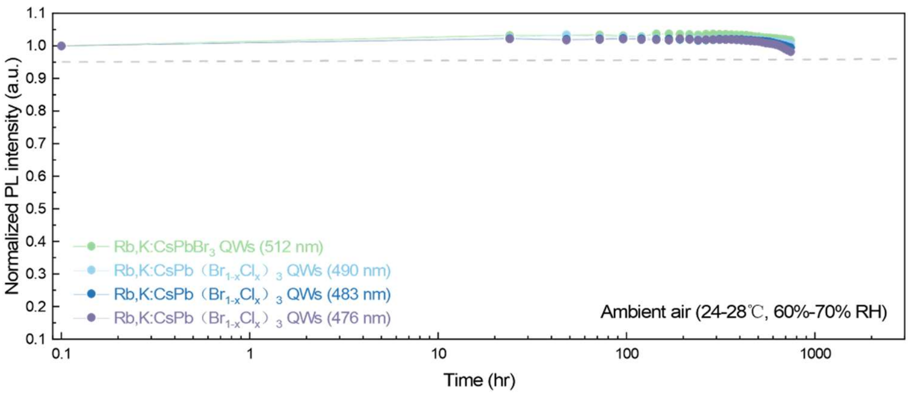

Figure R7. Environmental photoluminescence stability under ambient condition for alkali metal doped PeQWs.

10. The authors find that the addition of Rb/K leads to smaller FWHM. But the interpretation of these data is not solid. Perhaps it is more related electron phonon coupling. In addition, the results are similar to a recent report suggesting that the alkalic cations can homogenize Br/Cl distribution and thus improve the homogeneity and reduced the FWHM (Matter 2024, 7, 1054-1070).

Response: We appreciate the reviewer for the valuable comments and sharing the representative paper. Since our PeQWs are low-dimensional materials, the variation in diameter and interwire distance will lead to undesired optical properties mentioned in the manuscript if they are strongly coupled with each other. In another report (Adv. Mater. 2024, 36, 2404856), the authors demonstrated that large size distribution of QDs and strongly coupled QDs results in the inhomogeneous line broadening and redshift of emission. However, the interwire distance of our PeQWs is more than 10 nm, thus our PeQWs should not exhibit such a phenomenon. Furthermore, lower defect density will also contribute to a narrower emission linewidth. After the doping of Rb^+ and K^+, the phase purity and defect density of PeQWs is reduced. Therefore, we believe that our interpretation is solid.

Our interpretation is one case among all of the explanations for the narrower emission linewidth. We have studied the paper the reviewer listed while we were formulating our manuscript.

Nevertheless, we believe that it is imperative to explore an alternative explanation as previous studies have shown that alkalic cations can homogenize halide distribution, leading to a reduced emission linewidth. Since the reviewer mentioned this paper in the comment, we have incorporated the mentioned explanation into the revised manuscript

Revision: Page 10-11, line 227-233 in the revised manuscript.

11. The authors claimed that the Rb/K addition did not generate interstitial sites, suggested by TEM. However, it is not easy to visualize capture the interstitials in perovskites by HRTEM.

Response: We thank the reviewer for raising this concern. Indeed, it's not very easy to visually capture the interstitials in perovskite by HRTEM. Actually, we have attempted to obtain atomic-resolution EDS mapping of our PeQWs to check the specific location of each atom. Unfortunately, PeQWs decomposed rapidly during the scanning process, which is the case for most Cs-based perovskites. According to the previous reports (Adv. Mater. 2018, 30, 1707350, Adv. Optical Mater. 2020, 8, 2000742, Adv. Funct. Mater. 2022, 32, 2208077, J. Mater. Chem. C, 2024,12, 6395-6405), the XRD peak position for different planes will shift to a lower angle and the interplane distance of crystal lattice will increase if Rb^+ and K^+ occupy interstitial sites. However, it's not the case for our PeQWs. We also examined the profile of signal intensity at different positions in the PeQWs lattice. We found that there is no clear signal for atom occupying at the interstitial sites. Combining XRD, GIWAXS and HRTEM results, we conclude that Rb^+ and K^+ cations didn't situate at the interstitial sites.

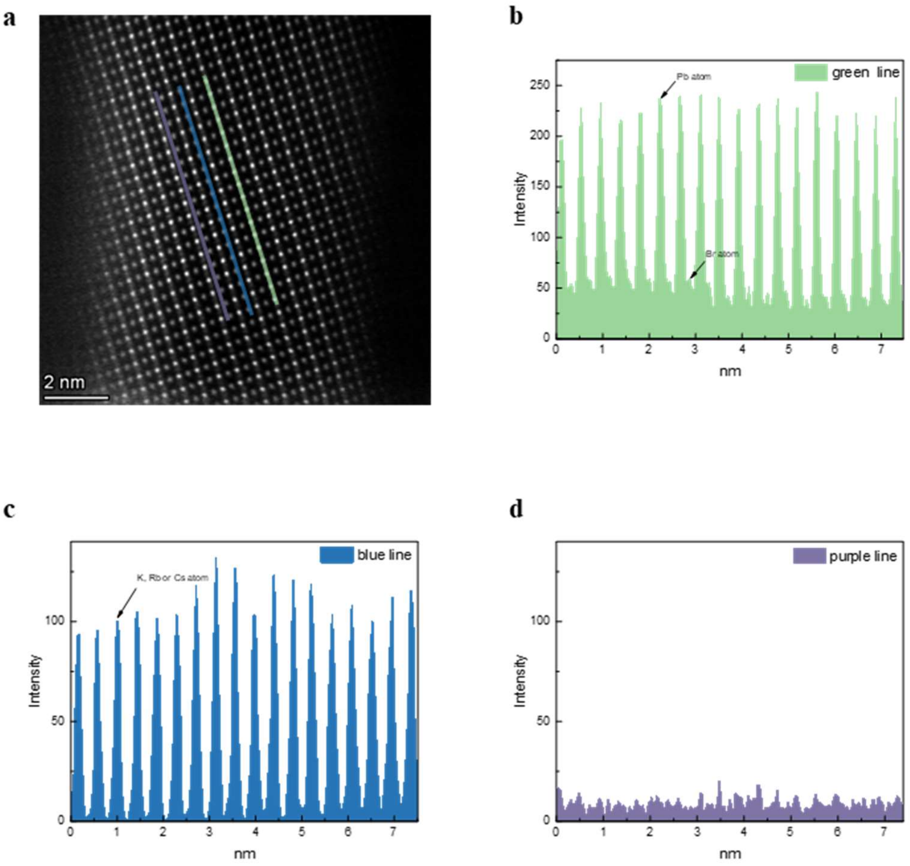

Supplementary Figure 4. Atomically resolved low-dose HRTEM image of alkali metals doped PeQWs and profile of signal intensity at different position.

12. There is a typo in the caption of Figure S2. It should be Cs4PbBr6 not Cs2PbBr6.

Response: In light of the reviewer's comment, we have revised the caption of Supplementary Figure S2.

Revision: Supplementary Figure 2 in the revised supplementary information.

Reviewer #2 (Remarks to the Author):

The manuscript describes the results of alkali metal doping studies of metal halide perovskites towards ultrapure and efficient light emitting diodes. The main founding presented in the manuscript is demonstration of the highly pure and efficient electroluminescence.

The results are very well presented. The conclusions are very much supported by the data. I recommend the manuscript for publication after minor revision.

Response: We sincerely thank the reviewer for the positive recognition.

(1) Beside the emission bandwidth, the emission peak wavelengths are also important to wide-color-gamut display applications, maybe 478nm, 532nm, 638nm for the blue, green and red ones.

Response: We thank the reviewer for the valuable advice. The wavelengths of the Rec. 2020 primary colors are 467 nm (blue), 532 nm (green) and 630 nm (red). In our previous report (Nature Communications 14, 4611 (2023)), we have demonstrated red/green/blue all-inorganic PeQWs-based LEDs. However, the emission wavelengths are 478 nm, 512 nm and 630 nm. In order to satisfy the Rec. 2020 requirements, we will further increase the chloride concentration for $CsPb(Br_{1-x}Cl_x)_3$ QWs to realize 467 nm emission, use $FAPbBr_3$ QWs to get 532 nm emission and $CsPb(Br_{1-x}I_x)_3$ QWs for 630 nm emission.

(2) The operating half lifetimes are relatively low, especially for the blue ones. Could authors comment on this point.

Response: Thank you very much for raising this important question. After Rb^+ and K^+ doping, the operating half lifetimes were improved compared with our previous report (Nature Communications 14, 4611 (2023)). Previously, the operating half lifetime was only a few minutes under 100 nits. In this study, we conducted an operating half-lifetime test at 1000 nits,

indicating that the lifespan could be much longer when operating at 100 nits. We believe that the short operating half lifetime is caused by the high operating voltages because the turn-on voltage is relatively high. We attribute the relatively high turn-on voltages to the energy level mismatch between PeQWs and Al electrode as well as the thin barrier layer of Al_2O_3 (~5 nm). To further enhance long-term stability and decrease the working voltage, it is imperative to eliminate the thin barrier layer of Al_2O_3 situated between PeQWs and Al electrode as well as inserting a proper electron transporting layer. One potential remedy involves the implementation of the device structure detailed in a study (Sci. Adv. 2017;3: e1700738). In this scenario, the proposed device structure would consist of a Si wafer/Al/compact TiO_2/perovskite QWs/TPAC/HAT-CN/ITO configuration.

(3) Why choose HAT TAPC as hole transporting layers instead of PEDOT: PSS?

Response: We thank the reviewer for the question. The purpose of using HAT-CN and TAPC as hole injection layer and hole transporting layer has been illustrated in our previous report (Nature Communications 14, 4611 (2023)). We deposited 1,3,5-tris(bromomethyl)-2,4,6-triethylbenzene (TBTB) doped TAPC through a chlorobenzene solution and deposited HAT-CN through thermal evaporation. Both TBTB and TAPC can passivate the top surface of PeQWs. The 20 nm HAT-CN was used to improve hole injection and reduce the sputtering damage to the PeQWs. We didn’t use PEDOT: PSS because depositing PEDOT: PSS involves a water-based solution, which is incompatible with our fabrication process.

(4) The open voltages of these LEDs are relatively high. Could authors comment on this point.

Response: We appreciate the reviewer for raising this important question. As we mentioned in the response to Q2 above, we attribute the relatively high turn-on voltages to the energy level mismatch between PeQWs and Al electrode as well as the thin barrier layer of Al_2O_3 (~5 nm). To further enhance long-term stability and decrease the working voltage, it is imperative to eliminate the thin barrier layer of Al_2O_3 situated between PeQWs and Al electrode as well as

inserting a proper electron transporting layer.

Reviewer #3 (Remarks to the Author):

In this article, Fan and co-workers report efficient sky blue (476 nm, 485 nm, and 490 nm) and green perovskite LEDs (512 nm) using nanopatterned alumina templates. Their green devices showed a remarkable EQE of ~30%. Using DFT, the authors demonstrate that the Al-Br and Pb-O bonds at the interface are responsible for reduced defects and enhanced PLQYs. At the same time, the authors employ Rb+, and K+ dopant ions to fabricate inorganic perovskite layers in patterned templates. Overall, this work is interesting, and the authors performed numerous studies to analyze the perovskite layers and consequently constructed efficient devices. However, I have several concerns that are listed below, which need to be addressed before publication in Nature Communications.

Response: We are grateful to the reviewer for the supportive comments.

1) First, it is unclear where the enhancement in EQE is coming from. Is it due to the templated structure leading to an improved optical coupling or due to the doping? I am curious to see how undoped devices behave in the templated structure. The authors provide no convincing evidence that doping benefits the device performance.

Response: Thank you very much for the beneficial comments. In this work, the EQE enhancement mainly originated from the improved quality of PeQWs, rather than the improved outcoupling efficiency. We have already demonstrated the advantages of using NAT template and studied the outcoupling efficiency in our previous report (Nature Communications 14, 4611 (2023)). The recipe we used in our previously report was RbBr: CsBr: $PbBr_2$: $PbCl_2$=0.1: 1.4: x: y and the one we used in this paper is KBr: RbBr:CsBr: $PbBr_2$: $PbCl_2$=0.2: 0.3: 1: x: y. According to the FDTD simulation, the outcoupling efficiency in the previous report was approximately 80% while the value has been slightly improved to around 85% in this paper. For green emitting devices, the maximum EQE was improved from 26% to 30%.

Based on your suggestion, we fabricated devices without doping. The recipe we used is CsBr:

$PbBr_2$=1:1 in DMSO. Compared with doped devices, undoped devices show inferior performance. Below please find a representative data of an undoped device with the maximum EQE of only 18.8%. And its maximum luminance is around 4000 nits.

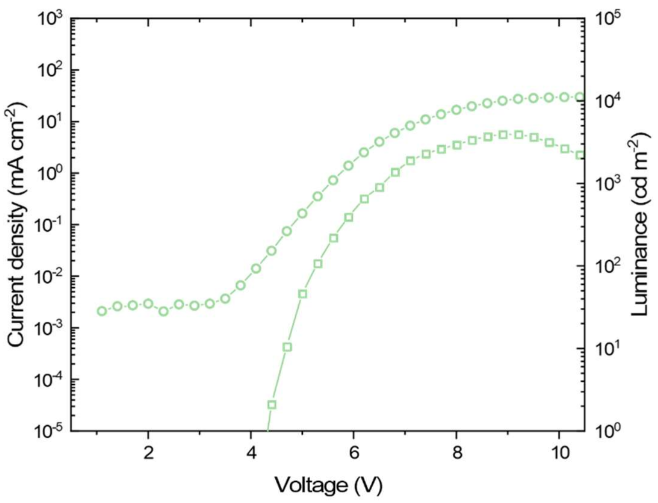

Figure R8. Current density (*J*)-voltage *(V)*-luminance *(L)* curves for undoped LEDs.

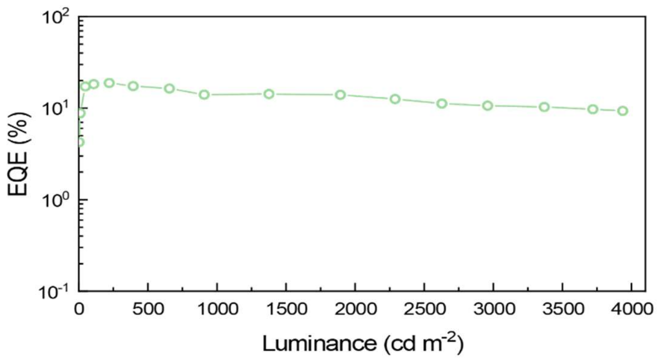

Figure R9. External quantum efficiency *(EQE)*-luminance *(L)* curves for undoped LEDs.

2) Also, the authors used XRD peak shift to support the substitutional doping of Rb+ and K+, like many other reports, but they claim that this is unambiguous evidence of substitutional doping. This evidence is not enough to support their bold claims. Also, why do you see a peak shift in those planes, where the reflections are from octahedra and A-site has no major role? Moreover, Rb and Cs have similar ionic radii. The shift in the peaks, sometimes, is also

attributed to the "height effect," where it is possible that the resultant templated structure may have different sample heights.

Response: We appreciate the reviewer for suggesting this important point. The samples we used for XRD measurement were fabricated in the same batch and the measuring condition was the same. Moreover, the NAT template was fabricated under identical conditions, with the same solution temperature, growth voltage, and duration. Thus, we believe that NAT templates have almost the same thickness and morphology and the "height effect" can be avoided.

The change in A-site cation will definitely alter the lattice constant of perovskite, causing a peak shift in the XRD pattern. Since the radius of Rb^+ and K^+ cation is smaller than Cs^+ cation, the lattice constat will reduce when Cs^+ cation is replaced by Rb^+ or K^+ cation. That's why we can observe a peak shift in the XRD pattern. Similarly, in the in-plane intensity profiles extracted from the GIWAXS, we also notice a peak shift, meaning that the doping of Rb^+ or K^+ reduces the lattice constant of perovskite. Moreover, the measured average interplane spacing of perovskite lattice in the atomically resolved low-dose HRTEM images reduces from 6.06 Å to 5.97 Å after doping. With the XRD, GIWAXS, atomically resolved low-dose HRTEM, EDS mapping, UPS results and DFT simulation, we are confident to conclude that Rb^+ and K^+ can substitute Cs^+ at A-site.

3) The primary blue 476 nm LEDs show a remarkable max EQE of ~17%, whereas their PLQY is only 66%. In addition, there is an insulating Al_2O_3 in the template. Despite these, the performance is very high. To me, this indicates that there was nearly no loss in the free carriers, and the majority of the injected carriers led to the emission. Can you explain what is responsible for this? Are these PLQYs measured in templated structures? Generally, mixed halides tend to suffer more from defects, which are detrimental in device EQE, but authors seem to have no such issues.

Response: Thank you very much for providing detailed comments. The EQE is defined by the equation below:

$$EQE = PLQY \times \text{outcoupling efficiency} \times \text{charge balance factor} \times \text{singlet-triplet ratio}$$

Assuming singlet-triplet ratio as 1, the EQE depends on PLQY, outcoupling efficiency and charge balance factor. Due to the energy level mismatch between PeQWs and hole transporting layer, PeQWs and Al electrode as well as the thin barrier layer of Al_2O_3 (~5 nm), the charge balance factor is below 1, indicating that lots of injected carriers are lost before they are injected into PeQWs. Assuming the perfect charge balance and singlet-triplet ratio as 1, the EQE depends on PLQY and outcoupling efficiency. According to our FDTD simulation, the outcoupling efficiency of our PeQWs is around 85 %. Therefore, the EQE can be 56.1 % thus 43.9 % of injected carriers don't contribute to light emission which is still a significant loss.

Supplementary Table 5. Summary of simulated out-coupling efficiency for alkali metals doped PeQWs-based LEDs.

Perovskite QWs	Dipole polarization	Out-coupling efficiency (%)	Average out-coupling efficiency (%)
Rb, K: $CsPb(Br_xCl_{1-x})_3$ QWs (476 nm)	HD	86.98	85.01
	VD	81.06	
Rb, K: $CsPb(Br_xCl_{1-x})_3$ QWs (483 nm)	HD	87.11	85.32
	VD	81.73	
Rb, K: $CsPb(Br_xCl_{1-x})_3$ QWs (490 nm)	HD	87.12	85.35
	VD	81.81	
Rb, K: $CsPbBr_3$ QWs (512 nm)	HD	86.68	85.25
	VD	82.39	

The PLQYs were measured in the templated structure. Without the templated, the PLQY of perovskite was very low in our previous report (Nature Communications 14, 4611 (2023)).

Actually, our mixed halide PeQWs are also affected by defects caused by chloride doping. In our previous report (Nature Communications 14, 4611 (2023)), the PLQYs for blue emitting PeQWs are 24% (478 nm) and 73% (491 nm). After Rb^+ and K^+ doping, the PLQYs were improved to 66.2% (476 nm), 71.5% (483 nm) and 81.6% (490 nm). We attribute the improved PLQYs to the increased exciton binding energy and the reduced defect density.

4) Do you see a shift the EL peak with time when mixed halides are used in a device?

Response: Thank you for the valuable comments. As can be seen from the Supplementary Figure 30, we didn't observe obvious EL peak shift and emission linewidth broadening during the operating lifetime test for mixed-halide PeQWs-based LEDs.

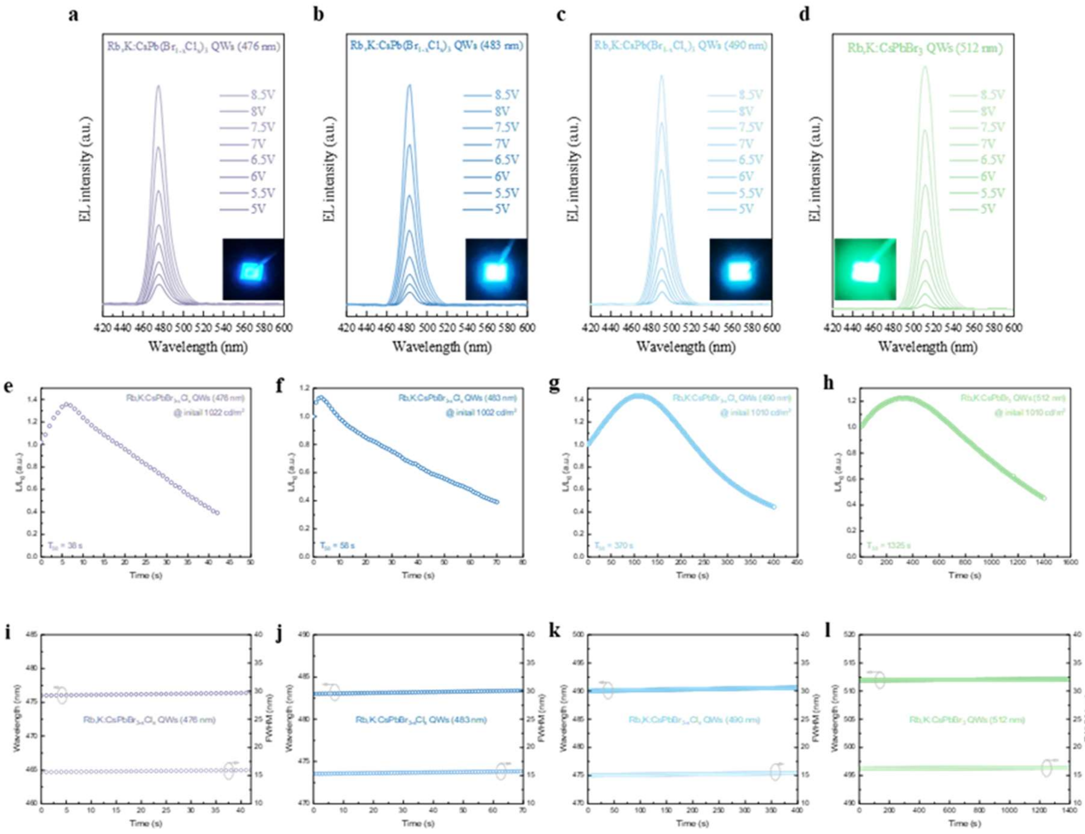

Supplementary Figure 30. a-d, EL spectra of alkali metals doped PeQWs-based LEDs operating at different voltages. Inset, electroluminescence images of for alkali metals doped PeQWs-based LEDs. e-h, T_{50} lifetime measurements for alkali metals doped PeQWs-based LEDs. i-l,

EL peak position FWHM evolution as a function of time.

Other minor points

1) The PLQYs and EQEs are reported as 4 sig figs, which is not appropriate unless the authors perform a statistical analysis to prove that all the numbers are significant. So, I encourage the authors to round it to 2 sig figs.

Response: We appreciate the reviewer for the valuable suggestion to improve the presentation of our experimental results. After reading several published literatures, we found that most literature presented the efficiency values in 3 significant figures. As the EQE has been progressively improved in recent literature, the difference in EQE among reports is getting smaller. In order to fairly and accurately reflect our progress, we decided to round the PLQYs and EQEs to 3 significant figures.

Revision: Page 2 line 42, page 4 line 86, page 12 line 263-264 in the revised manuscript.

2) The figure legends are hard to read due to the font size and the color in Figures 3 and 4

Response: We appreciate the valuable comment. We made the front bold and enlarged the font size so that the figure legends can be seen clearly.

Revision: Figure 3 and 4 in the revised manuscript.

Response to the Reviewers' Comments

[Nature Communications NCOMMS-24-55954-A]: Ultrapure and efficient electroluminescence in alkali metal doped inorganic perovskite quantum wires arrays

We would like to thank the reviewers and the editors for their valuable comments on our work. Such productive feedback from the experts in this field helped us to improve our manuscript. Here we provide a point-by-point response below to the reviewers' questions. We also revised our manuscript to comply with the reviewers' comments and have highlighted the revised parts in red.

Reviewer #1 (Remarks to the Author):

I appreciate the efforts made by the authors, but unfortunately, I am not convinced.

I agree that utilizing identical techniques to those employed by others does not inherently indicate a lack of novelty. However, my primary concern is that the paper's conclusion—that Rb and K can replace Cs—is not novel. Furthermore, the authors assert that 20% and 13.3% of Rb and K have been incorporated into the crystal lattice, yet the photoluminescence (PL) and electroluminescence (EL) exhibit minimal changes (e.g., perovskite emission remains at 512 nm). This is certainly unreasonable. As a large number of doping results in lattice construction and significantly enlarged bandgap. Here is more literature for the references (e.g. Nanoscale, 2018,10, 3429-3437; Nature Communications 2019, 10, 1868), where 20% Rb incorporation results in a big shift in the bandgap.

I would expect to observe a continuously enlarged bandgap of perovskites with varying Rb/K concentrations without the aid of Cl ions. It is possible that only a small fraction of Rb or K ions are successfully integrated into the lattice at high doping levels, while the majority form aggregates. The XRD results are not particularly informative in this scenario, as aggregates may be amorphous and thus not easily detectable. Additionally, HRTEM is limited in its ability

to provide comprehensive insights, as it examines only very small regions of the material. In my view, synchrotron-based nano X-ray fluorescence microscopy and solid-state NMR would be very helpful to detect whether Rb/K cations are uniformly distributed into perovskites or being aggregates, upon different Rb/K content. Besides, XPS may also help if the Rb/K doping ratio is indeed as high as what the authors claim. It is unclear why the authors did not include Pb, Rb, and K core-level spectra in Figure S9, as this data would be critical for supporting their claims, especially for those with high Rb/K contents.

In addition, the three papers I mentioned aimed to show that for different cases, including perovskite stoichiometry and K/Rb doping level, the role of these alkalic ions can be different.

Response: We appreciate the reviewer bringing up these important points. We have to rephrase that concept of Rb^+ and K^+ substituting Cs^+ at A-site is not new in the research filed. As we discussed in our previous response letter, even though some reports suggest that Rb^+ and K^+ can replace Cs^+ at A-site with limited evidences, the prevailing consensus leans towards the contrary viewpoint. Similar to the work cited in the previous response letter (Joule, Volume 7, Issue 12, 2905 - 2922), the key point lies in providing unambiguous and sufficient evidences supporting that Rb^+ and K^+ can substitute Cs^+ at A-site in our PeQWs, which no one has ever done before. Besides, this is only one of the highlights of our paper. The others are: 1) We fabricate highly efficient PeLEDs based on alkali metals doped PeQWs with top-emission device configuration. The EQE values set a record among top-emitting PeLEDs which are exceptionally well-suited for wide-color-gamut display applications. 2) The PeLEDs has a narrow full-width of half maximum (FWHM) of ≤ 16 nm, demonstrating high color purity.

In our previous response letter, we indicated that the doping concentrations of Rb^+ and K^+ were 20% and 13.3%, respectively. However, it's important to clarify that these percentages refer to the doping concentration in the precursor solution and it does not mean that 20% and 13.3% of Rb^+ and K^+ were incorporated into the crystal lattice. We conducted the elements analysis on the selected area from the EDS mapping (Figure R1). The actual doping concentrations of Rb^+ and K^+ are approximately 7.0% and 2.4%, respectively. We believe that a fraction of Rb^+ and

K^+ was casted away along with the precursor solution during the spin-coating process. In this case, it's completely reasonable that the PL and EL of Rb, K: $CsPb(Br_{1-x}Cl_x)_3$ QWs exhibit minimal changes.

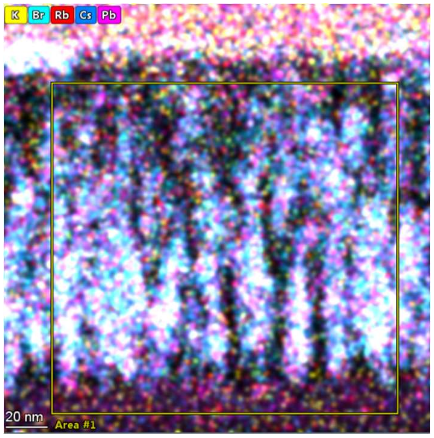

Figure R1. EDS mapping of Rb, K: $CsPb(Br_{1-x}Cl_x)_3$ QWs.

As suggested by the reviewer, we fabricated bulk $CsPbBr_3$ thin film and $CsPbBr_3$ QWs with varying concentrations of alkali metal doping (Figure R2). For bulk $CsPbBr_3$ thin films, the PL of bulk $CsPbBr_3$ doped with 20% K^+ blue-shifted because of the formation of non-emissive potassium lead bromide impurity phase in the presence of excess amount of K^+, whereas the PL remained almost unchanged as the doping concentration increased (Adv. Optical Mater. 2020, 8, 2000742). In accordance with existing literatures (Nanoscale, 2018,10 and 3429-3437; Nature Communications 2019, 10, 1868), the PL of Rb^+ doped thin films exhibited a continuous blue-shifting as the doping concentration increased. In stark contrast, we observed a continuous shift in the bandgap towards higher energy level as the concentration of Rb^+ or K^+ increased. Such a shift is facilitated by the AAO template which aligns well with our theory proposed in the manuscript. We believer that the K^+ doped PeQWs exhibit a minor PL shift because of the large difference in the doping concentration of the precursor solution and the fabricated PeQWs.

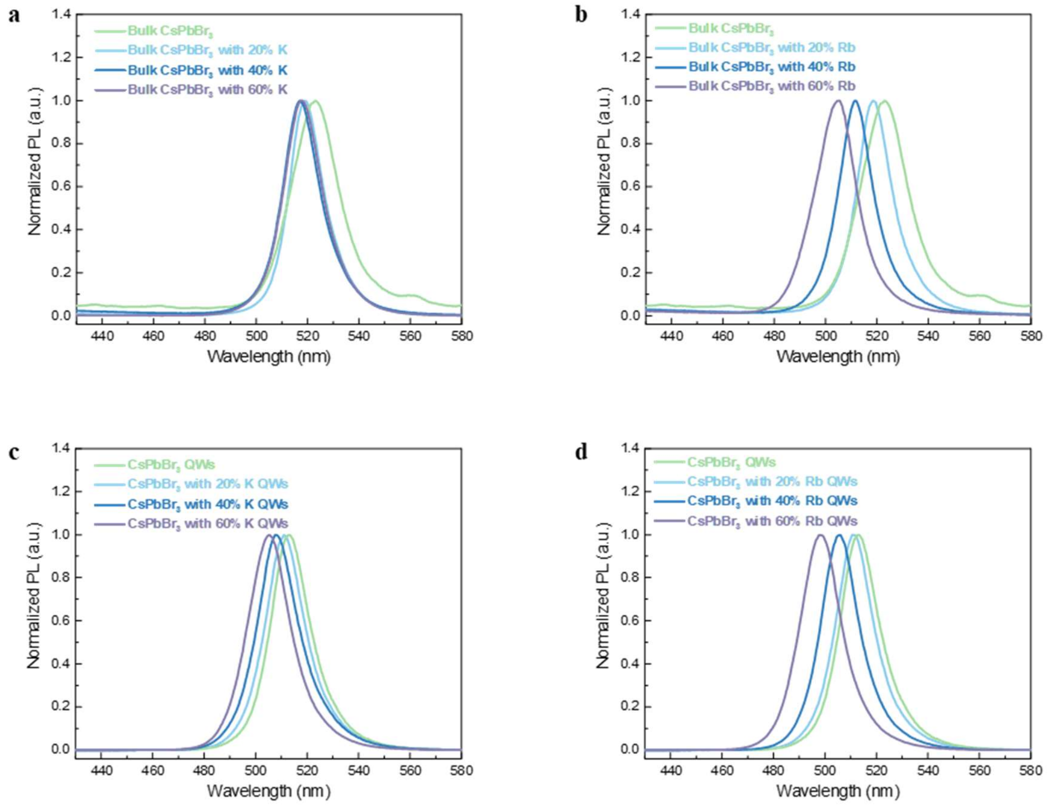

Figure R2. Photoluminescence spectra of bulk $CsPbBr_3$ and $CsPbBr_3$ QWs with different concentrations of alkali metal doping.

We agree that XRD results along cannot be considered as compelling evidence. Therefore, we have supplemented our findings with additional tests, such as GIWAXS, HRTEM, DFT simulation and XPS, to provide corroborative evidence. Through undoped PeQWs HRTEM images, we did find 0-dimension Cs_4PbBr_6 in the AAO channel which was not detected in the XRD pattern (Figure R3). We checked several positions and found more than two Cs_4PbBr_6 QWs. Given the possibility of finding 0-dimension Cs_4PbBr_6 in undoped PeQWs, we expect to find Rb-rich and K-rich compounds, whether they exist in a crystalline or amorphous state, if they aggregate within the AAO channel. Nevertheless, none of these were detected in alkali metal-doped PeQWs.

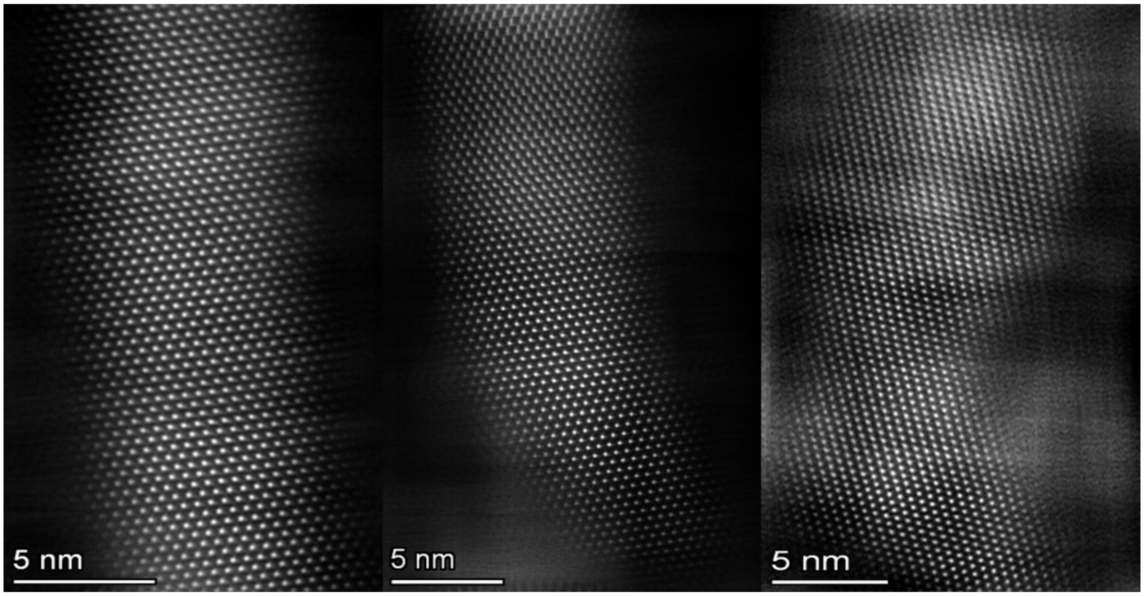

Figure R3. HRTEM images of Cs_4PbBr_6 QWs.

We are grateful to the reviewer for recommending synchrotron-based nano X-ray fluorescence microscopy and solid-state NMR as additional tests. However, accessing these tests can be quite hard for us, particularly in the case of synchrotron-based nano X-ray fluorescence microscopy. With all due respect, we don't think synchrotron-based nano X-ray fluorescence microscopy is helpful because the resolution can only reach 10-30 nm at state-of-the-art facilities (Adv. Energy Mater. 2020, 10, 1903170). As can be seen from Figure R4, the pore size of the AAO is only ~6.4 nm. Therefore, it's impossible to detect whether Rb and K cations are aggregates within the AAO channel. Furthermore, we do not conclude that the Rb and K cations are uniformly distributed in the PeQWs.

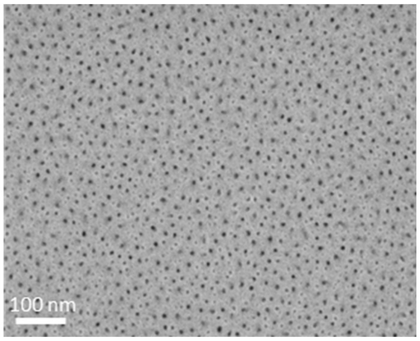

Figure R4. Top-view SEM image of NAT template.

For XPS measurements, we did include Pb core-level spectra in the Supplementary Figure 9 and Rb/K core-level spectra in the main Figure 1. Please check again.

For more responses:

1. The authors claimed that their perovskites are quantum wires, despite the emission of $CsPbBr_3$ being in the green spectrum. They attribute this to the confinement dimensionality being lower than that of quantum dots (QDs). While this rationale is correct, it raises a critical question: if the confinement is not sufficiently strong, is it appropriate to classify these materials as quantum wires? This issue is analogous to the distinction between nanocrystals and quantum dots. Additionally, the authors reference a Nature publication concerning the in-situ formation of perovskite QDs. In that study, the authors successfully fabricated quantum-confined $CsPbBr_3$ QDs exhibiting deep blue emission, which starkly contrasts with the current findings. The authors also cite their previous work; however, it does not make their argument to be correct. Regarding questions Q1 and Q2, I recommend that the authors consult the study by Annamaria's group (J. Am. Chem. Soc. 2014, 136 (51), pp. 17730–17733) for further insights and clarification.

Response: We appreciate the reviewer bringing up this important point. The reviewer provided a study done by Annamaria's group (J. Am. Chem. Soc. 2014, 136 (51), pp. 17730–17733) for us to gain more insights into the quantum confined perovskite. However, this paper mainly studied the influence of morphology of $MAPbI_3$ thin film, from a molecular to mesoscopic level, on the luminescence properties. The representative samples investigated in this paper have an observed average size of crystallite dimension ranging from ~200 nm to >2 μm. Furthermore, the obtained PL lifetime was reduced from >100 ns to 2 ns as the crystallite size reduced to ~200 nm. In this case, this paper is unrelated to quantum confined perovskite and certainly it cannot offer any insights or clarifications to the distinction between nanocrystals and quantum dots.

For the Nature paper we cited in the previous response letter (Nature 612, 679–684 (2022)),

the key innovation that sets this paper apart from existing research is that the authors demonstrated the direct synthesis-on-substrate of suitably coupled, monodispersed and ultrasmall perovskite QDs. The tunability of the band gap in their perovskite QDs continues to align with the theoretical and experimental models outlined in the literature (Nature Mater 2, 517–520 (2003)). It is evident that the confined perovskite QDs, which showcase deep blue emission, are in line with the latest research discoveries. Please check the figure presented in the Nature paper (Figure R5). As stated in our previous response letter, perovskite QDs with 6.4 nm diameter emit green light peaking at ~515 nm. While these perovskite QDs fall in the category of QDs, they reside within the intermediate quantum confinement regime due to their diameter being close to the exciton Bohr diameter of $CsPbBr_3$ (7 nm).

[FIGURE REDACTED]

Figure R5. Relationship among ligand concentration, the peak wavelength of QD films and the size of the QDs. (Nature 612, 679–684 (2022))

Experimental versus theoretical size dependence of the bandgap energy with quantum confinement regimes noted in relation to the Bohr diameter is illustrated in a paper (Chem. Mater. 2017, 29, 8, 3644–3652). When the perovskite QDs' diameter is 7.3 nm, the ratio to exciton Bohr diameter of $CsPbBr_3$ is approximately 1, making them fall under the intermediate quantum confinement regime (Figure S6). Similarly, our PeQWs have a diameter of around 6.4 nm, with a ratio to the exciton Bohr diameter of $CsPbBr_3$ at 0.91, indicating that our PeQWs exhibit intermediate quantum confinement. As depicted in Figure R7, there is an obvious PL peak shifting from 523 nm to 512 nm due to the intermediate quantum confinement effect. Besides, Professor Feng GAO, a leading expert in organic and perovskite semiconductors field,

previewed our previous Nature Photonics paper titled “Large-scale perovskite light-emitting diodes enabled by quantum-wire arrays: One step closer to commercialization” (Matter, Volume 5, Issue 8, 2428 - 2430), considering our material as quantum wires. Therefore, we are confident in classifying our perovskite as perovskite quantum wires.

[FIGURE REDACTED]

Figure R6. Experimental versus theoretical size dependence of the bandgap energy with quantum confinement regimes noted in relation to the Bohr diameter. (Chem. Mater. 2017, 29, 8, 3644–3652)

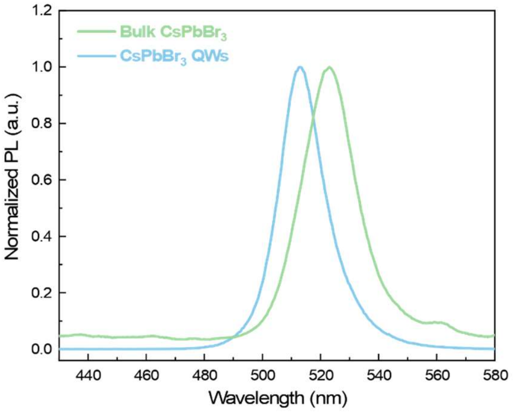

Figure R7. Photoluminescence spectra of bulk $CsPbBr_3$ and $CsPbBr_3$ QWs.

2. As I said, if the perovskite samples in this paper are QWs and the binding energy is high, we should see a big difference compared to bulk in recombination dynamics. Fluence dependent

PLQY or fluence dependent decay are necessary to extract the recombination constants.

Response: We appreciate the reviewer for providing valuable feedback. We will address this together with comment 6.

3. For Q4, While the authors provide extensive discussion on Q4, they fail to address my concern. I would like to see a continuously shifted bandgap of AAO-$CsPbBr_3$ with increasing the doping ratio of Rb or K. Demonstrating such a shift would serve as compelling evidence that the incorporation of Rb and K is facilitated by AAO. Additionally, including a comparison with an AAO-free sample would enhance the validity of this claim by clearly illustrating the role of AAO in the doping process.

Response: We are grateful to the reviewer for the helpful advice. We measured the PL spectra of newly fabricated PeQWs and bulk perovskite thin films. The results have been presented and discussed above.

4. For Q5, the resolution of EDS and its ability to distinguish different metallic element is not good enough. For instance, from the mapping of Rb, it seems that it is concentrated in top contact, but this is not true. Combining XRD and GIWAXS, I agree that K and Rb cations can enter the lattice. However, it does not imply that all the K and Rb ions can do that. The authors should give more evidence to demonstrate that the Rb and K are not concentrated somewhere, especially for high doping levels such as 20%.

Response: We thank the reviewer for pointing out this issue. We have to emphasize that FIB (focus ion beam) cutting is needed before the capturing the cross-sectional TEM images. To minimize the damage caused by FIB cutting to the sample, we need to deposit 300 nm Cu as protection layer. During the FIB cutting, it's possible that the elements from PeQWs migrate to upper layer. Besides, there might be an elemental signal overlap between protection layer and Rb/K. If you check the EDS mapping on these two papers (Nature 555, 497–501 (2018)

and ACS Energy Lett. 2018, 3, 11, 2671–2678), evident signals of K or Rb can be observed in the protection layer. Therefore, it's normal that elements from PeQWs were observed in the top contact.

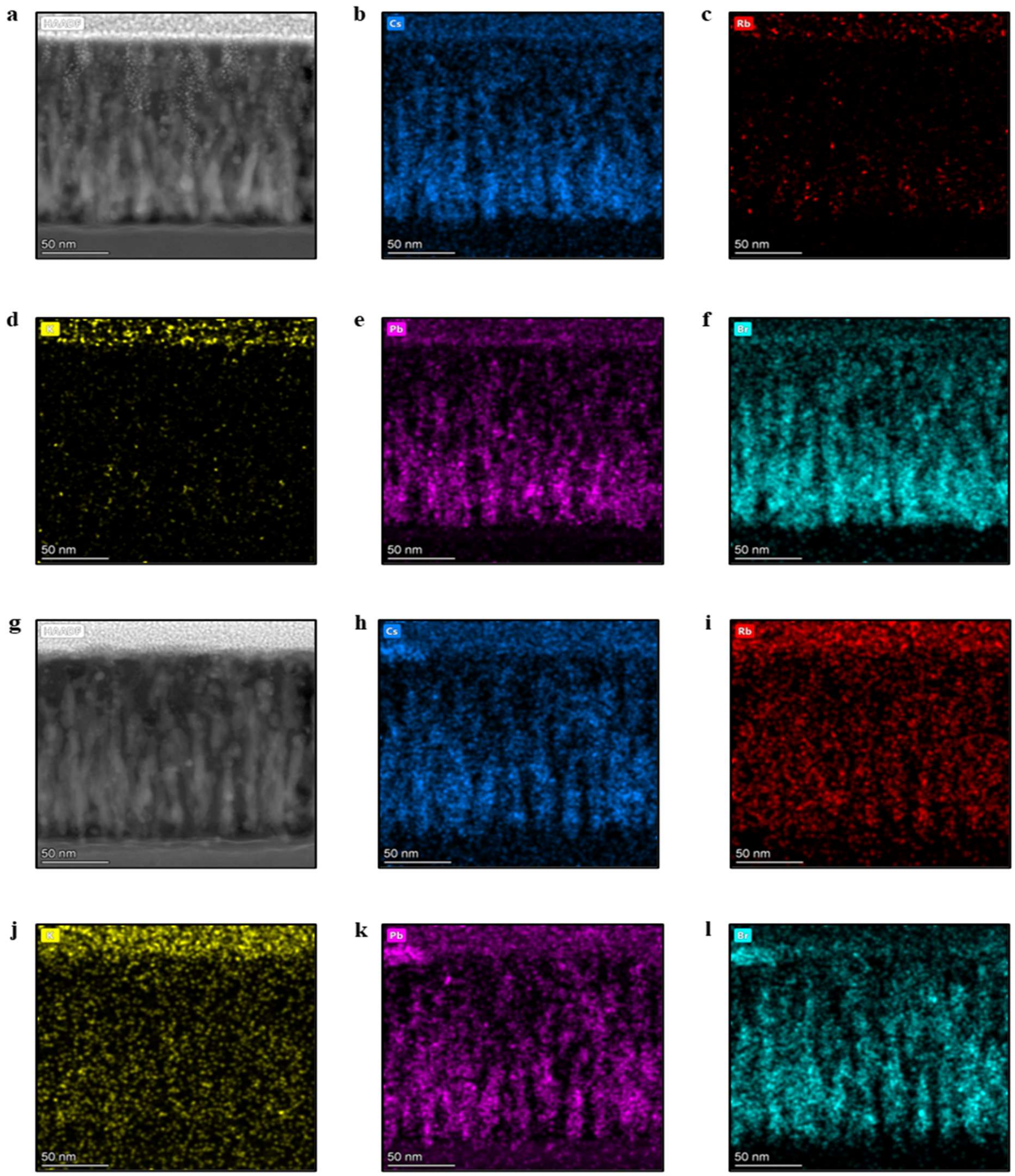

Supplementary Figure 3. TEM and EDS mapping of pristine $CsPbBr_3$ QWs and Rb^+, K^+: $CsPbBr_3$ QWs. a, g, Cross-sectional TEM images of pristine $CsPbBr_3$ QWs and Rb^+, K^+: $CsPbBr_3$ QWs. The corresponding EDS mapping images of b, h, Cs, c, i, Rb, d, j, K, e, k, Pb

and f, l, Br elements.

In order to further demonstrate that Rb^+ and K^+ cations are not concentrated somewhere in the PeQWs, we conducted time-of-flight secondary-ion mass spectroscopy (ToF-SIMS) to investigate the cation distribution. As can be seen from Figure R8 (a), only very week Rb^+ and K^+ signals were detected for undoped PeQWs. In stark contrast, the signals of Rb^+ and K^+ cations exhibit almost the same tendency with that of Cs^+, indicating that Rb^+ and K^+ cations are not localized in any specific region within the alkali metal doped PeQWs (Figure 8 (b) and (c)). Otherwise, we would expect to observe an abrupt change in the intensity of Rb^+ and K^+ compared with that of Cs^+ in the ToF-SIMS profiles. Amazingly, Cs^+, Rb^+ and K^+ cations show a uniform distribution vertically within PeQWs. Additionally, ToF-SIMS 3D tomography is depicted in Figure R9. We didn't observe any concentrated signals of Rb^+ and K^+ in the area of 50μm*50μm. Thus, we are confident to conclude that Rb^+ and K^+ cations are not aggregated in any specific region within the alkali metal doped PeQWs.

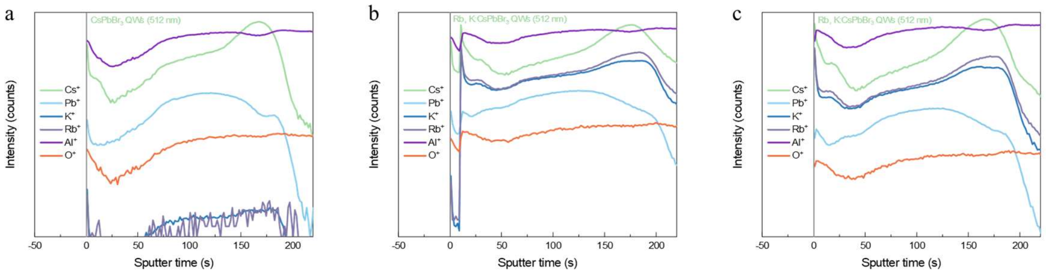

Figure R8. ToF-SIMS profiles of PeQWs with or without alkali metals doping.

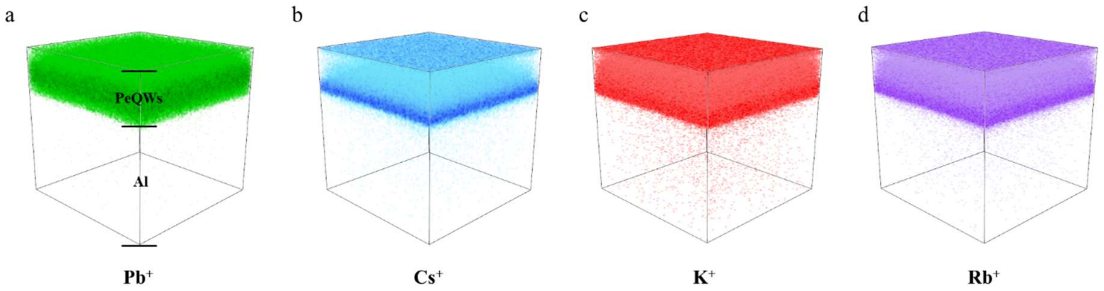

Figure R9. ToF-SIMS 3D tomography of alkali metals doped PeQWs

5. For Figure S14, please unify the photon energy range on the horizontal axis.

Response: We appreciate the reviewer for the valuable suggestion to improve the presentation of our experimental results. We have unified the photon energy range on the horizontal axis.

Revision: Supplementary Figure 14 in the revised supplementary information.

6. For the PL lifetime, I still believe it is over-interpreted. There is no difference between 7 and 9 ns. Furthermore, considering that an increase in Rb/K or Cl doping typically leads to an enlarged exciton binding energy, one would expect a corresponding acceleration in recombination rates. This anticipated faster recombination contradicts the observed PL decay behavior. Therefore, the PL decay alone cannot be used for understanding the defect physics and recombination dynamics. Other tests including fluence-dependent PLQY/decay or TA to extract the dynamics are important.

Response: The reviewer's concern is acknowledged. Given the similarity between comment 2 and 6, we decided to address them together. As suggested by the reviewer, we performed fluence-dependent decay to analyze the charge-carrier dynamics. The recombination dynamics of the charge-carrier density $N(t)$ can be described in the form of:

$$\frac{dN(t)}{dt} = -k_1 N - k_2 N^2 - k_3 N^3$$

where k_1 is the monomolecular recombination decay constant (which is relevant to both trap-assisted recombination and geminate recombination of exciton); k_2 is the bimolecular recombination decay constant (which is related to radiative recombination); and k_3 is the Auger recombination decay constant. According to the well-established method (Nat Commun 12, 336 (2021)), the second order (k_2) and third order (k_3) can be fitted through global fitting, and the results have been displayed in Table R1. As shown in Table R1, first-order recombination constant, k_1, reduces for alkali metal doped perovskite and PeQWs, which is well accordance

with our SCLC measurements. It is worth mentioning that the bimolecular recombination decay constant is one-order-of magnitude higher for PeQWs when compared to the bulk perovskite thin film, reconfirming that the recombination rate has been dramatically enhanced, and in good agreement with the temperature dependent PL measurement. Furthermore, the Auger recombination is also notably suppressed.

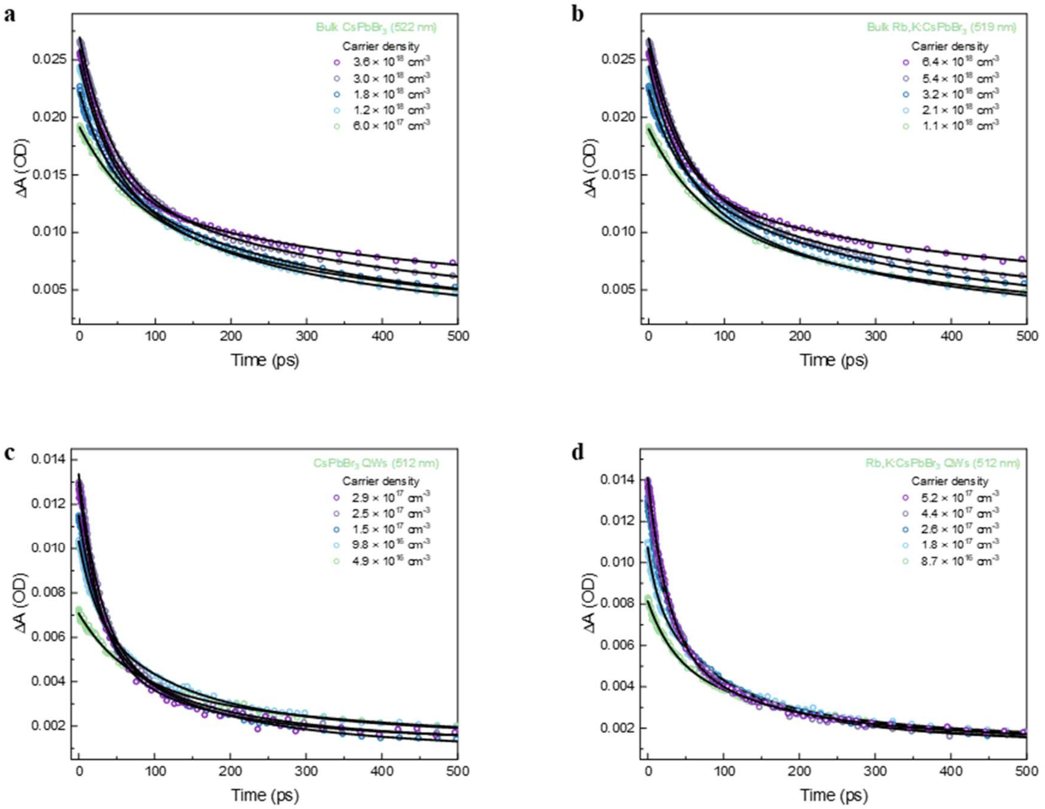

Figure R10. The TA bleaching kinetics at emission wavelength measured under various pump fluences for bulk $CsPbBr_3$ and $CsPbBr_3$ QWs with or without alkali metal doping.

Table R1. Monomolecular (k_1), bimolecular (k_2) and trimolecular (k_3) recombination constant of bulk $CsPbBr_3$ and $CsPbBr_3$ QWs with or without alkali metal doping.

	**k_1 (s^{-1})**	**k_2 (cm^{-3} s^{-1})**	**k_3 (cm^{-6} s^{-1})**

Bulk $CsPbBr_3$ (522 nm)	2.1×10^{7}	3.0×10^{-11}	1.4×10^{-29}
Bulk Rb, K:$CsPbBr_3$ (519 nm)	1.1×10^{7}	8.9×10^{-12}	2.3×10^{-30}
$CsPbBr_3$ QWs (512 nm)	6.1×10^{6}	9.5×10^{-11}	4.8×10^{-29}
Rb, K:$CsPbBr_3$ QWs (512 nm)	4.1×10^{6}	1.4×10^{-10}	1.1×10^{-29}

Revision: Page 10, line 215-219 in the revise manuscript. Supplementary Figure 20 and Supplementary Table 1 in the revised supplementary information.

7. For Q9, I do not believe a test for shelf stability in dry air (10-20% humidity) is meaningful. There are several reasons:

i) Although OLED emitter films exhibit instability in ambient air, this does not present a significant barrier to their commercialization. In practical applications, protective encapsulation effectively mitigates these stability issues.

ii) State-of-the-art perovskite solar cells, lots of which are also mixed halide perovskites, can be stable even in water. Basically, a test close to 85°C/85% relative humidity test for thousands of hours is required.

Besides, in our lab, the mixed halide perovskite emitters can be stored in dry air for several months without any negative effect on their optical properties. With all respect, I do not think anyone cares about that.

Response: We thank the reviewer for this critical comment. We have no intention to compare the stability of our PeQWs with OLED emitter and perovskite solar cells absorber under ambient condition because it's unreasonable to evaluate them within the same category. The reason why we conducted the shelf stability test under dry air is to demonstrate that our PeQWs can endure ambient condition for more than 1000 hours without encapsulation. Besides, the alkali metal doped PeQWs exhibit higher stability compared with our pervious report (Nat.

Photon. 16, 284–290 (2022)). Later, we also performed the shelf stability test in humid air (Figure R11). The PL intensity of our PeQWs reduced slightly after one month.

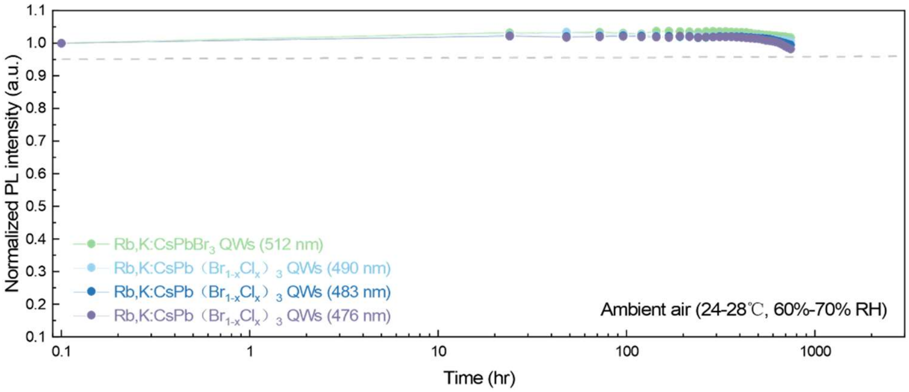

Figure R11. Environmental photoluminescence stability under ambient condition for alkali metal doped PeQWs.

Currently, there is no standardized environmental stability test for perovskite emitter. Researchers evaluate the environmental stability under different conditions depending on different scenarios. Many researchers conducted environmental stability test for perovskite emitter in dry air, observing a significant decrease in PL intensity after several hours (Nature 631, 73–79 (2024), Nature **622**, 493–498 (2023), Sci. Adv.9, eadh2140 (2023), Adv. Funct. Mater. 2023, 33, 2303787 and Journal of Luminescence 263 (2023) 120114). Some also investigated the environmental stability under humid air (Nat. Photon. 18, 363–370 (2024) and Angew. Chem. Int. Ed. 2025, e202419746). Therefore, we hold an opposite viewpoint that shelf stability test under dry air is meaningful and researchers do care about that.

Reviewer #2 (Remarks to the Author):

Fan et al. have solved my previous concerns. Published as is.

Response: We would like to thank the reviewer for the positive comments and support.

Reviewer #3 (Remarks to the Author):

I am fairly satisfied with the authors response to my comments. I recommend this work for publication in Nature Communication. However, I suggest authors to revise their figures and replace with high quality and legible ones. The lables, and legens are hard to read.

Response: We appreciate the positive comments and support from the reviewer. We will provide legible and high-quality figures in a PPT file.

Response to the Reviewers' Comments

[Nature Communications NCOMMS-24-55954-B]: Ultrapure and efficient electroluminescence in alkali metal doped inorganic perovskite quantum wires arrays

We would like to thank the reviewers and the editors for their comments on our work. Such productive feedback from the experts in this field helped us to further improve our manuscript. Here we provide a point-by-point response below to the reviewers' questions. We also carefully revised our manuscript to comply with the reviewers' comments and have highlighted the revised parts in red.

Reviewer #1 (Remarks to the Author):

The author believes that what they are dealing with is a quantum wire, but I still believe this is incorrect, as evidenced by the author's own spectral characterization. Unfortunately, I cannot recommend the acceptance of this manuscript.

Firstly, if it was truly a quantum wire, the first-order exciton radiation recombination would be dominate, rather than bimolecular radiation recombination. However, from the nature of PL decay curve (figure R10, in line with double exponential fitting), it is more likely that bimolecular recombination is the dominant recombination pathway.

Secondly, From the author's characterization, the first-order recombination (k1) rate appears to have slowed down compared to the bulk perovskites, which is not reasonable. There is no doubt that quantum wires are of faster first-order recombination rates, due to the contribution from exciton recombination.

Additionally, for quantum materials with quantum or dielectric confinement, Auger recombination (k3) is typically much greater than in bulk materials. This is determined by the photophysical nature, that is, the enhanced exciton-exciton interactions.

Besides, I find it hard to believe that Auger recombination in perovskites could reach a level of 10^{-30} cm^{-6} s^{-1}, as this is slower than what is observed in current perovskite photovoltaics or single-crystal materials, making it, in my view, implausible.

I believe the author is, on the one hand, parroting others, and on the other hand, while I acknowledge that some of their previous articles demonstrated clear evidence of confinement, such confinement is absent here, and thus it does not meet the definition of a quantum wire. The author likes to use TEM to prove their nanostructures, but as I mentioned before, TEM is not comprehensive. From some of the localized region, it appears to be a nanowire or with nanostructure. However, if viewed globally, the perovskite material observed from this angle may extend over a much larger area, allowing charge carriers to move across a significantly broader region. Anyway, the results provided by optical methods are more comprehensive and accurate.

Response: We sincerely appreciate the reviewer for providing critical and insightful comments that have prompted us to thoroughly examine our data. Without these comments, we would never have had the opportunity to correct our optical measurement process and the interpretation of charge carrier dynamics. These comments have been instrumental in refining our paper, enhancing its scientific depth and clarity. We hope the reviewer can find our substantially revised paper suitable for publication.

After receiving comments from reviewer 1, the editor consulted the original reviewer 3 for additional feedback regarding reviewer 1's concerns. Reviewer 3 echoes reviewer 1's concerns and highlights that Figure S10 displays a weak excitonic absorption peak (indicating weak confinement) in the absorption, which seems to act like a bulk instead of a quantum confinement structure. After reading this comment, we have reviewed the absorption spectrum in Supplementary Figure 10 and our optical absorption measurement process. We discovered that the excitonic absorption peak is notably absent in our previous absorption spectrum of blue-emitting PeQWs. And we found it is **a result of an erroneous optical absorption measurement procedure**. We sincerely apologize for the misstates we made. Below we provide a detailed explanation on this.

Firstly, we fabricated new AAO templates and performed the optical absorption measurements. As can be seen from the Figure R1, the absorption spectrum exhibits significant variation

within the 400 nm to 800 nm range for different AAO templates fabricated in a single batch. According to literature, during the fabrication of AAO templates, there are anion impurities in AAO templates due to the competition between the water-splitting reaction and dissociation of acids to form conjugate base anions, which can replace O^{2-} in the oxide as substitution or contamination impurities (Journal of Membrane Science 98, 131-142 (1995), Microporous and Mesoporous Materials 100, 154-159 (2007) and Microporous and Mesoporous Materials 225, 192-197 (2016)). The anion has played key roles in the refractive index and absorption coefficient of the AAO templates (Microporous and Mesoporous Materials 100, 154-159 (2007) and Microporous and Mesoporous Materials 225, 192-197 (2016)). Therefore, **the significant variation in the absorption spectrum can be attributed to the variation in the anion impurities and morphology of the AAO templates**.

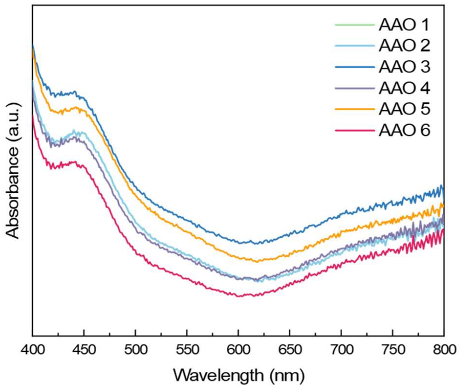

Figure R1. Absorption spectrum of 6 different AAO templates fabricated in a single batch.

Previously, when conducting optical absorption spectrum measurements, we used PeQWs in an AAO template as a target sample and another blank AAO template as a blank control sample. The actual absorption spectrum of PeQWs was determined by subtracting the absorption spectrum of PeQWs in AAO template from the absorption spectrum of the control blank AAO template. As there is a large variation in optical absorption property from AAO template to AAO template, the optical absorption of the AAO hosting PeQWs may differ much from the

control AAO sample. This can potentially cause large measurement error. To address this error, we optimized the optical absorption measurement procedure. First, we fabricated a blank AAO template and measured its absorption spectrum. Next, we deposited PeQWs into the same AAO template and measured its absorption spectrum again. The actual absorption spectrum of PeQWs is obtained through subtracting their respective absorption spectrum. We have conducted this experiment on **6 different samples**, as shown in Figure R2 and R3 below. Now, **we can clearly observe distinct excitonic absorption peaks in the absorption spectrum of each PeQWs, indicating that that our PeQWs experience quantum confinement**. The obtained results are consistent with those reported in the existing published paper (Chem. Mater. 2016, 28, 18, 6450–6454).

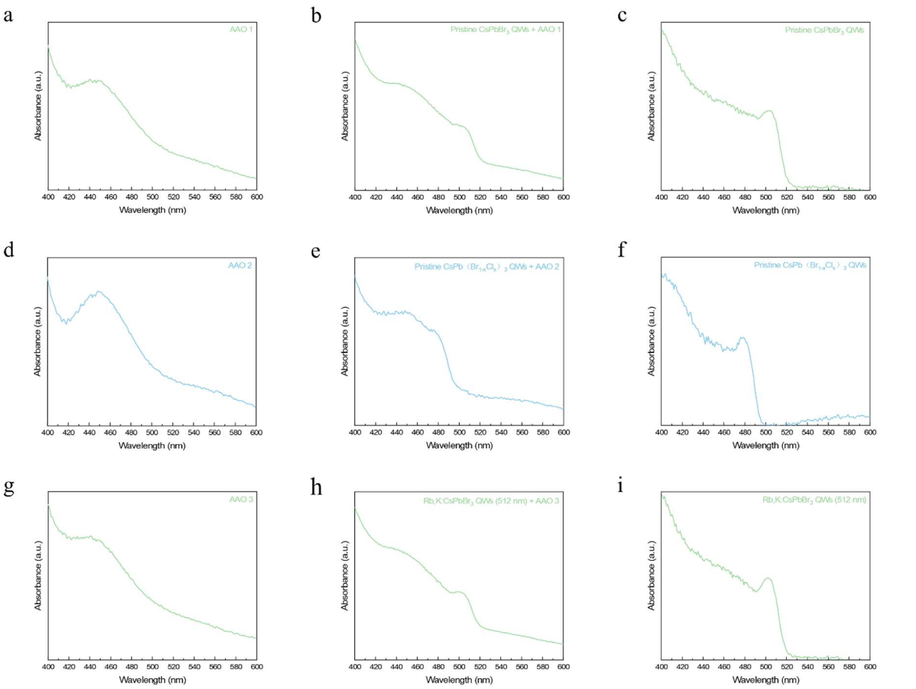

Figure R2. Absorption spectrum of a, d, g, blank AAO template 1,2 and 3, b, e, h, PeQWs in AAO template 1,2 and 3, and c, f, i, PeQWs.

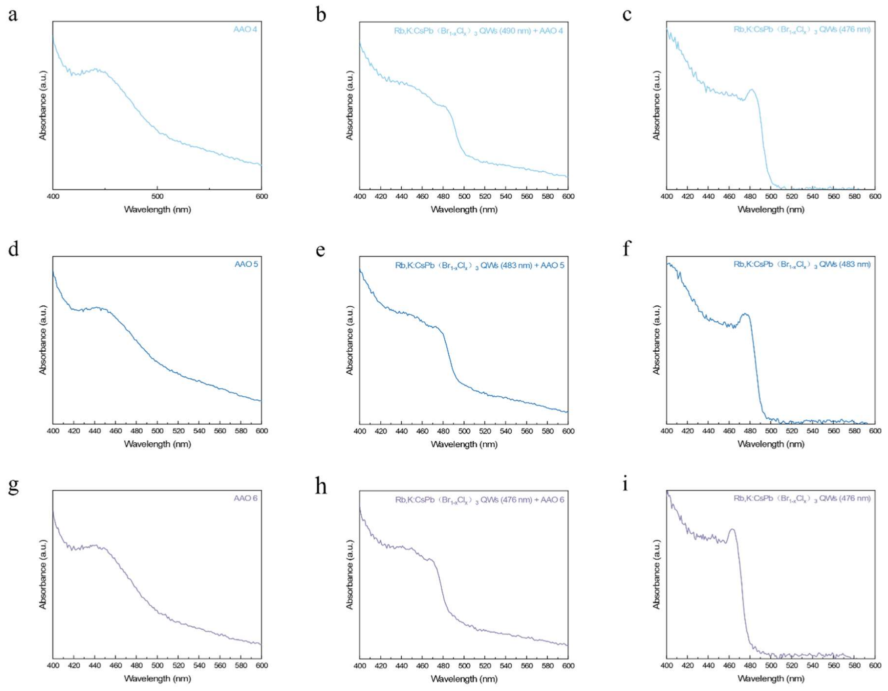

Figure R3. Absorption spectrum of a, d, g, blank AAO templates 4,5 and 6, b, e, h, PeQWs in AAO template 4,5 and 6, and c, f, i, PeQWs.

In addition, thanks to the reviewer for pointing out the recombination constant issue. We have carefully checked the results again and found that we have mis-interpreted the charge carrier dynamics previously. Below please find a detailed explanation and followed by a more accurate interpretation.

Firstly, the photo-generated carrier density (N_0) is evaluated by following equation:

$$N_0 = \frac{E\lambda\alpha(\lambda)}{hcA_{eff.}}(1 - R_{pump})$$

where E is the energy of the excited pump pulse; λ, $\alpha(\lambda)$ and R_{pump} represents the wave length, absorption coefficient and reflectivity of the excited pump pulse, respectively. $A_{eff.}$ is the efficient overlap area of the pump and the probe beam. Previously, we used the incorrect

absorption coefficient values when calculating the photo-generated carrier density (N_0) of PeQWs. There are two reasons why those values were incorrect. **Firstly, the absorption spectrum collected through the erroneous absorption measurement procedure leads to incorrect absorption coefficient values, as we mentioned above. Secondly, when performing the transient absorption measurement, we were measuring the combination of PeQWs and AAO template. Therefore, using the absorption coefficient values extracted from the absorption spectrum of PeQWs with AAO template is more reasonable for the photo-generated carrier density. However, we extracted the values from the actual absorption spectrum of PeQWs (The actual absorption spectrum is the result of the absorption spectrum of PeQWs with AAO template subtracting the absorption spectrum of the corresponding AAO template).** After correction, the calculated photo-generated carrier density (N_0) values are accurately depicted in Figure R4. Specifically, at low carrier density (usually below 10^{15} cm^{-3}), the main leakage of carriers comes from excitonic radiative recombination and trap-assisted Shockley–Read–Hall (SRH) nonradiative recombination. At medium carrier density (usually ~10^{15} to ~10^{17} cm^{-3}), bimolecular recombination appears to be the dominant mechanism (Trends in Chemistry 3, 34-46 (2021)). Given that the photo-generated carrier density (N_0) exceeds 10^{15} cm^{-3} in our materials, it is evident that excitonic radiative recombination and trap-assisted Shockley–Read–Hall (SRH) nonradiative recombination are not the primary recombination pathways in Figure R4. In the past, **instead of selecting the TA bleaching kinetics at emission wavelength measured under various pump fluences, we selected the TA bleaching kinetics at the wavelength with highest ΔA value at different pump fluences previously**. It's unfortunately incorrect and we have now plotted the correct TA bleaching kinetics at emission wavelength in Figure R4.

Commented [ZF1]: Is it the AAO variation issue we mentioned above?

Commented [22R1]: Yes

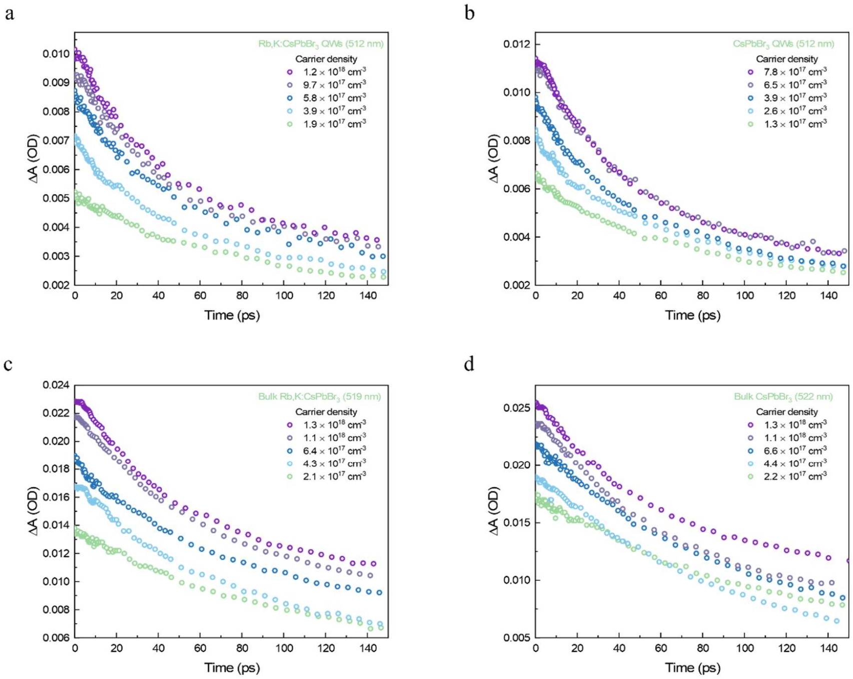

Figure R4. The TA bleaching kinetics at emission wavelength measured under various pump fluences for bulk $CsPbBr_3$ and $CsPbBr_3$ QWs with or without alkali metal doping.

Secondly, the global fits to transient absorption dynamics are illustrated as follows (Nature Communications 12, 336 (2021)). The recombination dynamics of the charge-carrier density $N(t)$ can be described in the form of:

$$\frac{\mathrm{dN(t)}}{\mathrm{dt}} = -\mathrm{k_1 N} - \mathrm{k_2 N^2} - \mathrm{k_3 N^3}$$

where k_1 is the monomolecular recombination decay constant (which is relevant to both trap-assisted recombination and geminate recombination of exciton); k_2 is the bimolecular recombination decay constant (which is related to radiative recombination); and k_3 is the Auger recombination decay constant.

Generally, the observed quantity of photo-generated transmission change $\mathrm{x(t)} = \frac{\Delta \mathrm{T}}{\mathrm{T}}\mathrm{(t)}$ shows

a linear relationship related to the charge-carrier, which can be described by the following equation:

$$N(t) = \varphi C x(t)$$

where φ is the photo-to-carrier conversion ratio, $C = \frac{N_0}{x(0)}$ is the proportionality factor between the initial transmission change $x(0)$ and the initially absorbed photon density N_0.

Combining the Supplementary equations above, we obtain the following equation:

$$\frac{dN(t)}{dt} = -k_1 x - C\varphi k_2 x^2 - C^2\varphi^2 k_3 x^3$$

Previously, we studied some papers that include analysis of fluence-dependent transient absorption spectrum. Most of them only mentioned that the first order recombination constant (k_1) is related to rap-assisted Shockley–Read–Hall (SRH) nonradiative recombination (Nat. Photon. 17, 435–441 (2023) and Nat Commun 16, 927 (2025)). That's why **we didn't take geminate exciton recombination into consideration when evaluating the first order recombination constant (k_1)**. However, this appeared to be inappropriate here.

We thank the reviewer for having inspired us to think it through again. Now, we have gone through the calculation process again. The constant values were extracted from TA measurements for different fluences with each symbol representing one measurement (Figure R5). The normalized TA kinetics were multiplied with the initial photo-generated carrier density N_0, and taking the time derivative of this carrier density N(t). We can observe different scaling of the recombination rate dN/dt with N, indicating transitions between different recombination mechanisms. The recombination coefficients are obtained by global fitting of TA dynamics at different excitation intensities and summarized in Table R1. According to a published review paper (Trends in Chemistry 3, 34-46 (2021)), **the value of k_1 ranges from ~10^5 to ~10^9 s^{-1}, the value of k_2 varies from ~10^{-12} to ~10^{-7} cm^3 s^{-1} and the Auger recombination rate coefficient k_3 of most perovskites usually ranges from 10^{-26} to 10^{-29} cm^6 s^{-1}**. Thus, the obtained constant values in Table R1 fall within the normal range. Similar to the results in another paper (Nat Commun 8, 14558 (2017)), the k_1 of PeQWs is two orders of

magnitude higher than that of bulk perovskite. The k_1 of PeQWs is dominated by geminate exciton recombination while the k_1 of bulk perovskite is dominated by trap-assisted Shockley–Read–Hall (SRH) nonradiative recombination. Moreover, the PeQWs exhibit higher radiative recombination rate compared with bulk perovskite. Because of enhanced exciton-exciton interactions mentioned by the reviewer, the obtained Auger recombination rate of PeQWs is one order of magnitude higher.

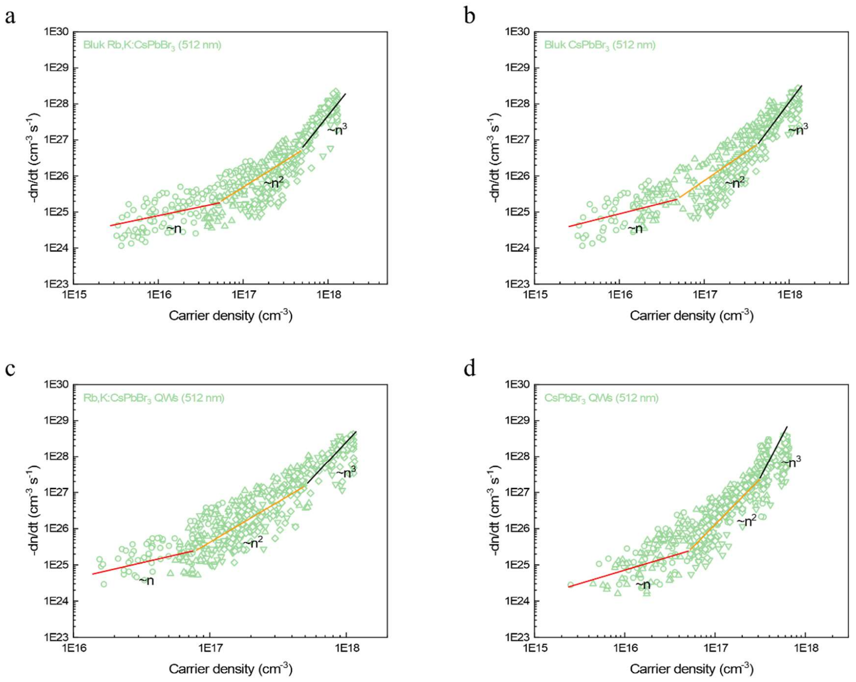

Figure R5. Recombination rate over charge density of bulk $CsPbBr_3$ and $CsPbBr_3$ QWs with or without alkali metal doping.

Table R1. Monomolecular (k_1), bimolecular (k_2) and trimolecular (k_3) recombination constant of bulk $CsPbBr_3$ and $CsPbBr_3$ QWs with or without alkali metal doping.

	k_1 (s^{-1})	k_2 (cm^{-3} s^{-1})	k_3 (cm^{-6} s^{-1})

Bulk $CsPbBr_3$ (522 nm)	4.9×10^6 (Trap-assisted)	1.3×10^{-8}	5.3×10^{-27}
Bulk Rb, K:$CsPbBr_3$ (519 nm)	2.4×10^6 (Trap-assisted)	8.4×10^{-9}	2.4×10^{-27}
$CsPbBr_3$ QWs (512 nm)	1.3×10^8 (Excitonic)	4.3×10^{-8}	1.6×10^{-26}
Rb, K:$CsPbBr_3$ QWs (512 nm)	2.5×10^8 (Excitonic)	6.8×10^{-8}	5.1×10^{-26}

In addition, we re-analyze our TA data to further support our statement. The TA evolution in the course of carrier intraband relaxation was illustrated in a report (Nano Lett. 2016, 16, 4, 2349–2362) (Figure R6). Without excitation (at left), optical transition energies "seen" by a probe pulse are defined by single-exciton energies (solid lines). After a high-energy "hot" exciton is injected into a QD (middle), the transition energies seen by the probe pulse are modified by the exciton–exciton interaction (dashed lines). In the case of exciton–exciton attraction (shown in this example), an energy required to introduce the second exciton into the QD is reduced compared to that of the single-excition state by the exciton–exciton interaction energy. This is a so-called "biexciton effect" manifested as a red shift of the band-edge transition observed in early time TA as a derivative like feature with a photoinduced absorption (PA) at the lower energy accompanied by a bleach at the higher energy. After carriers relax into the band-edge state (at right), the PA feature is overwhelmed by the bleach signal resulting from the state-filling effect.

[FIGURE REDACTED]

Figure R6. A schematic illustration of TA evolution in the course of carrier intraband relaxation (Nano Lett. 2016, 16, 4, 2349–2362).

Therefore, we can clearly observe a noticeable positive PA at lower energy in the early time transient absorption spectrum (Figure R7). **An earlier study pointed out that exciton–exciton interaction energy increases with decreasing the size of perovskite NCs, indicating that perovskite experiences quantum confinement** (Chem. Mater. 2017, 29, 8, 3644–3652). The increased exciton–exciton interaction energy in perovskite NCs with smaller sizes indicates a heightened excitonic nature which caused by the combination of increased Coulomb interactions with decreasing particle size, easier crystal lattice deformation, or reduced screening from ionic nuclei effect in smaller NCs. Similarly, we extracted the exciton–exciton interaction energy from the transient absorption spectrum listed in TableR2. We obtained similar values to those reported in the mentioned paper, indicating that our PeQWs experience quantum confinement.

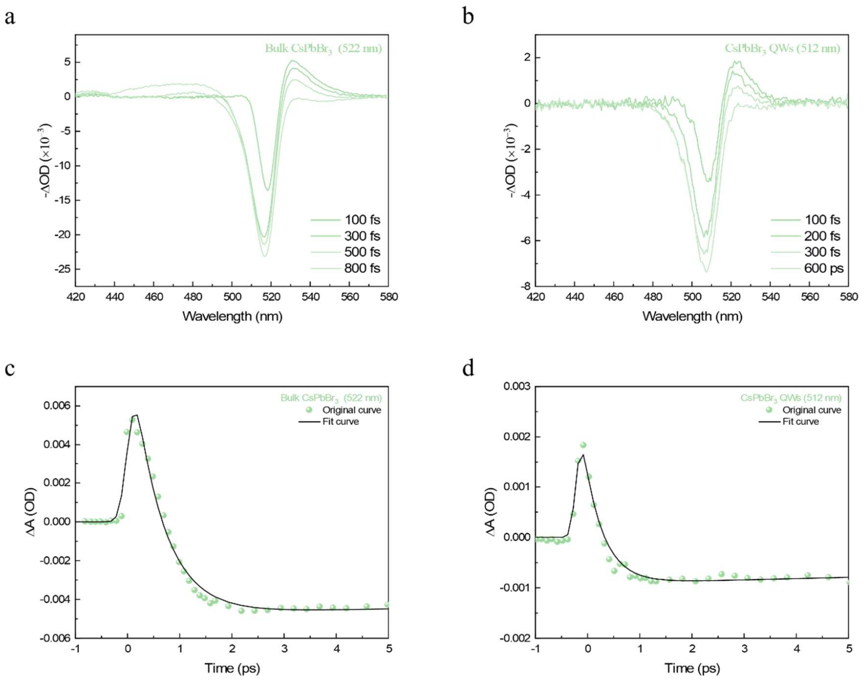

Figure R7. a, b, Early time carrier dynamics in bulk perovskite and PeQWs probed by transient absorption spectroscopy. c, d, Early time TA dynamics probed at the positions of the PA.

Table R2. Exciton–exciton interaction energy and short time cooling lifetimes

	Exciton–exciton interaction energy (meV)	τ_{cool} (fs)
Bulk $CsPbBr_3$ (522 nm)	54.0	574
$CsPbBr_3$ QWs (512 nm)	74.5	376

Regarding the TEM images of our PeQWs, the reviewer asserted that the perovskite material may extend over a much larger area when viewed globally. We assume that the reviewer considers the material within the two white dash lines to be a perovskite nanowire embedded in a larger perovskite which is located within the two red dashed lines (Figure R8. a and b).

Consequently, the charge carriers can move freely within this region, causing perovskite to exhibit characteristic of bulk material. It’s totally reasonable to hold this perspective since we didn't offer an explanation for it before. However, the actual situation may differ from the reviewer's impression. The fabricated AAO template with an average pitch of 12 nm (the distance between the center of two pores) has separated channels that are not interconnected (Figure R8. e). After perovskite deposition, isolated PeQWs surrounded by insulating aluminum oxide form inside the channels, confining the charge carriers within individual PeQW. It’s impossible for charge carriers to more freely across different PeQWs because of the existence of insulating aluminum oxide. In cross-sectional TEM images, the detected materials, devoid of a visible crystal lattice, within the two red dash lines are isolated PeQWs. When taking the TEM images, we only focused on one single PeQW. To further prove that our materials are isolated PeQWs, we extracted a single PeQW using the method outlined in our previous reports (Nano Lett. 2019, 19, 5, 2850–2857 and Nat. Photon. 16, 284–290 (2022)). We can clearly observe extracted single PeQW with diameter of ~6.4 nm in the Figure R8. c and d.

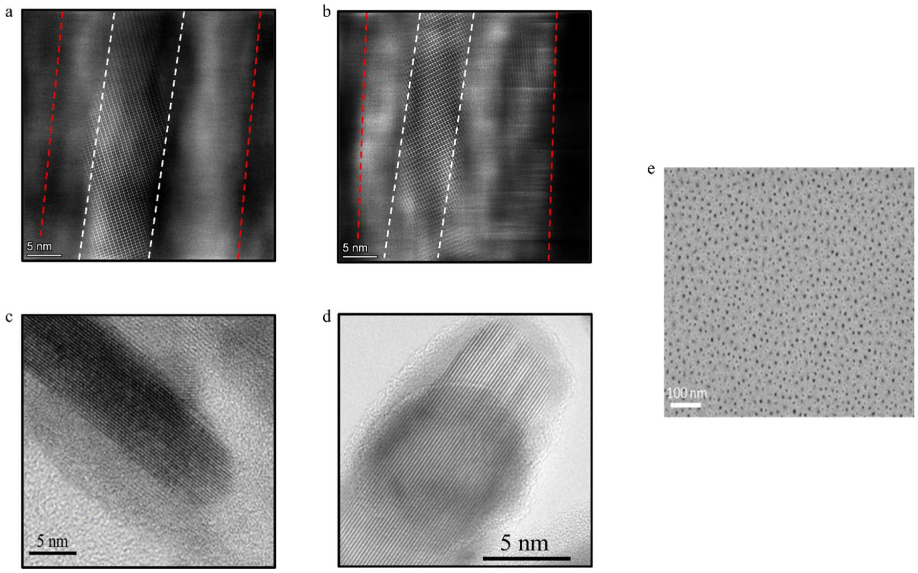

Figure R8. a, b, Cross-sectional TEM images of PeQWs. c, d, TEM images of extracted single

PeQW. e, Top-view SEM image of AAO template.

Based on the aforementioned explanation, we have achieved more reliable results and made corresponding corrections in terms of the critical insights from the editors and reviewers. And we believe that our material are perovskite quantum wires with quantum confinement. We would like to express our gratitude to the reviewer for highlighting the clear mistakes we made during the last review. We hope that the reviewer will reconsider the possibility of our work being accepted by Nature Communications.

Revision: Page 10, line 215-223 in the revised manuscript. Supplementary Figure 10, Supplementary Figure 20 and Supplementary Table 1 in the revised supplementary information.

Response to the Reviewers' Comments

[Nature Communications NCOMMS-24-55954-C-Z]: Ultrapure and efficient electroluminescence in alkali metal doped inorganic perovskite quantum wires arrays

We would like to thank the reviewers and the editors for their comments on our work. Such productive feedback from the experts in this field helped us to further improve our manuscript. Here we provide a point-by-point response below to the reviewers' questions. We also carefully revised our manuscript to comply with the editor' comments.

Reviewer #1 (Remarks to the Author):

I appreciate the authors' efforts in investigating carrier dynamics and acknowledge that their conclusion—excitonic recombination dominance—could support the achievement of quantum wires. Although I remain somewhat puzzled by how the authors arrived at results that appear to completely contradict their previous findings within such a short timeframe and using identical technology, I see no reason to reject this manuscript.

However, I remain unconvinced by the shelf lifetime data (Fig. 3e), as I said it is not meaningful. I guess the authors are not willing to show their short operational lifetime in their main context, and thus use the shelf lifetime instead. Given the high impact of Nature Communications, I am concerned that the current presentation of these results may potentially mislead the perovskite research community.

Response: We sincerely appreciate the reviewer for providing critical comments. Previously, our study on the charge carrier dynamics based on fluence-dependent transient absorption was not comprehensive enough. Upon the reviewer pointing out the recombination constant issue, we promptly delved into studying fluence-dependent transient absorption and verified the previous results. After more systematic study, we have come to the conclusion that we have mis-interpreted the charge carrier dynamics previously. In the previous response, we are confident that we have provided a detailed explanation and a more accurate interpretation.

Regarding the shelf lifetime data, we believe that we have already offered a clear explanation for why we present this data in the previous response. Additionally, we mainly demonstrated the properties of our PeQWs in the Figure 3 and there is no intention to mislead in presenting the PL lifetime of our PeQWs under ambient condition in this section. As for the operational lifetime of our devices, we have mentioned it in the main context and presented the corresponding figure in the supplementary information (Supplementary Fig. 31). We have no intention of hiding it.

Reviewer #3 (Remarks to the Author):

I think authors demonstrated extensive studies to prove that they are synthesizing perovskite quantum wires. Their absorption data both steady-state and transient now shows convincing evidence of nanowire formation. I strongly encourage the authors to replace both Absorption and TA data in the SI with high resolution figures. Overall, I am satisfied with authors response and I recommend this work for publication in Nat comm.

Response: We appreciate the positive comments and support from the reviewer. We will provide legible and high-quality figures.
